# Dissecting a novel allosteric mechanism of cruzain: A computer-aided approach

**DOI:** 10.1371/journal.pone.0211227

**Published:** 2019-01-25

**Authors:** Lilian Hernández Alvarez, Diego Enry Barreto Gomes, Jorge Enrique Hernández González, Pedro Geraldo Pascutti

**Affiliations:** 1 Departamento de Física, Instituto de Biociências, Letras e Ciências Exatas, Universidade Estadual Paulista Julio de Mesquita Filho, São José do Rio Preto, São Paulo, Brazil; 2 Diretoria de Metrologia Aplicada às Ciências da Vida (DIMAV), Instituto Nacional de Metrologia, Qualidade e Tecnologia (INMETRO), Rio de Janeiro, Brazil; 3 Institute de Chimie, Université de Strasbourg, Strasbourg, France; 4 Instituto de Biofísica Carlos Chagas Filho, Universidade Federal do Rio de Janeiro (UFRJ), Rio de Janeiro, Brazil; Wake Forest University, UNITED STATES

## Abstract

*Trypanosoma cruzi* is the causative agent of Chagas disease, a neglected infection affecting millions of people in tropical regions. There are several chemotherapeutic agents for the treatment of this disease, but most of them are highly toxic and generate resistance. Currently, the development of allosteric inhibitors constitutes a promising research field, since it can improve the accessibility to more selective and less toxic medicines. To date, the allosteric drugs prediction is a state-of-the-art topic in rational structure-based computational design. In this work, a simulation strategy was developed for computational discovery of allosteric inhibitors, and it was applied to cruzain, a promising target and the major cysteine protease of *T*. *cruzi*. Molecular dynamics simulations, binding free energy calculations and network-based modelling of residue interactions were combined to characterize and compare molecular distinctive features of the apo form and the cruzain-allosteric inhibitor complexes. By using geometry-based criteria on trajectory snapshots, we predicted two main allosteric sites suitable for drug targeting. The results suggest dissimilar mechanisms exerted by the same allosteric site when binding different potential allosteric inhibitors. Finally, we identified the residues involved in suboptimal paths linking the identified site and the orthosteric site. The present study constitutes the first approximation to the design of cruzain allosteric inhibitors and may serve for future pharmacological intervention. Here, no major effects on active site structure were observed due to compound binding (modification of distance and angles between catalytic residues), which indicates that allosteric regulation in cruzain might be mediated via alterations of its dynamical properties similarly to allosteric inhibition of human cathepsin K (HCatK). The current findings are particularly relevant for the design of allosteric modulators of papain-like cysteine proteases.

## Introduction

Cruzain is the major papain-like cysteine protease of *Trypanosoma cruzi*, the protozoan responsible for Chagas disease. This enzyme is indispensable for the survival and propagation of the parasite and, therefore, is considered a potential drug target for the disease control [1–3]. Toxicity and inefficiency of the available chemotherapy [4–6], fueled the pursuit for alternative drugs, which in turn, has led to the discovery of many cruzain inhibitors. A tangible evidence of the latter, is the occurrence of twenty-five crystal structures of cruzain in complex with different competitive inhibitors in the Protein Data Bank (PDB) [1, 7–9]. On the other hand, numerous experimental studies and computational predictions have been performed to characterize the cruzain binding site and specificity, facilitating the design of active-site directed drugs [8–12]. However, the lack of interest of pharmaceutical industry in low profit products (which is the case of tropical diseases) [13], and the failure to find a “real drug” that reaches the production and distribution scales, have encouraged the search for new scaffolds of cruzain inhibitors, as well as different strategies of enzyme inhibition and, ultimately, novel therapeutic targets.

Allostery has usually been defined as the modulation of the protein function triggered by the interaction with a molecule (allosteric modulator) through a protein site (allosteric site) different from the functional one (orthosteric site) [14, 15]. Remarkably, it has been shown that allostery is not only a property of proteins, but also an inherent characteristic of most macromolecules [16, 17]. Since the proposal of the concerted Monod, Wyman, and Changeux (MWC) [18] and the sequential Koshland, Nemethy, and Filmer (KNF) models [19] in the 1960s, conformational changes were considered as a signature of allosteric modulation. However, a more modern concept of allostery takes into account the “conformation ensembles and population shift” rather than the classical two state model [20–24]. According to this concept, macromolecules exist as a population of equilibrium conformations, which has a certain dynamic distribution that may be altered by some environmental changes, such as covalent or noncovalent binding of ligands, pH, temperature, or ionic force [25]. The redistribution of the fraction of active/inactive protein conformations within the population promoted by the binding of an allosteric ligand leads to the modulation of the protein function [16, 25, 26].

In recent decades, the design of allosteric drugs has emerged as a promising research field in disease control [14, 15, 27]. Allosteric modulators offer many advantages that make them appropriate as drug candidates. A major benefit of these strategies, compared to those that perturb the active site directly, is that they offer a noninvasive or more specific protein control. Allosteric modulators neither interfere with the active site ligands nor do they obstruct its vicinity [17, 25, 28].

**Fig 1:**
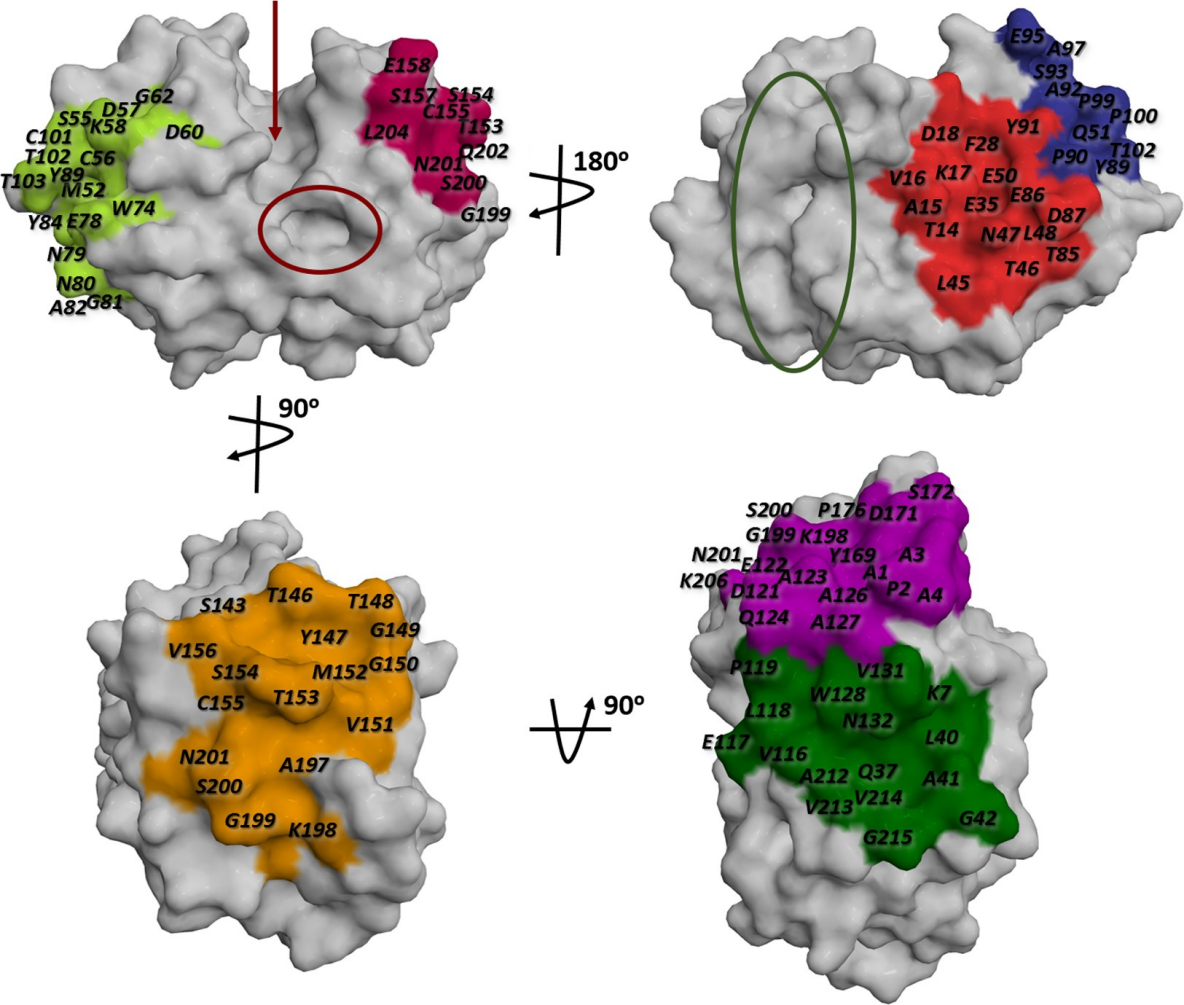
Mapping of cruzain potential allosteric pockets based on previous predictions for papain-like cysteine proteases superfamily. The allosteric sites were numbered from zero to seven, i. e., site 1 (lemon), site 2 (pink), site 3 (red), site 4 (blue), site 5 (orange), site 6 (violet) and site 7 (green). The position of the cruzain active site is indicated with an arrow and an adjacent subsite identified by Durrant *et al*. [11] is enclosed by red circle. The site 0 (glycosaminoglycan binding site in HCatK) is highlighted with a green circle.

Several findings support the existence of allosteric modulation in the papain-like proteases [29–35]. One key example is the work of Novinec *et al*., where several allosteric sites of human cathepsin K (HCatK) and the sector residues potentially mediating allosteric communication in the aforementioned protein family were identified employing the statistical coupling analysis (SCA) method (Fig 1) [36]. Both, in the previous work and in a more recent study, the co-crystallization of HCatK with allosteric inhibitors, positioned in a previously-predicted site 6, was reported (PDBID: 5J94 and 5JA7) [36–38]. On the other hand, there are other cases of allosteric modulation in several parasitic cysteine proteases such as falcipain 2 from *Plasmodium falciparum*, which is allosterically inhibited by heme, suramin and their derivatives, as well as a chalcone-like compound [33–35].

Interestingly, the role of glycosaminoglycans (GAGs) in allosteric modulation of papain-like cysteine proteases has been widely studied in the past decades. Proteases such as human cathepsins S (HCatS) and B (HCatB), brucipain, cathepsin L of *Leishmania mexicana* and even cruzain are allosterically modulated by GAGs [30, 32, 39–43]. However, no other biomolecules or chemical compounds exerting allosteric modulation on cruzain have been reported. So far, there is only an *in silico* work by Durrant *et al*. reporting the characterization of an adjacent subsite to the cruzain orthosteric site (Fig 1) [11]. However, it is expected that the predicted site will be useful for the design of more specific competitive inhibitors, not allosteric ones.

In recent years, the computer-aided drug design has been widely used in the field of rational discovery of allosteric inhibitors [37, 44–46]. Techniques such as SCA, molecular docking, molecular dynamics (MD) simulations and allosteric networks have emerged as a valuable complement to experimental methods for the study of allostery [16, 24, 47–49]. These approaches have also allowed the prediction of allosteric sites and the quantification of protein and/or ligand motions in full atomic detail, describing the molecules behavior at high resolution [28, 50–52].

Among the current *in silico* methodologies employed to study allostery, those based on the dynamical network analysis have proven particularly useful [24]. The latter approaches take advantage on the possibility that subtle adjustments in protein dynamics induce modifications of correlated-residue motions, thereby fine-tuning the signal propagation within the protein structure [24]. An insightful way of extracting information from the dynamical network representations is by clustering its nodes into communities, i.e., groups of highly intracorrelated residues that are loosely correlated with the remaining residues [53]. This analysis can further our comprehension on the dynamic perturbations triggered by the allosteric modulator in order to propagate the allosteric signal across the protein structure. In addition, residues that are crucial to the allosteric mechanism can be identified, thus providing a powerful way for intervention through mutagenesis [24, 53].

Here we present a combination of computational approaches, such as virtual screening (VS), MD simulations, binding free energy calculations and structure-based network analysis to propose novel allosteric sites and modulators of cruzain. To our knowledge, this is the first work where all crystal structures reported for this protease are compared in order to explore the diversity of its conformational space. An ensemble VS was conducted to cope with the flexibility of allosteric sites in the docking score. Moreover, a graph-based representation of protein structures was employed to investigate the correlated motions, allosteric propensities of protein residues and organization of residue-residue interaction networks. In general, we provide molecular-level insights into protein dynamics, transient pocket formation and enzymatic function, caused by ligand binding to the cruzain allosteric sites. The approach presented here provides a framework for the *in silico* design of allosteric drugs targeting this protease and other enzymes.

## Materials and methods

### Virtual screening

298 709 compounds from the files 21_p0.0.sdf and 21_p0.1.sdf contained in Lead Now library of ZINC database (http://www.zinc.org, accessed on March, 30^th^, 2016) were filtered in order to eliminate the potentially reactive and non-lead compounds. After this step, the protonation states of the remaining 209 422 compounds were defined according to the following criteria: *i*) all basic groups were protonated and *ii*) all acid groups were deprotonated. For simplicity, no tautomers were included in our compound library. Subsequently, each compound was parametrized using the MMFF94 force field and subjected to energy minimization (EM) until convergence, defined by an energy gradient tolerance of 0.05 kcal Å^-1^ mol^-1^. All previous steps were conducted with MOE platform [54]. Finally, the compound database was converted into pdbqt format with AutoDockTools [55].

**Fig 2:**
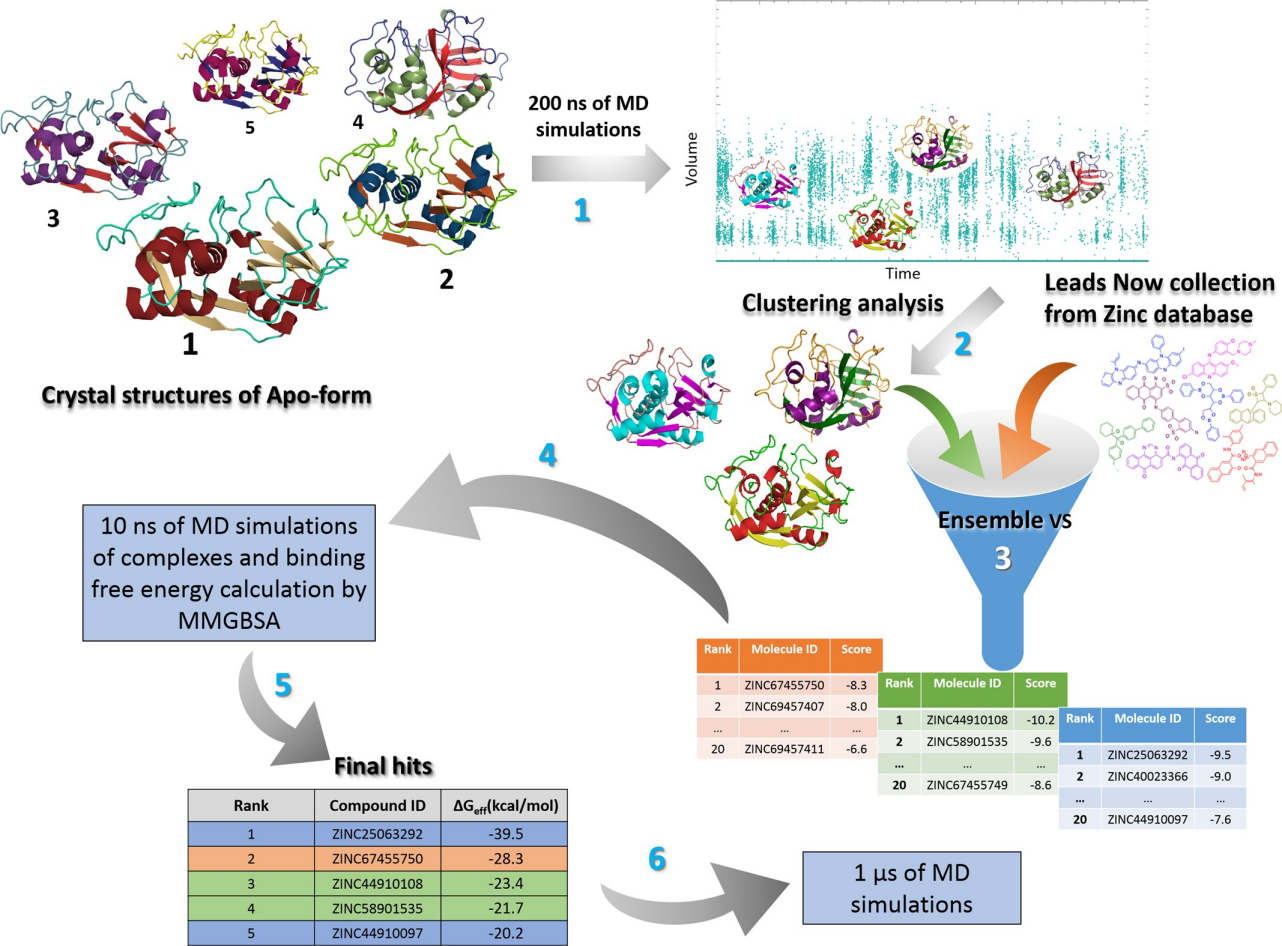
Workflow of structure-based identification of cruzain allosteric inhibitors. In the first step, five cruzain apo structures were selected and subjected to 200 ns of MD simulations. The second step represents the volume-based cluster analysis performed for each allosteric site, which produced three representative structures per pocket. The third step illustrates the ensemble VS accomplished for each cavity, where twenty hits were chosen per representative structure (60 per allosteric site). Subsequently, 10 ns MD simulations were carried out with these 60 complexes and the respective trajectories were employed for MM-GBSA binding free energy calculations. In this step, a final list of five hit compounds, ranked according to the MM-GBSA results, was obtained per allosteric site. Finally, 200 ns MD simulations were conducted to analyze the potentially mechanism of cruzain allosteric modulation exerted by these compounds.

VSs against the three representative structures of each selected allosteric site (Fig 2, step 3) were carried out in parallel with AutoDock Vina v1.12 software [56]. The number of generated poses was set to ten and docking simulation were initiated using a random seed. The exhaustiveness of the search and energy range between the best and the worst binding modes were set to the default values (8 and 3 kcal/mol, respectively) The grid box was defined according to the size of each cavity (S1 Fig), using AutoDockTools [55]. Twenty complexes with the highest Vina-score pose (S_vina_) were selected per representative structure, thus generating 60 preliminary hits for each cavity (Fig 2, step 3). Subsequently, all these complexes were subjected to short MD simulations in order to re-score binding poses using Molecular Mechanics Generalized Born Surface Area (MM-GBSA) free energy calculation (Fig 2, step 4).

### Ligand parametrization

Ligand parametrization was required in order to perform MD simulations of cruzain complexes. A less accurate parametrization using the AM1-BCC method [57] implemented in Antechamber was used for re-ranking the compounds selected from VS (Fig 2, step 4). After this intermediate rescoring step, a most rigorous charge derivation was conducted for the most promising compounds (e.g., top 5 compounds). The compounds selected for long simulations (Fig 2, step 6) underwent steepest descents EM until gradient convergence with the Universal Force Field (UFF) parameters using Avogadro [58]. The minimized structures were subsequently subjected to geometry optimization at B3LYP/6-31G(d,p) level using Gaussian 09 package [59]. The electrostatic potential (ESP) for each optimized structure was then generated by single-point (SP) calculations using the HF/6-31G(d) method and Merz-Kollman (MK) scheme [60]. The partial atomic charges were fitted to the ESPs through the Restricted Electrostatic Potential (RESP) fit implemented in the program antechamber of Amber14 package [61]. Finally, the compounds were fully parametrized using the previously-determined partial charges and assigning the remaining parameters according to the Generalized Amber Force Field (GAFF) [62] implemented in Amber14 package [61].

### Protein structures retrieval and systems setup and minimization

Twenty-five tridimensional (3D) structures of cruzain in complex with competitive inhibitors were obtained from Protein Data Bank (PDB) [63]. Subsequently, all cruzain structures (including each protein chain reported in each PDB file) were compared with each other through the calculation of pairwise Root Mean Square Deviation (RMSD) of the backbone atoms. This analysis was performed in order to find structures with highest structural differences after removing heteroatoms (Fig 2, step 1). Other parameters taken into account for the selection of cruzain structures were the experimental resolution (<2.5 Å) and their usage in previous computational experiments, for comparison purposes (see S1 Text). The numbering scheme for cruzain residues was taken from their sequential order of appearance in the protein sequence (not the papain-numbering scheme present in PDB structures).

Protonation states of cruzain ionizable residues were determined at the main functional pH of the enzyme (pH = 5.5) using the PDB2PQR server [64]. Further preparation and simulations were performed with Amber14 [61]. Systems were solvated with explicit TIP3P water molecules [65] in a cubic box extending at least 10 Å from the solute surface, and treated with periodic boundary conditions. Negative net charges were neutralized by replacing water molecules with Na^+^ counterions. AMBER14SB force field [66] was selected for protein parametrization. EM was performed with pmemd.MPI program of Amber14 package [61]. This procedure was conducted in two steps: (*i*) solute atoms restrained with a harmonic constant of 10.0 kcal∙mol^-1^∙Å^-2^, and (*ii*) unrestrained system. In both cases, 1500 steps of steepest descent followed by 500 steps of conjugated gradient EM were performed.

To study the potential impact of the allosteric ligands on the functionality of the orthosteric site in terms of affinity, MD simulations with a mimetic substrate of cruzain were performed. Cruzain-peptide complexes were built following the strategy reported by Durrat *et al*. [11], i.e., by superimposing the average structure from MD simulations of the cruzain apo form with the crystal structure of human procathepsin K (PDB: 1BY8). According to the available information on specificity of cruzain binding site, the propeptide of procathepsin K was mutated using the mutagenesis plugin of Pymol v1.8 [67] in order to model the structure of an optimized substrate of cruzain. Previous studies have described that cruzain “prefers” basic and hydrophobic amino acids at P1 and P2 positions, being LEU and PHE the most frequent ones at P2 [68, 69]. Another work reported that cruzipain (naturally occurring form of cruzain), accommodates preferentially basic residues at P3-P5 and P3’-P5’positions, while there is no discrimination at P1’ for residues PHE, LEU, ARG and ALA [68]. In addition, cruzain hydrolyzes substrates with SER, PHE, ALA or PRO at P2’ with similar efficiency [69]. Based on this information, the final sequence generated for the heptapeptide was ACE-AKLF↓LAK-NME, where ACE and NME stand for acetyl and N-methyl amide capping groups, added to the N- and C-termini, respectively, in order to neutralize their net charges.

### Molecular dynamics simulations

The equilibration procedure was conducted with pmemd.MPI and involved two sequential steps in the NVT and NPT ensembles, respectively, both keeping the solute heavy atoms restrained with a 10.0 kcal∙mol^-1^∙Å^-2^ restraint constant. During the 500 ps of NVT equilibration, the temperature was linearly increased from 10 to 300 K using the Berendsen thermostat [70]. The subsequent 500 ps of NPT equilibration was performed at a temperature of 300 K and a pressure of 1 bar, employing the Langevin thermostat and Berendsen barostat for temperature and pressure control, respectively [70, 71]. The particle mesh Ewald (PME) method was used to handle long-range electrostatic interactions [72] and the cut-off value for non-bonded interactions was set to 10 Å. Covalent bonds involving hydrogen atoms were constrained with the SHAKE algorithm [73]. A time step of 2 fs was used for numerical integration of the Cartesian equation of motion and coordinate files were recorded every 20 ps.

For cruzain apo-form, 200 ns of MD simulations were performed for each of five different structures selected in pairwise-RMSD analysis. Besides, the complexes involving the top-scoring ligands from the Vina-score ranking list were selected for shorter 10 ns MD simulations in complex with cruzain (Fig 2, step 4). Finally, five independent 200 ns production runs were carried out for each complex selected from MM-GBSA results (Fig 2, step 6). The pmemd.cuda program [74] was employed to run the production simulations in identical conditions to those used during NPT equilibration step but without restraints.

### Characterization and selection of cruzain cavities

Initially, each allosteric site previously-predicted by the SCA method [36] was mapped onto the cruzain structure for further analysis. Cruzain cavities were analyzed from the MD simulations of the apo form. The cavity volume must correspond to or exceed that of a ligand and the shape must allow its fit. In addition, the pocket physicochemical properties must complement those of the ligand [75]. For these reasons, the cavity volumes were calculated with POVME [76] throughout the simulation time, which provided an estimation of ligand accessibility for each cruzain identified pocket. Only cavities with a minimal volume greater than 200 Å^3^ were considered for the VS. Moreover, the most flexible cruzain regions were monitored through the calculation of per-residue Root Mean Square Fluctuation (RMSF) values. Finally, in order to characterize the electrostatic properties of each cavity, the APBS method available at PDB2PQR web-server was used to calculate the electrostatic potential on the protein surface (http://nbcr-222.ucsd.edu/pdb2pqr_2.1.1/) and the results were visualized with Pymol v1.8 [67].

To provide a non-redundant set of cruzain pocket conformations for the ensemble VS (Fig 2, step 2), ten representative conformations per pocket were generated by volume-based clustering analysis, using the hierarchical-agglomerative algorithm implemented in cpptraj [77, 78]. Furthermore, the RMSD of pocket heavy atoms was calculated for each pair of representative conformations. Therefore, we considered the structures with the highest differences in terms of both, volume and pairwise RMSD values, while ignoring the cluster size. Our final selection was limited to three representative conformations (Fig 2, step 2). The data of pocket volume and pairwise RMSD values were normalized to keep both magnitudes comparable.

### Trajectories analysis

The cpptraj module of Ambertools14 package [61, 78] was used to determine trajectory parameters such as RMSD, RMSF, average structure, interatomic distances and hydrogen bonds. The calculation of RMSD values for side chain and backbone atoms was performed employing the snapshots from production runs with respect to corresponding starting frame. Hydrogen bonds established between each ligand and cruzain were calculated with the default geometric definition of cpptraj. This geometric criteria establish a distance cutoff of 3.0 Å between acceptor and donor heavy atom, and the acceptor-hydrogen-donor angle must be ≥ 135° [61, 78]. Figures of protein cavities density and complexes were generated with Pymol v1.8 program [67].

### Binding free energy calculations

MM-GBSA and MM-PBSA are computationally efficient methods for estimating the binding free energy (Δ*G*_*bind*_) of protein-ligand complexes [79–82]. Here, the effective free energy (Δ*G*_*eff*_, neglecting the configurational entropic contribution) values of all protein–ligand complexes were calculated using the MMPBSA.py program [83]. The single-trajectory approximation was used for this calculation, employing the MD simulations corresponding to the 60 complexes selected per cavity (Fig 2, step 4). The GB-neck2 model (igb = 8) with mbondi3 radii was used for estimating the polar solvation energy (Δ*G*_*GB*_) [61]. The Δ*G*_*eff*_ values were averaged over all snapshots, thus generating a new score that allowed us to obtain a re-ranked list of the most promising candidates (Fig 2, step 5). For the final set of top-scoring compounds, average Δ*G*_*eff*_ values from simulation replicas were determined as a criterion of ligand stability into allosteric site. The standard error of the mean (*SE*) was calculated through the following equation: $SE\;=\;\frac{SD}{\surd N}$, where *SD* is the standard deviation of the average Δ*G*_*eff*_ values obtained from the independent simulations (*N*).

Finally, per-residue effective free energy decomposition was carried out in order to determine the most important residues involved in cruzain-ligand interactions [84, 85]. Energetically-relevant residues, i.e., warm- and hot-spots, at the interfaces of the studied complexes were predicted using the energy decomposition protocol implemented in MMPBSA.py and the single trajectory approach. We defined as warm- and hot-spot residues those with an energy contribution (Δ*G*_*res*_) to the total Δ*G*_*eff*_ value ranging from -1.0 to -0.4 kcal/mol and ≤-1.0 kcal/mol, respectively, as defined elsewhere [86].

### Coordination propensity calculation

The calculation of coordination propensity (*CP*) coefficient was performed with home-made scripts using the formula reported in previous works (Eq 1) [87, 88]. This methodology is based on the construction of a matrix whose elements, termed coordination propensity parameters, are calculated as follows:

$$CP_{i,j}=\langle {\left(d_{i,j}-\langle d_{i,j}\rangle \right)}^{2}\rangle$$ (1)

where *d*_*i*, *j*_ is the distance between the Cα atoms of residues *i* and *j* in each snapshot and the brackets indicate the average distance over the trajectory.

The *CP* matrices obtained from independent replicas were averaged for each system. The values obtained for the apo form were subtracted from those obtained for the holo form $\left(\langle CP_{apo}\rangle -\langle CP_{holo}\rangle \right)$ and reported for each complex.

### Graph construction and calculations

Correlated motions between residues were calculated using the general correlation (*GC*) coefficients derived from mutual information theory [89]. In this approach, protein residue networks are defined as a set of residues; i.e., nodes, connected by edges (residue pair connections weighted by *GC* values). The *GC* matrix was calculated with g_correlation software of GROMACS v3.2.1 [89]. The *GC* of each pair of residues was obtained for every system, considering the motion of Cα atoms and averaging over independent replicas for both, apo and holo cruzain systems. In order to highlight the principal changes in *GC* values of the analyzed systems, the matrices obtained for apo enzyme were subtracted from those obtained for holo complexes $\left(\langle GC_{holo}\rangle -\langle GC_{apo}\rangle \right)$.

In this work, the network construction and the processing of *GC* and contact map matrices were made with Bio3d package [90], employing the methodology proposed by Sethi *et al*. [53]. The distance used for contact maps calculation was 5.0 Å, as previously used in other studies [91], in which it produced the smallest number of communities for the studied systems. The cut-off of *GC* value taken for network building was 0.5. For every system, the pairwise product between the average *GC* and contact map matrices corresponding to each simulation replica $\left(\langle GC_{ij}\rangle \cdot \langle Cmap_{ij}\rangle \right)$ was used to generate the final average matrix employed in network community calculations. The graph community structure was built using the Girvan-Newman clustering method, which is essentially based on the edge betweenness partition criterion [92].

The path length was weighted using the *GC* coefficient according to the Eq 2 [53].

$$d_{i,j}=-log\left|GC_{i,j}\right|$$ (2)

Here, *d*_*i*, *j*_ is the distance between contacting nodes i and j. According to this analysis, the resulting graph will produce short distances for strongly correlated residues and longer distances for residues with weak correlations. The optimal paths were identified using Dijkstra’s algorithm available in NetworkX [93]. Finally, for a given pair of residues termed source and sink, 500 suboptimal pathways were calculated employing the Weighted Implementation of Suboptimal Pathways (WISP) method [94]. Residues CYS25 at the catalytic site and a hot spot of the allosteric site were selected as sink and source, respectively, for pathway analysis.

### Construction of energy interaction networks based on cruzain ensembles from MD simulations

Protein energy networks (PENs) are a specific case within the protein structural networks (PSNs) approach [48, 95–100]. PENs construction is based on the calculation of pairwise interaction energies between protein residues in equilibrium ensembles, usually generated from MD simulations. These energies are considered the ‘strength’ between the nodes (residues) and are mapped as edges onto the protein structure. Subsequently, the strength values are generally normalized in order to weight or deduce the information transfer rate from one node to another. Finally, these data are used in network analysis such as shortest path identification and node centrality calculations.

In this work, the interaction energies were calculated employing the pair-wise energy decomposition function of MMPBSA.py program [83]. For network construction, we followed the protocol proposed by Vijayabaskar *et*. *al* [95], where only van der Waals and electrostatic interactions of non-continuous residues were taken into account. Furthermore, all energies were normalized between zero and one, the more negative values being closer to one, and the more positive ones, closer to zero. Subsequently the betweenness centrality was calculated with the igraph package [101]. The normalized data were transformed into path length through the following formula: $w_{ij}\;=\;-log\left|{Inorm}_{ij}\right|$, where, *Inorm*_*ij*_ is the normalized interaction energy between residue *i* and *j* and *w*_*ij*_ is the path length. Hence, the information transfer rate will be greater for the more favorable interactions, as the latter will have shorter path lengths.

## Results

### Transient pockets and functional gate in a conserved groove of papain-like cysteine proteases revealed during MD simulations

**Fig 3:**
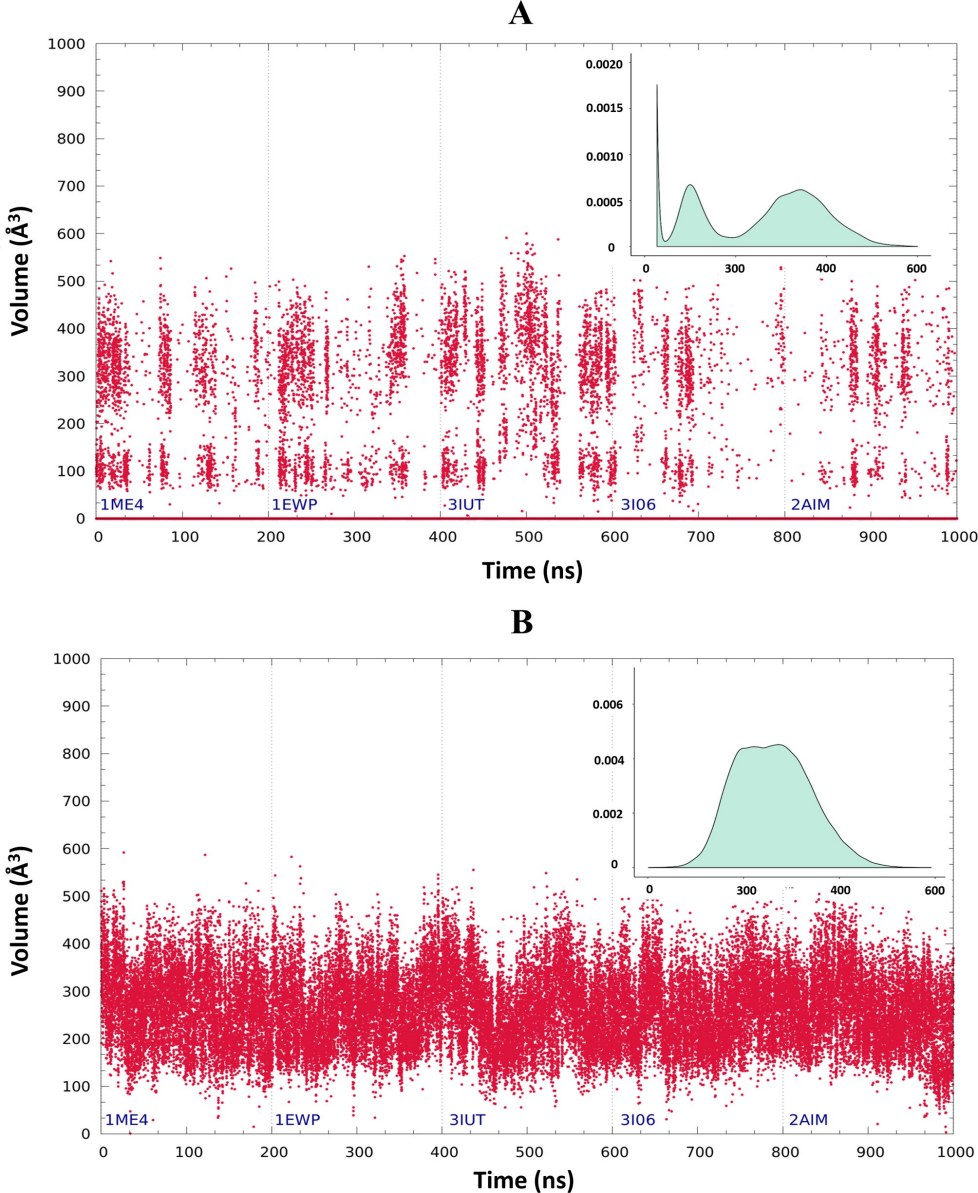
Dynamical analysis of cruzain sites 1 and 3. Time evolution of instantaneous volumes for site 1 (A) and site 3 (B). Graphs were subdivided according to the cruzain structure employed in each simulation (PDBID). Insets within each graph correspond to the volume probability histograms along the MD trajectory.

**Fig 4:**
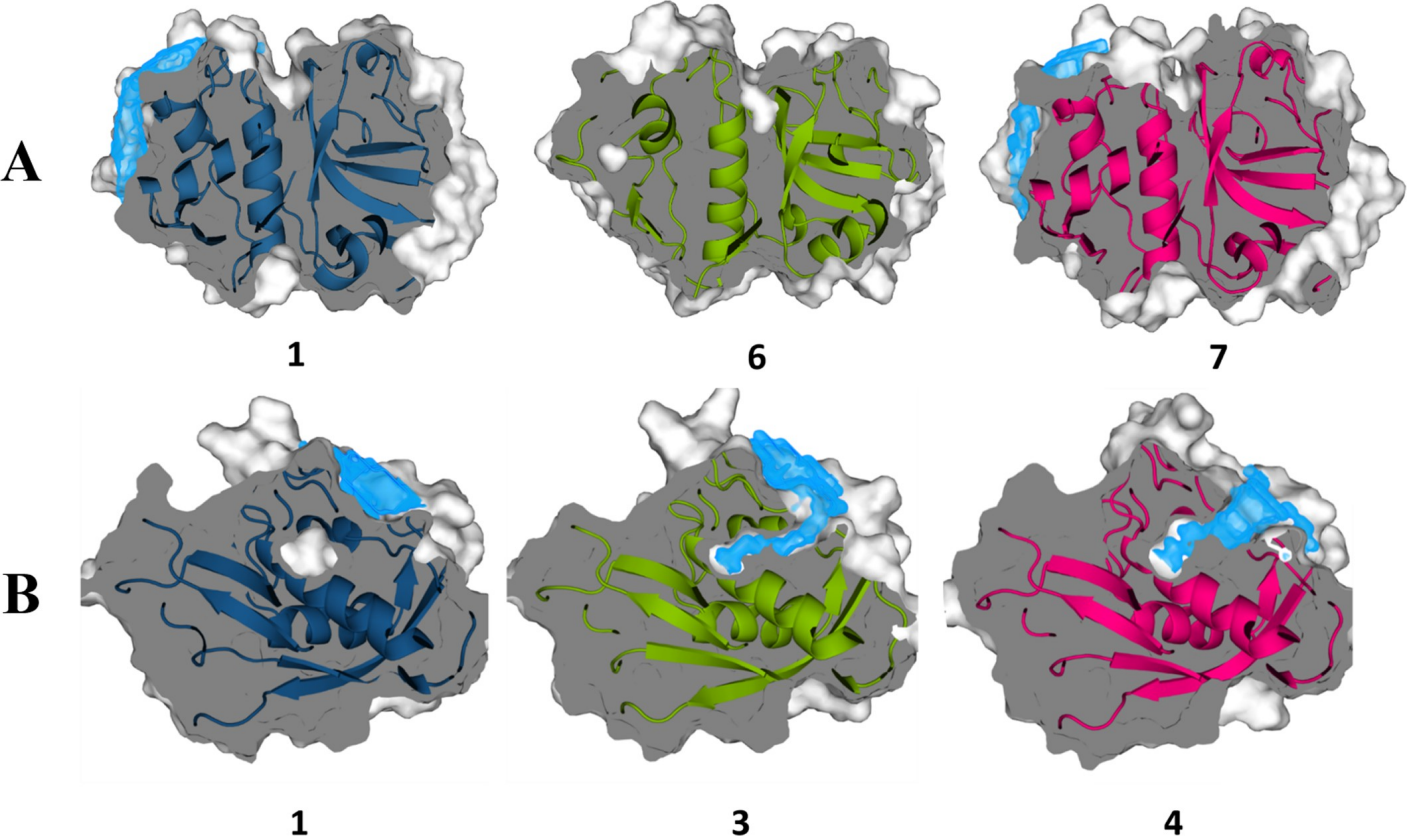
Representative structures of cruzain selected for ensemble VS against sites 1 and 3. Representative structures of (A) site 1 and (B) site 3. Volume densities of the three selected representative structures are depicted in blue. The corresponding cluster identifier is shown in each case.

After mapping the putative allosteric sites of HCatK onto the surface of cruzain through structural alignment (Fig 1), the characterization of cruzain cavities was performed through an analysis of the enzyme conformational dynamics. The selection of cruzain structures for MD simulation of the apo form is described in detail in S1 Text. The results showed that site 1 and site 3 display a suitable volume size (≥ 200 Å^3^) along simulation time (Fig 3); therefore, both were chosen for further VS experiments. Subsequently, three central structures per-site, satisfying the conditions described in Materials and Methods, were selected (Fig 4). The results of pairwise RMSD and volume comparison between all generated representative structures of site 1 and site 3 are shown in S3 Fig.

The time evolution of cavity volume indicated that site 3 is more stable than site 1 (Fig 3). However, the latter reached higher volumes, probably because it is flanked by a flexible fragment of cruzain loop(loop_84-109_), homologue of the occluding loop of human cathepsin B (S4A Fig), that eventually can unveil the larger pocket. Moreover, the distribution of conformational space of site 1 showed three well-defined populations, being the structures with zero volume the most representative ones (Figs 3A and 4A). The above results suggest that site 1 constitutes a cruzain transient pocket. On the other hand, cruzain site 3 showed a conformational space characterized by two overlapping normal distributions of volume values (Fig 3B). However, there was an intermediate representative structure (cluster 3) that shared structural characteristics with the remaining two (Fig 4B). Our predictions revealed the occurrence of an opening/occlusion dynamics of site 3 internal cavity (cluster 1 in Fig 4B), in which the salt bridge established between conserved residues LYS17 and GLU35 (see sequence alignment of [11]) acts as “functional gate” of this groove (S5 Fig).

Additionally, the RMSF analysis showed the same fluctuation pattern in the five replicas of the apo-form (S6B and S6C Fig). Here, we observed the main flexibility in cruzain loops, mainly in the region corresponding to loop_84-109_. These findings explained the huge volume variation of site 1, since several residues of the previous loop region are flanking this site (S4A Fig). Moreover, RMSD values calculated for heavy atoms showed different time evolution behavior between site 1 and site 3 (S6A Fig). In this sense, site 3 showed relatively stable RMSD value; whereas site 1 displayed structural fluctuations during simulation time, as expected for a site that displays volume instability.

### Preferential binding of identified hits to allosteric site 3 of cruzain

The prediction of allosteric modulators of cruzain was based on two rounds of high-throughput selection using: (i) Autodock Vina scoring function, and (ii) combination of MD simulations with MM-GBSA calculations as the final post-processing step. The comparison of the compounds identified through the previous strategy showed non-overlapping scaffolds targeting each of the three selected clusters of the same cavity. This arises from the large structural differences between the three representative structures selected for the ensemble VS (Fig 4), which reinforces the importance of using different receptor conformations in VS experiments to increase the structural diversity of the selected compounds.

**Fig 5:**
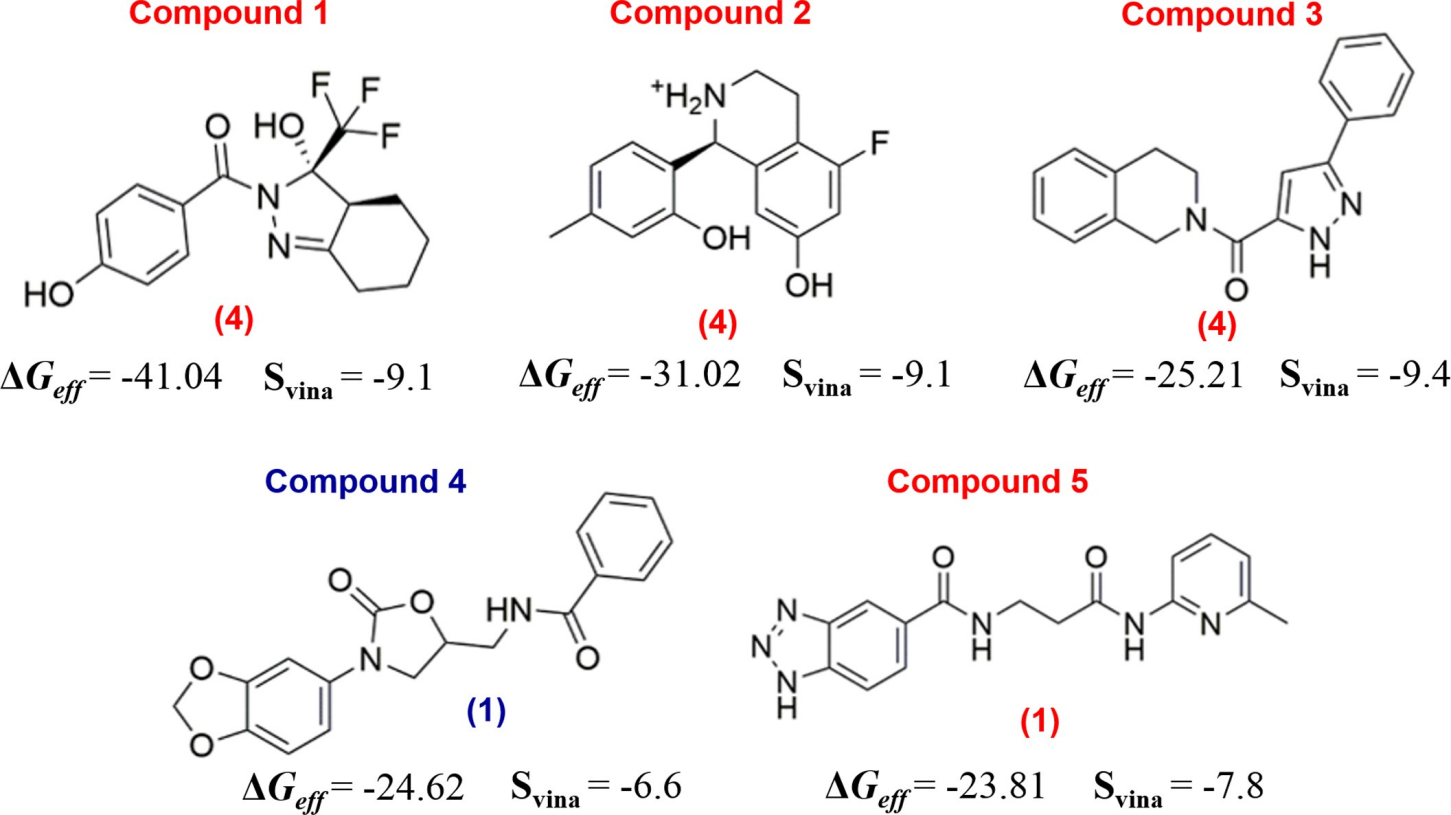
Chemical structures at pH = 5.5 of top five hits selected from the combination of VS and MM-GBSA calculations. Compound names are colored differently, according to the allosteric sites wherein they were docked, i.e., red for site 3 and blue for site 1. Below each chemical structure, the numerical IDs of cluster central structures that bind each ligand are depicted between parentheses. The S_vina_ and Δ*G*_*eff*_ values are expressed in kcal/mol units.

In Fig 5, the top five compounds are listed and numbered in increasing order according to the MM-GBSA free energy values. These results showed that the Δ*G*_*eff*_ values of the compound list ranged from -41 to -23 kcal/mol (Fig 5). Interestingly, ligands having the lowest energy values among all sets were detected in VS against the representative structures that possessed the largest volumes (Figs 4 and 5); thus underlining the importance of exploring the protein conformational space to identify different pocket structures. In our case, despite having many cruzain crystal structures (25), it was necessary to perform MD simulations to sample appreciable conformational changes prior to conduct VS simulations.

Finally, we selected three top-scoring compounds (compounds 1, 2 and 4, see Fig 5) in complex with cruzain in order to assess the time stability of these complexes in longer MD simulations, which, in turn, would suggest the suitability of the selected compounds and/or the druggability of the potential allosteric sites. Note that our selection was based on the overall best hits identified for the studied pocket, in terms of Δ*G*_*eff*_ values. We also considered that two compounds targeting site 3 were, in principle, a handful subset to investigate the potential allosteric modulation of cruzain arising from the ligand binding to this site, which requires the usage of computationally-demanding calculations. The ensemble generated from MD simulations are useful to analyze the potential allosteric effects triggered by the binding of the selected compounds to cruzain sites 1 and 3. Note that within the list of best hits (Fig 5), only compound 4 is derived from VS against site 1. This is a consequence of large solvent accessible area present in site 1 (S4A Fig), where the interactions of ligands with solvent molecules are likely to destabilize ligand-cavity interactions in short MD simulations (10 ns). Conversely, site 3 is a buried groove (S4B Fig), which can establish more stable interactions with ligands, but, again, at expense of large desolvation of the ligand.

**Fig 6:**
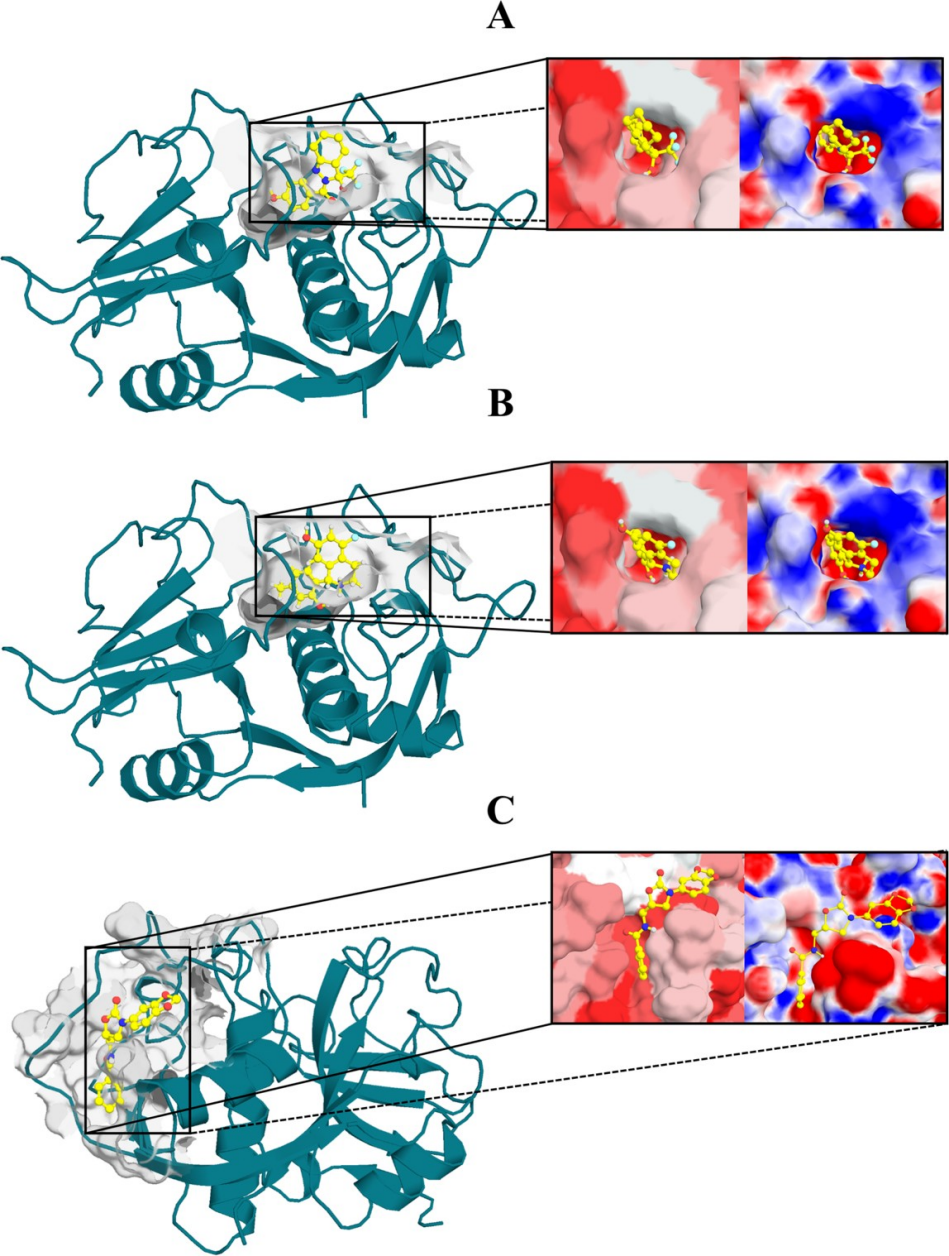
Selected docking poses for the best hits of each pocket. Top-scoring poses of (A) compound 1 and (B) 2 docked into site 3 pocket, and (C) top-scoring pose of compound 4 docked into site 1. Protein surface is colored according to hydrophobicity and electrostatic potential. The color scale for hydrophobicity ranges from red (hydrophobic) to white (hydrophilic), and for electrostatic potential, from red (electronegative potential) to blue (electropositive potential).

In Fig 6, the top-scoring docking poses of these ligands are represented. Moreover, each pocket surface was colored according to the electrostatic potential and hydrophobicity. These results show that site 1 is made up of residues with dissimilar electrostatic and hydrophobicity properties (Fig 6C). Hence, compound scaffolds that possess polar and non-polar interacting groups, such as compound 4, are suitable to target this site. On the other hand, site 3 has a positive region at the entrance, in contrast with its internal area, which is highly negative. This is due to the presence of LYS17 in a more solvent-exposed region of site 3 and GLU35, GLU50 and GLU86 positioned in the pocket inner region. The two top-scoring ligands selected for this site (compounds 1 and 2) contain aromatic rings placed within the pocket hydrophobic region (Fig 6A and 6B). In addition, the polar substituents of these ligands may interact with polar regions by forming hydrogen bonds and/or salt bridges, as in the case of compound 2 (positively charged, see Fig 5).

### Selected hits display stable interactions with site 3 during the long MD simulations

A comparative analysis of conformational dynamics and energetic profiles was performed, including the apo form, cruzain with a peptidic substrate, and the former systems bound to the allosteric ligands. The stability and convergence of long MD simulations (200 ns per replica) performed for cruzain complexes were monitored through per-frame Δ*G*_*eff*_ and ligand RMSDs (S7–S12 Figs). The accumulated average values of Δ*G*_*eff*_ became stable in most cases (S7–S11 Figs). The equilibration time of complexes was selected according to the behavior of Δ*G*_*eff*_ values across the simulation replicas. Accordingly, 60 ns was chosen as the start point for the rest of MD simulations analysis (S7–S11 Figs). On the other hand, in the MD simulation replicas of the cruzain-compound 4 system, the ligand dissociation was observed after 50 ns of simulation time. The latter points out the lack of a stability of the ligand in this site.

**Fig 7:**
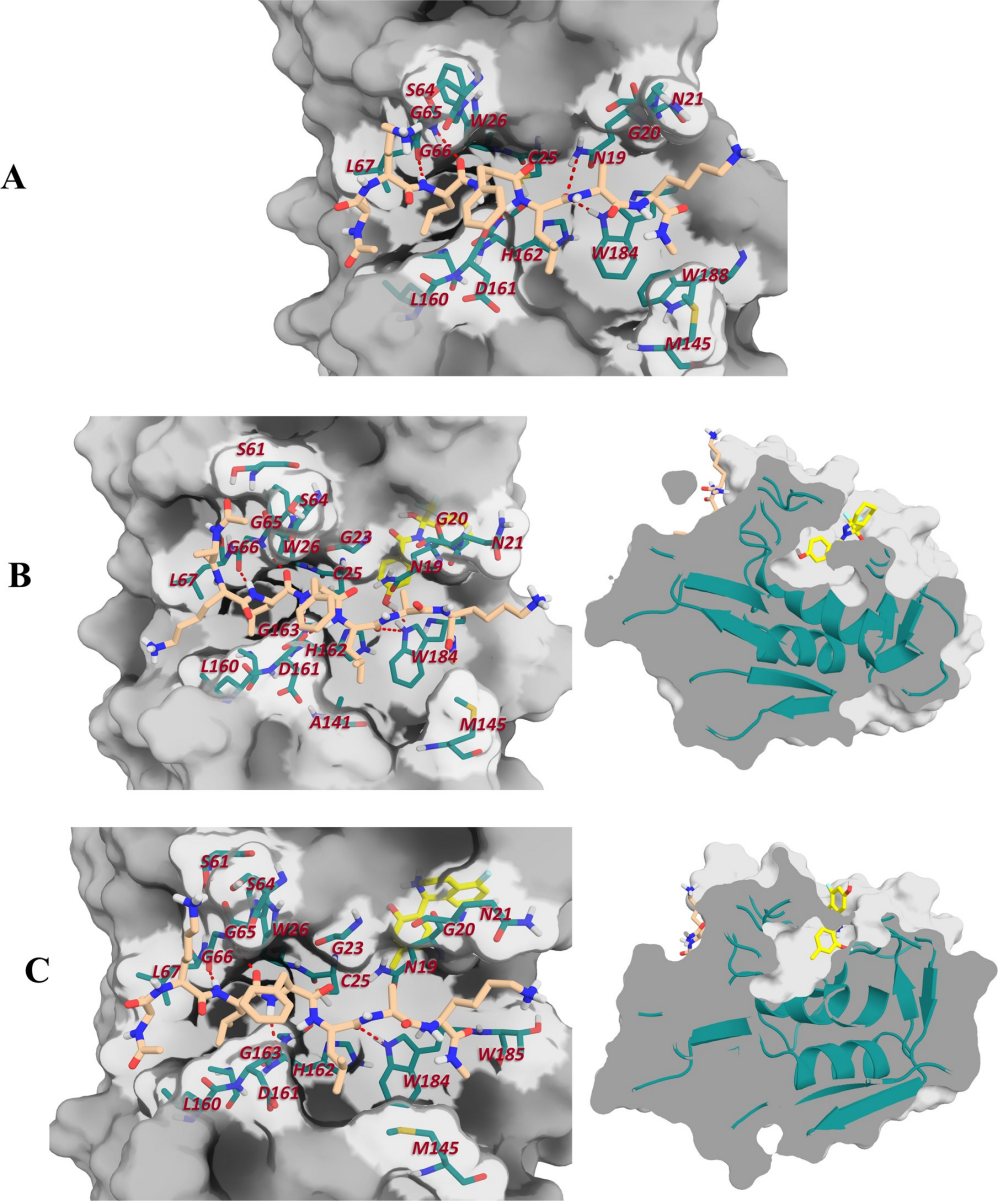
Main interactions established between substrate and cruzain binding site. (A) cruzain-substrate (B) cruzain-peptide-compound 1 and (C) cruzain-peptide-compound 2. The interacting residues are labeled in each case and the relative position of ligand (yellow) with respect to substrate (lightorange) is represented in the right panel.

Finally, the Δ*G*_*eff*_ values of ligand binding (Δ*G*_*eff*(ligs)_) were calculated as the average of five replicas in order to stablish the best hits (Table 1). The results show subtle differences between both compounds (~1 kcal/mol); therefore, both ligands may have the same priority in terms of enthalpic contribution for further experimental assays. In addition, moderate decrease in substrate affinity was observed when we compared the Δ*G*_*eff*(peptide)_ values in the presence of ligands (${\Delta \Delta G}_{pep,compd1}^{pep}$ = 2.86±1.39 and ${\Delta \Delta G}_{pep,compd2}^{pep}$ = 2.64±1.39, see Table 1). These lines of evidence indicate a moderate destabilizing effect on the formation of enzyme-substrate complex (Fig 7). Moreover, the hydrogen bond analysis of cruzain-substrate complexes showed that the most distinctive differences caused by the ligand binding to the allosteric site 3 were mainly in those established at the S1’-P1’and S2’-P2’ interfaces (Fig 7 and S1 Table). Note that these hydrogen bonds were also the most unstable in MD simulations corresponding to cruzain-substrate system (S1 Table). Interestingly, site 3 is close to the S1’-S2’ subsites (Fig 7), and the presence of ligands in that cavity directly affects the movement of loop_10-25_ and loop_182-192_, and, thus, the dynamic of aforementioned subsites. As was previously discussed, GLN19 and TRP184 are conserved residues placed in the former loops [11], and it has also been reported that they are essential for the catalytic mechanism in papain-like cysteine proteases [102]. Although moderate variations in Δ*G*_*eff*_ values for substrate binding were observed, the variations in the motions of these protein regions may affect the cruzain catalytic rate constant as well.

**Table 1:** Effective free energies calculated for intermolecular interaction of each system.^a^

System	cruzain-compound 1	cruzain-compound 2	cruzain-peptide	cruzain-peptide-compound 1		cruzain-peptide-compound 2	
**Replicas**	-28.83±0.07^b^	-30.55±0.1	-64.59±0.05	-27.73±0.04^c^	-65.26±0.06^d^	-30.73±0.05	-63.37±0.05
	-31.11±0.11	-32.5±0.08	-65.29±0.05	-26.71±0.03	-61.45±0.05	-31.84±0.04	-58.64±0.05
	-27.05±0.1	-24.72±0.13	-63.84±0.05	-28.53±0.04	-59.16±0.05	-31.54±0.04	-65.03±0.05
	-26.52±0.09	-28.78±0.09	-64.08±0.05	-27.96±0.04	-63.46±0.06	-28.77±0.04	-64.83±0.05
	-29.49±0.12	-30.15±0.11	-63.58±0.05	-28.30±0.04	-56.79±0.05	-31.64±0.04	-58.25±0.05
			-64.67±0.05	-29.35±0.03	-62.78±0.05	-30.3±2.98	-60.05±0.09
**Average**	-28,6±0.83	-29,34±1.3	-64.34±0.28	-28.1±0.29	-61.48±1.37	-30.8±0.52	-61.7±1.36

^a^ All energies are in kcal/mol

^b^ Standard Errors of mean

^c^ Energy calculated for ligand

^d^ Energy calculated for peptide

### Aliphatic chains of site 3 display large energy contribution to ligand binding

**Fig 8:**
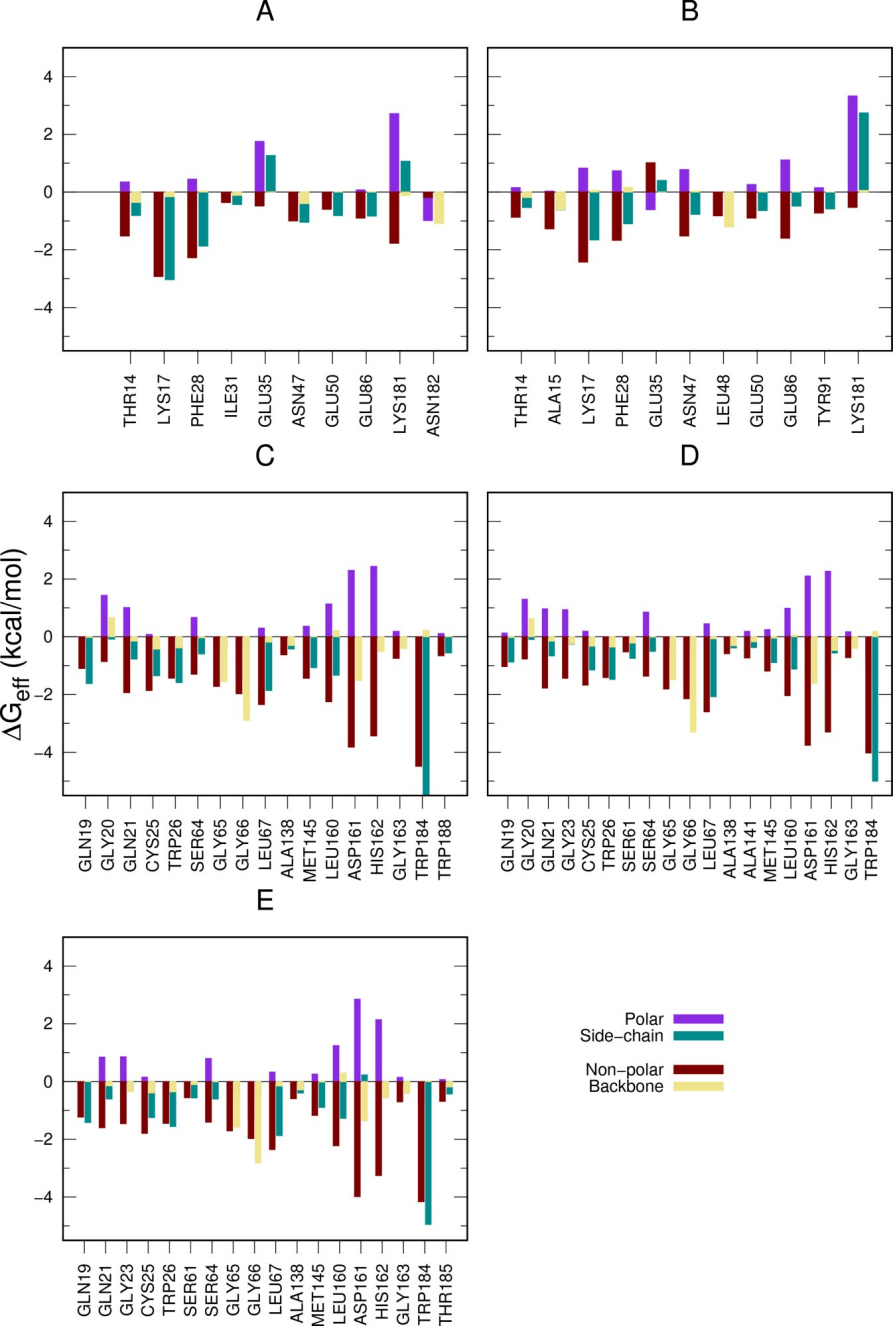
Per-residue free energy decomposition of cruzain complexes. The side chain, backbone, polar and non-polar contributions of each residue are displayed for all the analyzed complexes, i. e, (A) cruzain-compound 1 and (B) cruzain-compound 2, (C) cruzain-peptide, (D) cruzain-peptide-compound 1 and (E) cruzain-peptide-compound 2.

In order to get insights into the ligand/peptide interactions with cruzain, the per-residue decomposition of Δ*G*_*eff*_ *(*Δ*G*_*res*_*)* was employed. As it is shown in Fig 8, the non-polar interactions displayed the largest energy contribution to the binding process between cruzain and the substrate. Residues GLN19, CYS25, TRP26, LEU67, MET145, LEU160 and TRP184 were predicted as the main hot-spots of cruzain active site. Interestingly, there is a noticeable decrease in 1 kcal/mol of Δ*G*_*res*(TRP184)_ when ligands are bound to the alloteric site 3 (Fig 8C, 8D and 8E). This change partially accounts for the ΔΔ*G*_*eff*(peptide)_ observed between cruzain-peptide and cruzain-peptide-ligand systems (Table 1). Additionally, Δ*G*_*res*(GLN19)_ decreased in 1 kcal/mol when compound 1 is positioned within site 3. These variations reinforce our previous conclusion that the binding to site 3 affects the dynamics of S1’-S3’ subsites.

In general, we observed that despite site 3 surface being highly charged, van der Waals interactions are predominant in cruzain-ligand interfaces (Fig 8A and 8B). This is because the polar desolvation energy is more unfavorable in residues that establish polar interactions, i. e., GLU35, GLU86 and LYS181. The major hot spot residue at the interfaces of both cruzain compound complexes is LYS17, which interacts with the ligands through its aliphatic side chain, exposing its charged amine group to the solvent. Also, THR14, PHE28, ASN47 and GLU50 were predicted as critical interacting residues in studied cruzain-ligand systems.

**Fig 9:**
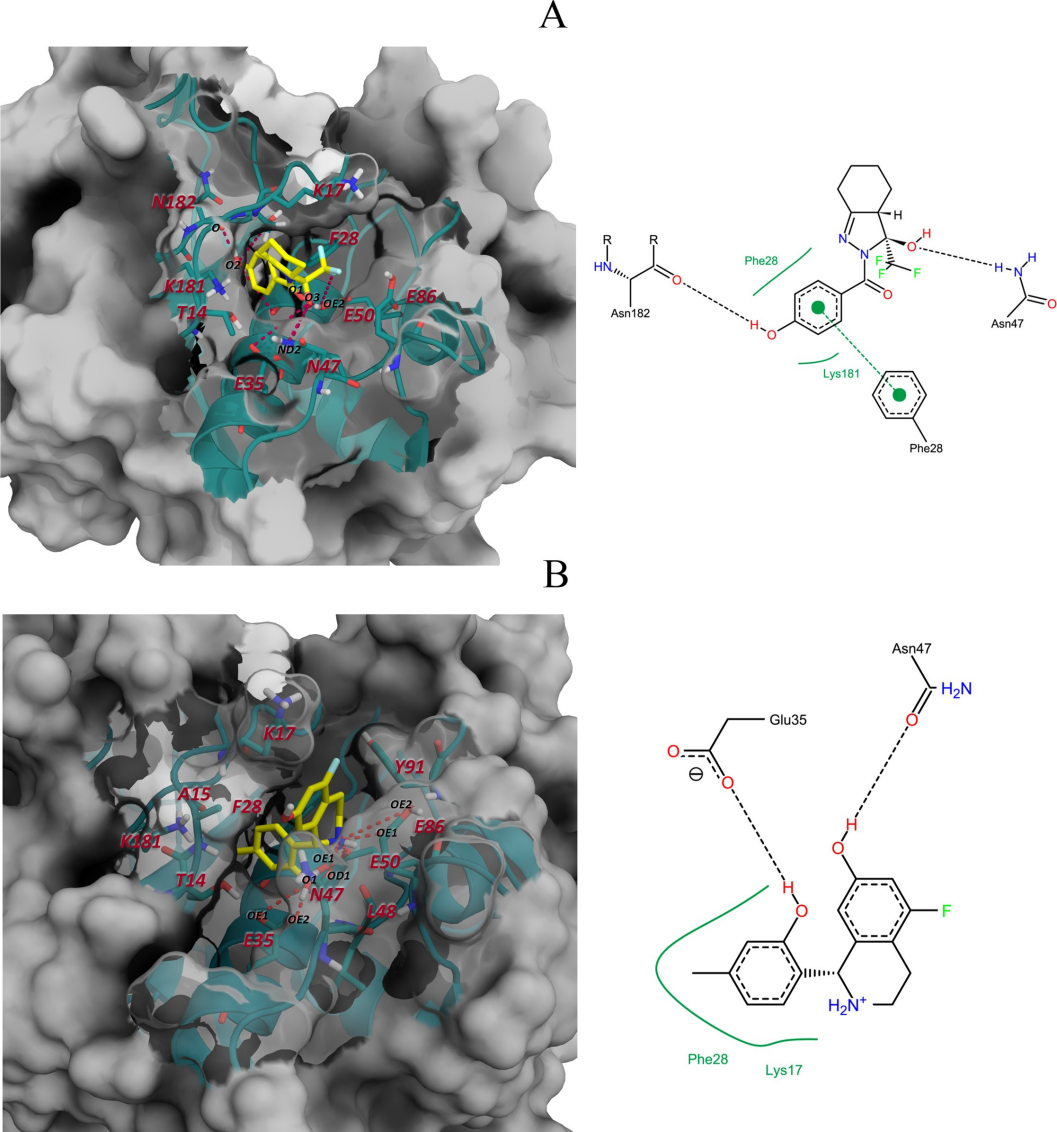
Structural representation of cruzain-ligand interfaces. 3D and 2D interfaces of (A) cruzain-compound 1 and (B) cruzain-compound 2. In each diagram, the residues considered energetically relevant are labeled together with the name of atoms forming the main inter-molecular hydrogen bonds (red dashed lines in left panel).

The further decomposition of Δ*G*_*res*_ into side chain and backbone contributions led to the identification of energetically-relevant positions within site 3. For almost every residue represented in Fig 8A and 8B, the energy contribution of side chain is larger than that of the backbone, except for ALA15, LEU48 and ASN182. This result suggests the essential role of positions of residues conforming site 3 in the interaction with ligands. Remarkably, three energetically-relevant residues, i. e., THR14, LYS17 and PHE28, are conserved positions within papain-like cysteine proteases [11], and were also characterized through SCA method as residues belonging to functional sectors. Therefore, they are likely to participate in allosteric regulation of HCatK [36]. On the other hand, GLU50 is a semi-conserved position [11], also lying within the same sector of the former residues [36], that is able to form hydrogen bonds with both ligands (Fig 9 and S1 Table). In addition, ASN47 is a non-conserved residue that establishes hydrogen bonds with both ligands. Thus, it may be crucial to the selectivity of cruzain allosteric inhibitors targeting site 3 (Fig 9 and S1 Table). Finally, note the unfavorable overall energetic contribution of conserved GLU35 in both complexes, where the polar desolvation energy is more unfavorable than its hydrogen bond energetic contribution.

In the cruzain-compound 1 complex, ASN182 establishes the most stable hydrogen bond through its backbone carbonylic oxygen (Fig 9 and S1 Table). Interestingly, ASN182 is a highly conserved residue whose participation in the catalytic mechanism of cruzain has been proposed [102]. In the cruzain-compound 2 complex, GLU86 also forms a hydrogen bond through its carboxylic oxygen atoms, but its polar desolvation energy is more unfavorable because of the presence of positive charge on the compound N atom. In addition, compound 2 establishes important van der Waals contacts with THR14, ALA15, PHE28, GLU86, GLU50, LEU48 and TYR91. However, LYS181 displays a more negative contribution to cruzain-compound 2 binding process because of the high desolvation penalty of the amine group, which is facing the phenolic ring of this ligand (Fig 9B).

### Compound 2 increases the flexibility of cruzain structure

**Fig 10:**
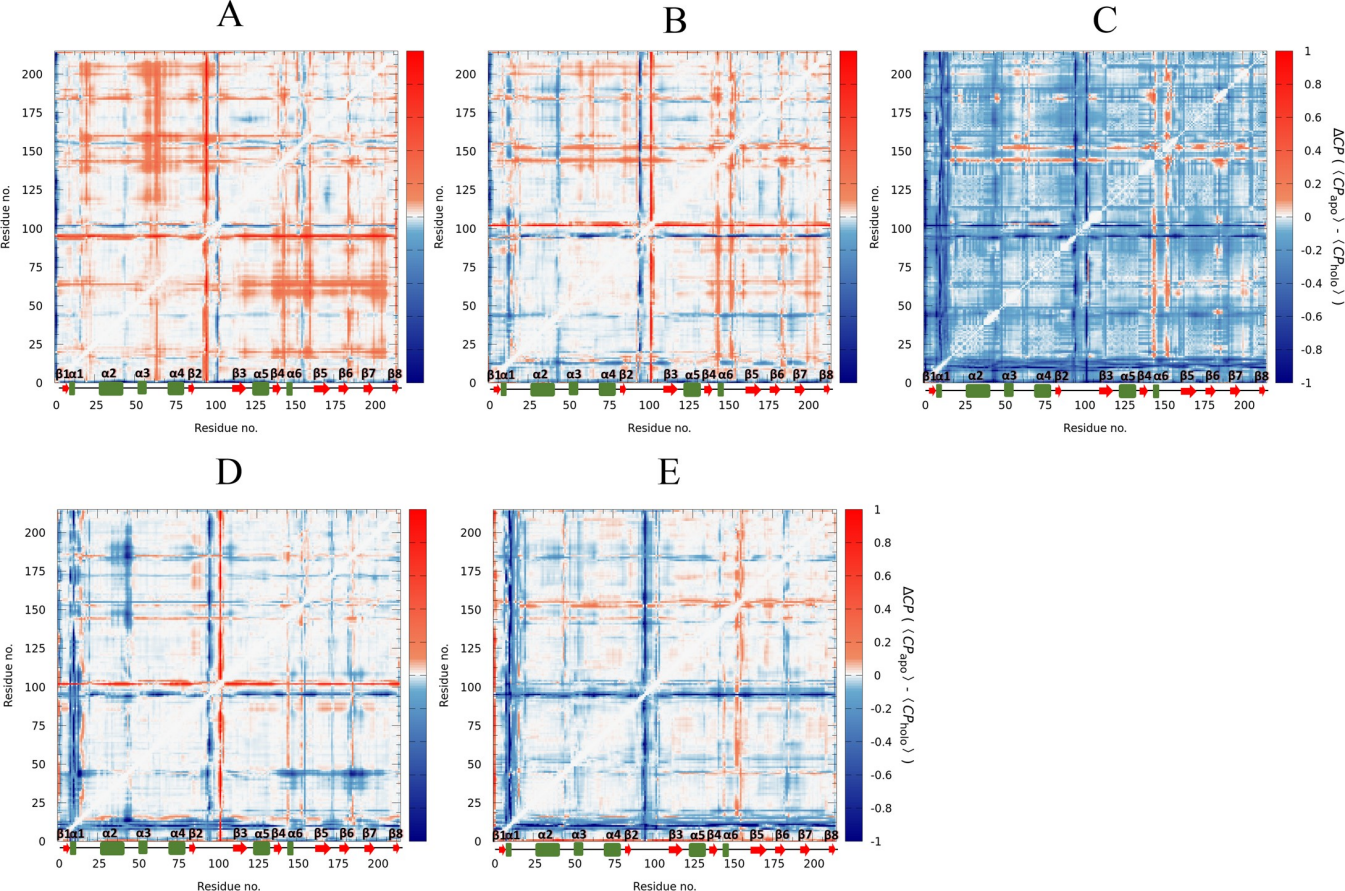
Analysis of motions of the apo and holo forms of cruzain. Graphic representation of matrices corresponding to *CP* differences (Δ*CP*). The *CP* values corresponding to (A) cruzain-peptide, (B) cruzain-compound 1 and (C) cruzain-compound 2 were subtracted from those of the apo form, and the *CP* values of (D) cruzain-peptide-compound 1 and (E) cruzain-peptide-compound 2 were subtracted from those corresponding to the cruzain-peptide system. The matrix scale is colored from blue (higher flexibility in holo form) to red (higher flexibility in apo form). The coloring scale was set between 1 and -1 in order to improve the visualization of Δ*CP* values.

Subsequently, we turned the attention to the characterization of the effects on cruzain internal dynamics triggered by ligand binding to site 3. The *CP* was calculated for each system in order to characterize the global rigidity/flexibility patterns. Here, we intended to seek for changes produced by ligand binding in the coordination of amino acids belonging to different protein domains. The matrices obtained from the *CP* differences between apo and holo forms are shown in Fig 10. Positive values in the matrices (red color) indicate higher flexibility in the apo form, while negative ones (blue color) denote higher flexibility in the holo form.

The results show that cruzain structure is less flexible when bound to the peptide than it is in the apo form (Fig 10A). Interestingly, the phenomenon of protein structure stabilization by substrate binding is commonly described in biological systems [103]. In addition, the more flexible regions of cruzain were those corresponding to loops, which is in accordance with RMSF results (S6 Fig). On the other hand, the large white region of matrix corresponding to cruzain-compound 1 complex indicates similar patterns in flexibility/rigidity between apo and holo forms (Fig 10B). The main differences are also located in loop regions, while the same degree of internal coordination is maintained in residues belonging to secondary structures. In these systems, the greatest Δ*CP* values occurred at loop_84-109_ and loop_139-161_, where differential patterns in residues flexibility in both apo and holo forms were observed (Fig 10B). Conversely, the MD simulations of cruzain-compound 2 complex indicated a uniform increase in overall protein flexibility, favoring distortions in both, loops and well-defined secondary structures (Fig 10C).

Interestingly, the results of pairwise distance distributions of key residues of cruzain active site show, in most cases, that their respective maxima shifted towards lower values when compound 2 is bound to cruzain (S2 Text and S13 Fig). This suggested that the S3 and S2 subsites are slightly narrower when the former ligand is bound to site 3. Therefore, the substrate binding site is likely to undergo a small structural reorganization. Noteworthy, compound 2 is positively charged at the pH employed for MD simulations (pH = 5.5, see Fig 5), which could induce a perturbation on protein movements and electrostatic balance. However, we observed no significant changes on protein surface electrostatic potential subsequent to this ligand binding (S15 Fig).

A parallel analysis of matrices corresponding to cruzain-peptide-ligand systems displayed low changes in *CP* values due to the ligand presence in site 3. It is worth noting the increase of flexibility in helix 1 (7–10 residues) and regions corresponding to positions 40–45 and 90–95. Interestingly, we observed a noticeable less perturbation in protein flexibility when we compared these systems with those lacking the peptide in the active site, notwithstanding the ligand binding to site 3.

**Fig 11:**
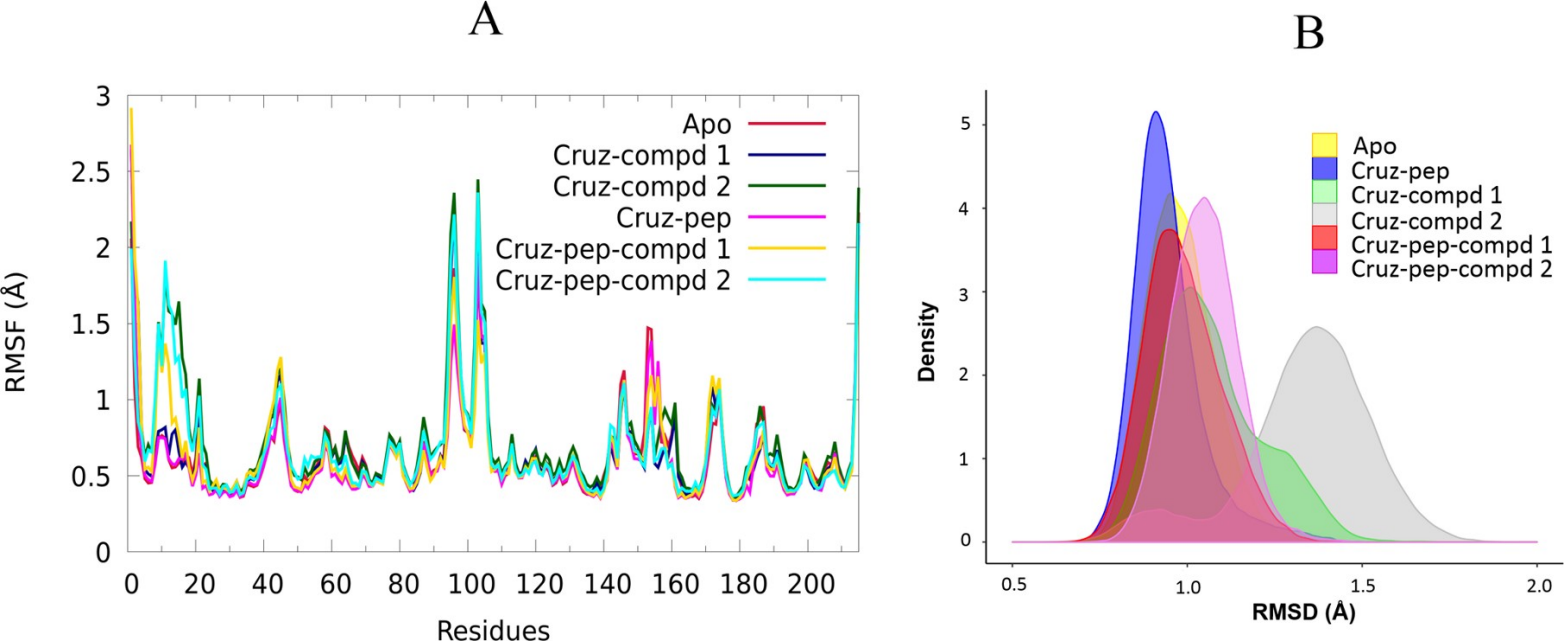
RMSF and RMSD values of cruzain analyzed complexes. (A) RMSF profiles and (B) RMSD distribution calculated for analyzed systems of cruzain. Each parameter was calculated for backbone atoms and taking as reference structure the starting frame of productive MD simulations.

Other significant changes in structure dynamics of cruzain were detected in RMSD and RMSF analyses (Fig 11). The RMSF results detected once again the cruzain loops as the most flexible regions of this enzyme (Fig 11A), and these predictions were also confirmed by the essential dynamics analysis (S3 Text). Note, that loop_10-25_ increases its flexibility in presence of ligands, and some residues belonging to this loop were also defined as hot spots in the section of per-residue energy decomposition of cruzain-ligands complexes. The RMSD distributions of the analyzed systems showed partially overlapping populations between them (Fig 11B). Here, in agreement with previous results, we could observe that the substrate binding induced small dynamic changes in cruzain structure, leading to an increased stability of peptide-bound complexes, which had the narrowest range of RMSD values. Conversely, a notable shift of the RMSD distribution toward higher values was detected when cruzain is in complex with both ligands, indicating an increase of enzyme conformational motions. Interestingly, the highest RMSD values were observed for cruzain-compound 2 complex (an increase of ~1.0 Å, see Fig 11B), which agreed with previously-discussed results of *CP* analysis (Fig 10C). Therefore, the dynamic changes induced by compound 1 were smaller than the respective variations of cruzain complexed with compound 2.

### Correlation network analysis reveals state-specific differences in residue couplings

**Fig 12:**
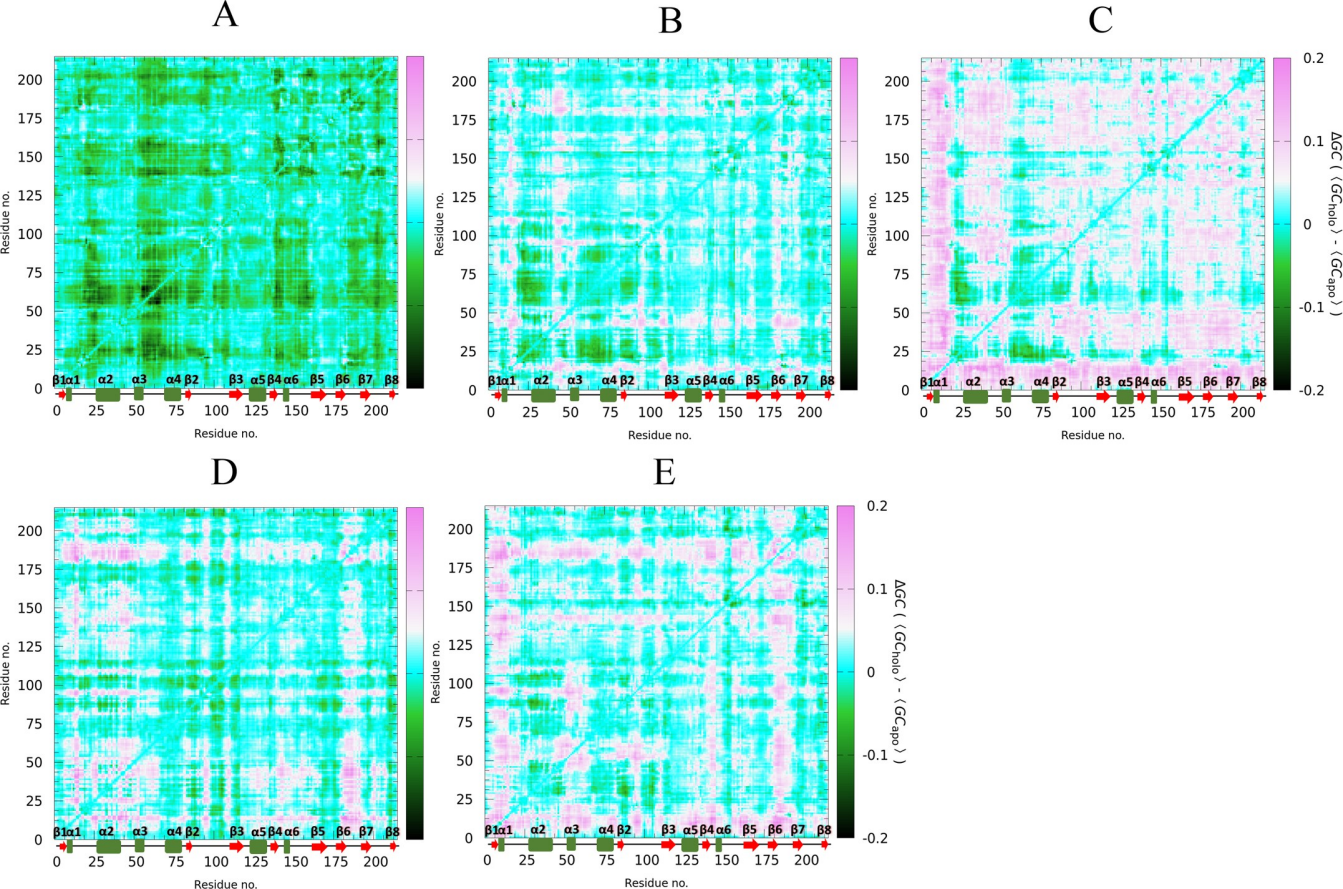
Comparison of generalized correlation coefficients of the analyzed cruzain systems. Δ*GC* matrices are represented as the subtraction of the apo form values from those corresponding to (A) cruzain-peptide, (B) cruzain-compound 1 and (C) cruzain-compound 2 systems, and as the subtraction of the cruzain-peptide from those calculated for (D) cruzain-peptide-compound 1 and (E) cruzain-peptide-compound 2 complexes.

The analysis of residue-residue couplings derived from MD simulations showed remarkable differences between apo and cruzain-peptide systems (Figs 12A and S18). Remarkably, the correlation decreases in regions corresponding to protein loops in cruzain-peptide system. The latter agrees with previous results that revealed the decrease in cruzain motions when the substrate lies within the enzyme active site. In parallel, the results obtained for apo and holo enzyme revealed an increase in correlations, triggered by ligand binding, mostly in cruzain-compound 2 complex (Fig 12C). This is demonstrated by the prevalence of positive values in the Δ*GC* matrix, i.e., holo minus apo correlations (pink spots). In the case of cruzain-compound 1 complex, an appreciable increment in correlation values was observed for residues lying in positions 14–19, 45–50, 90–100 and 180–190 (Fig 12B); while in the cruzain-compound 2 complex, many regions of the protein increased their coupled motions (Fig 12C). Besides, a decrease in correlation values (green points) of helix 2 (CYS25 localization) is observed in both systems (Fig 12B and 12C). Changes in coupled motions involving to interface residues of holo form are associated with the ligand-mediated “linkage” between loop_10-25_ and loop_84-109_. In addition, the comparison of correlated motions between cruzain-peptide and cruzain-peptide-ligand systems, revealed an intensification of couplings in regions comprising positions 10–55 and 182–192 (loop between β6 and β7) when compound 1 is bound to site 3 (Fig 12D). Likewise, when compound 2 is present, the correlated values increased in regions corresponding to N-terminal-20, α3, loop between β2 and β3, and loop between β4 and α6 (Fig 12E). It is important to highlight that in both cases, several residues interacting with aforementioned ligands lie in structural regions, which augmented their coupling motions.

**Fig 13:**
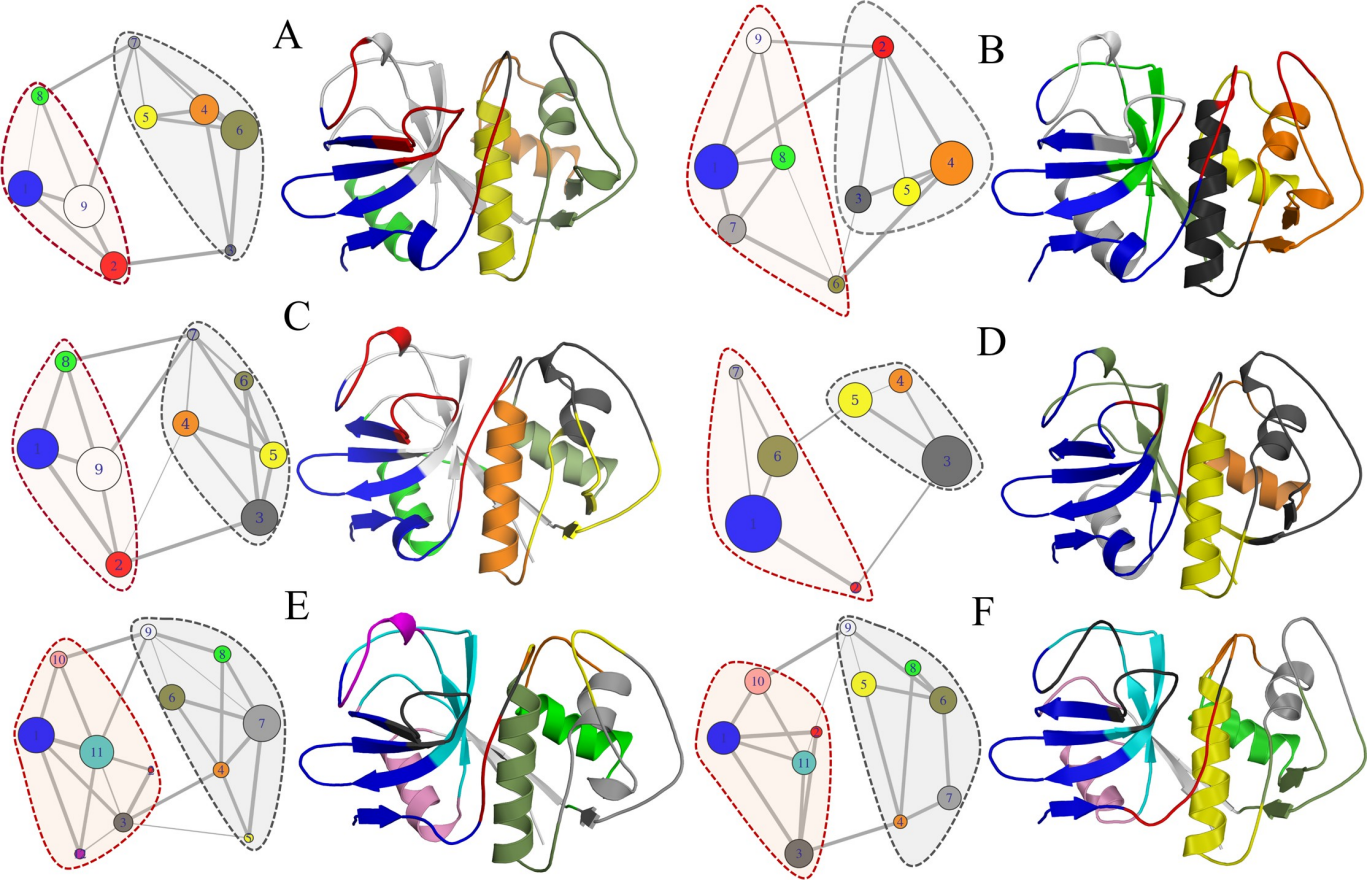
Community network analysis. Community structure corresponding to (A) cruzain apo form, (B) cruzain-peptide, (C) cruzain-compound 1, (D) cruzain-compound 2, (E) cruzain-peptide-compound 1 and (F) cruzain-peptide-compound 2. Dashed lines enclose the cruzain structural domains and edge thickness is proportional to the number of shortest path passing through those junctions. The circle diameter is proportional to the community size (i. e., number of residues).

The correlation network analysis further dissects residue couplings and reveals residue communities having the potential to facilitate long-range allosteric signal propagation. Also, the modification in the total number of communities and in the rearrangement of their residue composition provides a description of dynamical changes induced by effector binding during simulation time. In this sense, the study of intercommunity coupling strength in cruzain revealed the state-specific coupling paths and overall dynamical modules within the enzyme. The structure of papain-like proteases family are clearly partitioned into two main domains, and the analysis of time-averaged weighted networks adequately captured both of them in all the studied systems (Fig 13). In the network-based representation of cruzain apo form, its catalytic residues are distributed in three different communities, i. e., CYS25 in community 5, HIS162 in community 9 and ASN182 in community 2; while the two domains are linked by communities 7–9, 7–8 and 2–3. This highlights the indirect coupling of the catalytic residues. The analysis of intercommunity coupling strengths reveals state-specific allosteric coupling between site 3 and the active site through the edges between communities 6 and 5, and communities 2 and 9 (Fig 13A). On the other hand, the peptide binding induced changes in both domains and residue rearrangement within communities. Note that β-sheets-rich domain (delimited by red dashed lines in Fig 13) increased its community number; while the α-helix-rich domain (delimited by gray dashed lines in Fig 13) reduced the number of communities by lumping some of them together (Fig 13B). More importantly, the peptide binding induced a significant reorganization within the communities forming the cruzain active site (communities 2, 3, and 8 in Fig 13B). In this system, the variation in correlated motions comprising loop_139-161_, loop_181-193_, β3 and β8 played the main role in the fragmentation of the community containing HIS162 (community 9 in Fig 13A, which was fragmented in communities 8 and 6 in Fig 13B). Moreover, in the α-helix rich domain, the communities reordering involved the “packing” of loop_10-25_ and loop_84-109_. It is also important to highlight the loss of a direct correlation edge between the community containing the HIS162 residue and those corresponding to the α-helix rich domain in the cruzain-peptide complex (Fig 13B).

The comparison of community structure between the apo and complexed systems revealed remarkable conformational changes induced by effector binding (Fig 13C and 13D, see also S2 Table), highlighting a rearrangement of community numbers and its residue members in holo systems. The cruzain-compound 1 complex displayed a change in community 3 size and a new weak coupling between the main domains appeared (edge between community 4–2 and 4–3). However, the strength of direct interactions remained unchanged with respect to the apo form, except for the interaction between communities 1 and 8, which enhanced their correlation. Note that community reordering took place in the α-helix domain, specifically in loop_41-50_ and loop_84-109_, which are located within site 3 and site 1, respectively (Fig 13C). Conversely, in the cruzain-compound 2 complex, a whole rearrangement in communities was observed in both protein domains (Fig 13D). Here, the domain containing the helix 2 lost two communities, inducing more concerted motions within its structure. On the other hand, the binding of compound 2 induced changes in β-sheets-rich domain, with reassignment of a few residues from communities 2, 8 and 9 in the newly formed community 1. The decrease in community numbers suggests that compound 2 triggers an overall structural reorganization of cruzain. The strength of inter- and intra-domain couplings increased considerably in this system, which could affect the overall enzyme activity. Indeed, the appearance of a new correlation between two domains through communities containing residues of active site was observed, indicating a perturbation in the correlations of these residues (edge between community 6 and 5 in Fig 13D).

The comparison between the community networks of cruzain-peptide-ligand systems with respect to the remaining complexes shows an increase in community number, which indicates a disruption in correlated motions because of ligand presence in site 3 (Fig 13E and 13F). Note that the α-helix rich domain was split into six communities in both ternary systems (compare Fig 13B with Fig 13E and 13F). In this case, the main changes were detected in correlations of residues lying in loop_84-109_ and loop_10-25_ (Fig 13E and 13F). Another key factor that drives the fragmentation of communities in these systems was the substrate presence, which also prevented the increase in correlated motions (compared Fig 13C and 13D with Fig 13E and 13F). Therefore, these findings indicate that there are less overall protein movement when both, the binding site and site 3 are occupied.

### Network path analysis reveals couplings between cruzain orthosteric site and allosteric site 3

**Fig 14:**
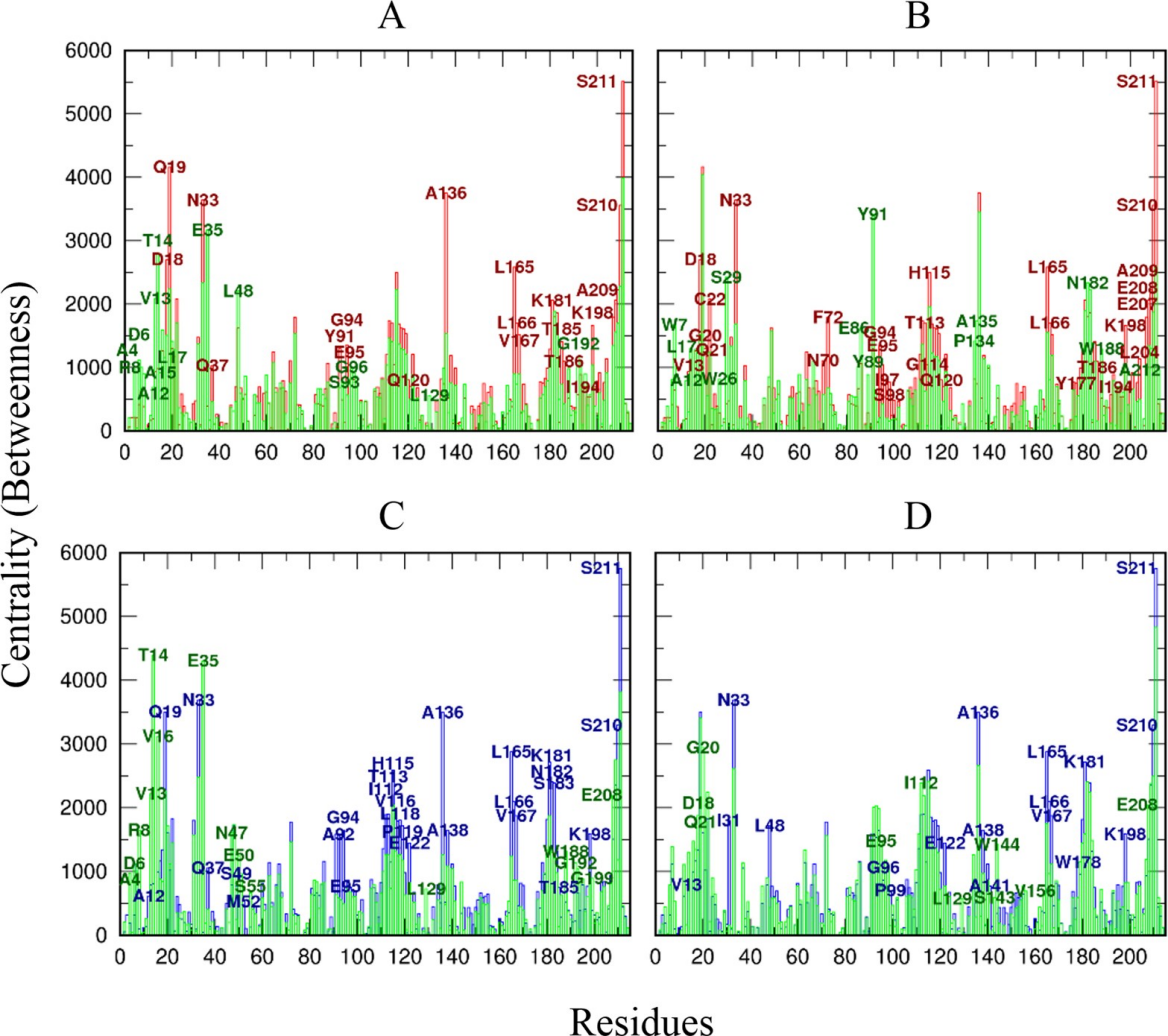
Residue centrality in cruzain analyzed systems. For convenience, the centrality profiles are represented as a comparison between ligand-bound and reference systems, i. e., cruzain apo form (red lines) and cruzain-peptide complex (blue lines), and cruzain-ligand complexes (green lines): (A) cruzain-compound 1, (B) cruzain-compound 2, (C) cruzain-peptide-compound 1 and (D) cruzain-peptide-compound 2. Residues with a ΔCentrality greater than 500 are labeled with the same color of their corresponding system.

The residue centrality based on average betweenness can characterize and differentiate highly connected residues that mediate stable interaction networks and allosteric communications in protein structures. This parameter can be presented as profiles, being a guiding indicator for the identification of functional residues critical to allosteric regulation. The centrality analysis highlighted remarkable differences in the way information is propagated through free cruzain versus the enzyme bound to allosteric modulators (Fig 14). These results showed changes in the centrality degree upon ligand association, indicating an alteration in dynamic couplings and a reduction in the diversity of the shortest pathways connecting all residues.

In the cruzain apo form, some residues with high-density network connections, i.e., ASP18, GLN19, ASN33, HIS115, ALA136, LEU165 and residues belonging to β8 were detected (Fig 14A and 14B). Note that most of these residues lie at the interface of the two domains of cruzain (i.e., GLN19, ASN33, and residues from β3, β4, β5 and β8), thus suggesting the important role of interdomain signal transduction in the protein function. It is noteworthy the high centrality values of GLN19, ALA136 and LEU165, which are key positions for catalysis and substrate recognition in papain-like proteases [102].

In the cruzain-compound 1 complex, new highly connected residues arouse, i. e., loop_10-25_, GLU35 and LEU48 (Fig 14A), many of which lie within site 3. These results point out that ligand presence increases the number of shortest paths crossing over site 3, making this groove a potential region for control of enzyme function. In the case of cruzain-compound 2 complex, residues LYS17, TRP26, SER29, GLU86, TYR91, VAL135, ASN182 and TRP188 increased their centrality (Fig 14B), being LYS17, GLU86, TYR91 and ASN182 part of site 3. Interestingly, most residues mentioned above are conserved across the papain-like cysteine proteases [11]. This suggests that ligands designed against site 3 may generate an allosteric signal involving common residues of this protease family, which could trigger the inhibition of the enzyme catalytic function.

Similarly to the apo form, in cruzain-peptide system, the residues positioned at the interface between the two domains of the enzyme showed the highest values of centrality, i.e., GLN19, ASN33 and β3, β5 and β8 (Fig 14C and 14D). In cruzain-peptide-compound 1 system, there was an increase in centrality values within the N-terminal region including loop_10-25_ (Fig 14C). However, there was no appreciable differences between cruzain-peptide-compound 2 complex and the reference system (Fig 14D).

On the other hand, optimal and suboptimal paths were calculated between CYS25 and each hot spot previously identified for site 3. Among all the pair of residues analyzed here, CYS25-THR14 was the one showing the largest increase in correlation between site 3 and the catalytic core because of ligand binding. This is consistence with centrality analysis where THR14 was one of the main residues displaying significant changes in its connections as a consequence of ligand binding (Fig 14).

**Fig 15:**
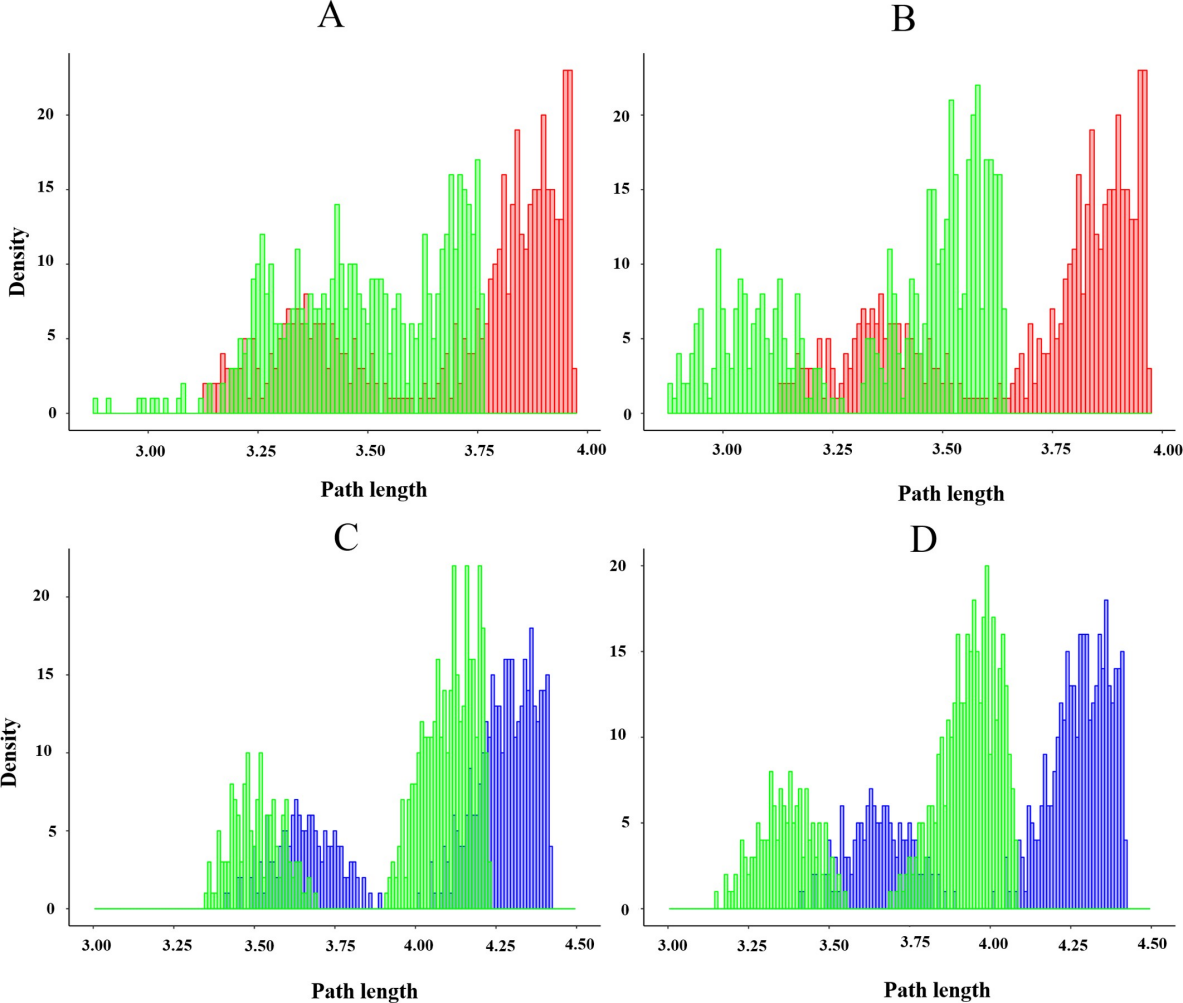
Statistical distribution of suboptimal paths. The histogram of the 500 path lengths associated with the apo and holo trajectories. Histograms corresponding to (A) cruzain-compound 1 and (B) cruzain-compound 2 were compared with apo distribution (red lines), while the histograms concerning to (C) cruzain-peptide-compound 1 and (D) cruzain-peptide-compound 2 complexes were compared with that of cruzain-peptide system (blue lines).

**Fig 16:**
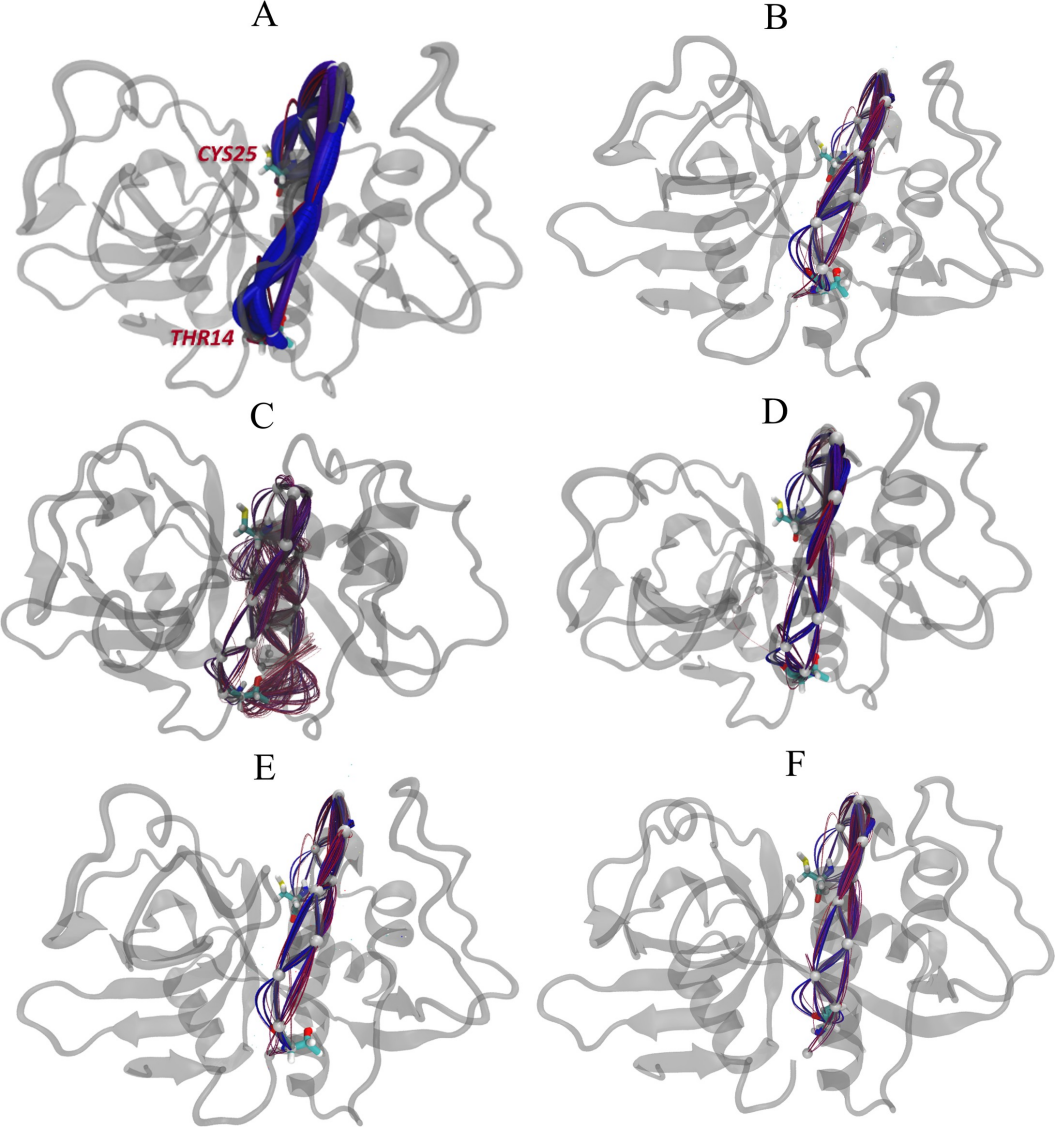
Suboptimal paths of cruzain analyzed systems. The 500 suboptimal paths between THR14 and CYS25 residues were calculated for (A) cruzain apo form, (B) cruzain-peptide, (C) cruzain-compound 1, (D) cruzain-compound 2, (E) cruzain-peptide-compound 1 and (F) cruzain-peptide-compound 2 trajectories. The shorter and longer paths are shown as blue and red splines, respectively.

By examining the path lengths distribution, we found that the shortest path of cruzain-compound 1 complex do not differ substantially from that of the apo enzyme (3.12 for apo from; 2.88 for cruzain-compound 1) (Fig 15A). Nevertheless, the distribution derived from this system trajectory is slightly deviated toward shorter path lengths, indicating that motions of the residues linking the allosteric and catalytic sites are more tightly correlated when compound 1 is bound. The determination of residues critical to the allosteric signal propagation (residue with highest degeneracy values), revealed the occurrence of different paths between the free enzyme and cruzain-compound 1 complex (Figs 16A, 16C and S19). The shortest path between THR14 and CYS25 in the apo state was: THR14 → VAL16 → ASP18 → GLN19 → CYS22 → SER24 → CYS25. In contrast, the optimal path when compound 1 is bound comprise the following residues: THR14 → GLU35 →ILE31 → PHE28 → CYS25. Here, a considerable decrease in node degeneracy values and the increment in the number of new residues involved in suboptimal paths are observed when compound 1 is bound (S19 Fig). This analysis identifies two main routes for allosteric communication, i.e., one that goes through helix 2 and the other one through loop_10-25_ (Fig 16A and 16C).

On the other hand, a shift of centrality values towards lower ranges was also observed for cruzain-peptide-compound 1 complex (3.41 for cruzain-peptide; 3.35 for cruzain-peptide-compound 1) (Fig 15C). In this case, the same shortest path was identified for cruzain-peptide-compound 1 and its reference system, which passed through loop_10-25_ (i. e., THR14 → VAL16 → LYS17 →ASP18 → GLY20 → CYS22 → SER24 → CYS25) (Fig 16B and 16E). Overall, we conclude that the binding of compound 1 alters the distribution of cruzain internal pathways.

The suboptimal paths between THR14 from site 3 and catalytic cysteine transverse almost the same nodes in cruzain-compound 2 complex and apo form (Fig 16A and 16D). Interestingly, the signal propagates directly to the catalytic core through the residues of loop_10-25_, being the optimal path for cruzain-compound 2 complex: THR14 → VAL16 → LYS17 → ASP18 → GLY20 → CYS22 → GLY23 → SER24 → CYS25. Even though the two systems share a similar optimal path, a strongest correlation between site 3 and the catalytic site in the holo form is detected. This becomes apparent by observing that the shortest path in cruzain-compound 2 system shifts towards lower length value (apo, 3.12; holo, 2.88) (Fig 15B). The previous shift is likely to arise from a more coherent signal propagation in the holo simulation, indicating a possible decrease in the entropy along the pathways due to compound 2 binding. In addition, an increase in the number of suboptimal paths with lower length values is observed in cruzain compound 2 complex with respect to the cruzain-compound 1 (Fig 15A and 15B).

Finally, the cruzain-peptide-compound 2 complex displayed an identical shortest path to that of the cruzain-peptide and cruzain-peptide-compound 1 systems (see explanation above). However, the distance of this path in terms of correlated motions was shorter than the other two (3.14) (Figs 15D and 16F). All these lines of evidence point out a dynamical tightening of the active site with site 3 in presence of compound 2, which might suggest, more potent allosteric influences of this compound in comparison to the other one.

### Energy interactions networks of cruzain reveal significant changes in interactions of ionizable residues of site 3 in presence of allosteric ligands

The structural stability and function of proteins depend on intricate network of inter-residue interactions. In this sense, the PENs approach accounts for the structural plasticity, stabilizing and hierarchical organization in protein structures. This analysis also provides a more realistic estimate of the communication paths within protein structure in energetic terms and clarifies the changes underlying in two existing forms of the same system (ex. bound and unbound, wild and mutated) [95].

**Fig 17:**
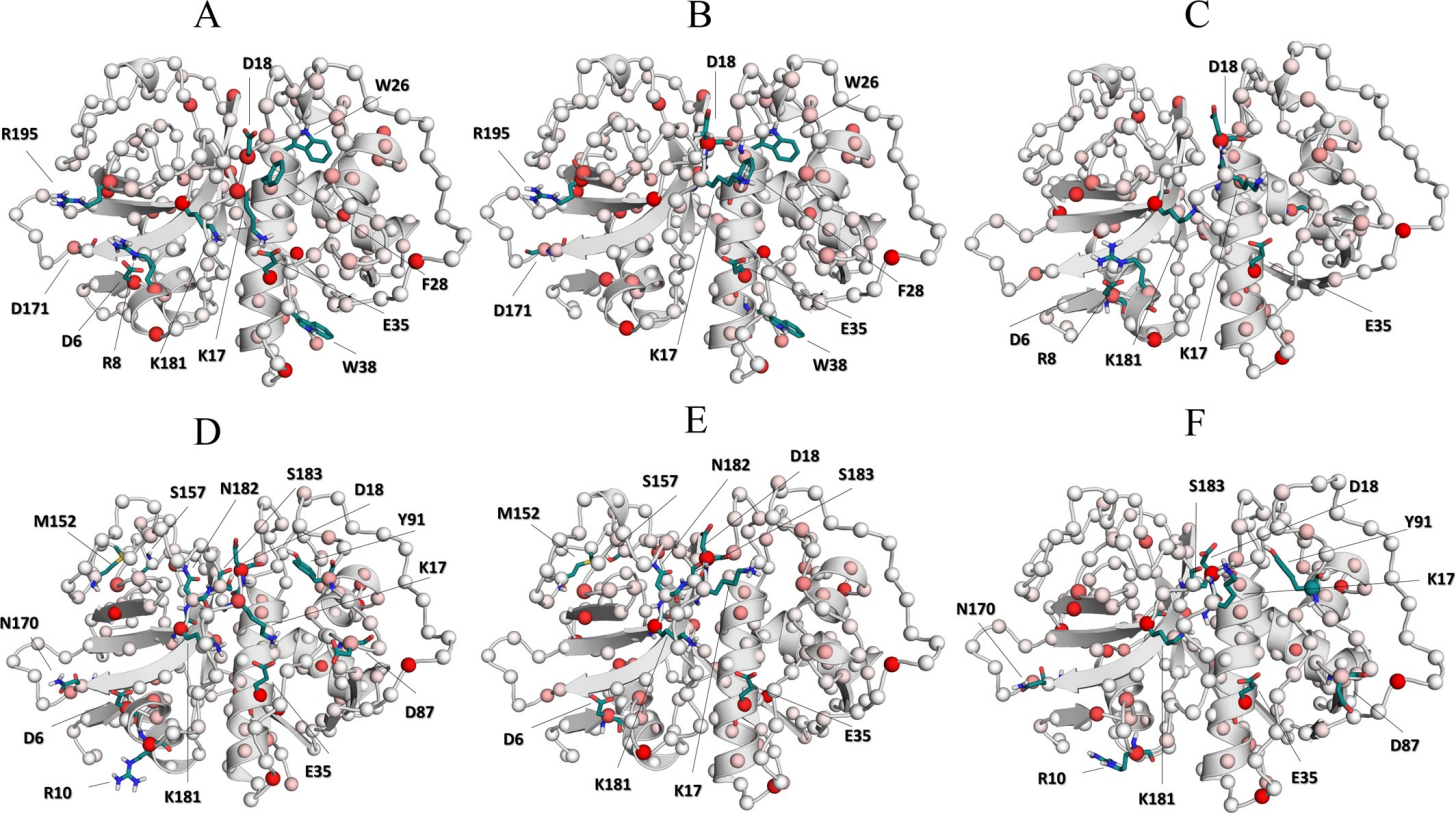
Comparison of betweenness centrality of each cruzain residue obtained from PENs. Representation of per-residue centrality calculated in each simulated system, i. e., (A) cruzain apo form, (B) cruzain-compound 1, (C) cruzain-compound 2, (D) cruzain-peptide, (E) cruzain-peptide-compound 1 and (F) cruzain-peptide-compound 2. Centrality values of the C-alpha atoms within cruzain structure are representend using a coloring scheme ranging from red (high centrality values) to white (low centrality values). Residues that changed the centrality upon ligand binding, are depicted as stick in reference and ligand-bound system, respectively.

In order to evaluate the effect of ligand binding on the residue interaction networks, we examined the residue centrality profiles of cruzain ensembles in their apo/substrate-bound and inhibitor-bound forms. As shown in Fig 17, the higher centrality values correspond to ionizable residues, along the outer layer of cruzain structure. These results indicate that many residues located in the N-terminal region (β1 and α1), as well as loop_10-25_, play important roles in information transfer within the enzyme structure (Figs 17 and S20). Note that GLU35 was the residue with the highest centrality value in both apo and enzyme-substrate systems, and it also is highly conserved across the papain-like cysteine protease family. However, three positively-charged histidines located at positions 43, 106 and 115 stand out due to their occurrence among the top 10 residues with the best centrality values. Interestingly, these histidines are non-conserved within the family of papain-like cysteine proteases, which could indicate that they have a role in transmission of energetic information specifically in cruzain. In addition, LYS181 is also within the top residues with greater centralities, and its location adjacent to the catalytic ASN182 may have a great influence on the catalytic mechanism of cruzain.

On the other hand, few changes in centrality values were detected in the studied systems, the decrease of LYS17 values to almost zero being the most remarkable variation caused by ligand binding to site 3 (Fig 17). The latter event sterically hinders the interaction between LYS17 and GLU35. In consequence, the role of LYS17 in the propagation of energetic paths across cruzain structure is affected, as well as that of some other surrounding residues, i.e., ASP18 and PHE28, which increased their centrality values in various analyzed systems. Moreover, there is a noticeable decrease in centrality values of GLU73 and residues lying within the α1 helix in ligand-bound systems, i. e., ASP6, ARG8 and ARG10 (Fig 17). Note that ASP6 and ARG8 are highly conserved residues from the N-termini of these cysteine proteases [11], and the previously-mentioned changes in their local interactions may affect the structural instability of this protein regions. However, residues like LYS181, GLU122, HIS106 and PHE28, increased their centrality values in almost all complexed systems. Interestingly, the aforementioned residues are semi-conserved and non-conserved within the C1A clan, except for PHE28. This suggests that changes in interaction energies involving the previous residues upon ligand binding may perturb the function of cruzain.

## Discussion

Notwithstanding cruzain being an attractive therapeutic target for Chagas disease, few of the reported inhibitors of this protease have been selected for clinical trials in humans to date. This is probably due to their toxicity, low pharmacologic profiles and/or off-target effects. There are some aspects of this enzyme requiring further study that may foster the discovery of new drugs, such as the search for potential druggable sites to design non-competitive inhibitors by using conformational-sampling technics. So far, there are no reported studies on the identification of allosteric inhibitors of cruzain through structure-based virtual screening approaches. In this sense, our results constitute an *in silico* strategy for designing novel allosteric inhibitors of this enzyme. In the current work, several approaches of structure-based drug design were employed in order to rank the best hits that could contribute to a more specific inhibition of this protease.

The discovery of “hidden” and transient pockets, which only occur in some conformations of the protein and with no association with experimental structure, has vastly increased the number of druggable allosteric sites in these biomolecules [28, 75, 104]. Due to the difficulties of designing allosteric inhibitors targeting allosteric binding sites employing structure-based approaches, the combination between computational and experimental studies is required to explore this promising area [105]. By means of MD simulation techniques, it is possible to assess the protein conformational states, as well as the energetic penalty associated with the population shift toward the conformation required for the exposure of the hidden pocket [49, 106]. Therefore, MD simulations are particularly useful to the study of allostery, which inspired us to employ it in the present work [75, 105].

First, we performed a structural comparison of all available crystal structures of cruzain, and a few differences were found between them. Moreover, the dynamical survey of previously identified cavities showed the presence of a hidden pocket with a functional gate (site 3) and one transient pocket (site 1). Additionally, we found that the main residues of site 1 are non-conserved [11], thus making this site suitable for the design of specific allosteric inhibitors. Ultimately, the characterization of site 1 and site 3 would not have been feasible through the mere superposition of crystallographic structures of cruzain. Our study of these allosteric sites, which are potentially druggable and can modulate the enzymatic activity [37], provides a solid basis for further drug discovery.

A concrete evidence of allosteric modulation in papain-like protease family, mediated by ligand binding to site 1 and 3, is a previous study reporting several allosteric regulators of HCatK [37]. In that work, various allosteric inhibitors were predicted as compounds binding the sites 1 and 3 of the human protease. However, none of the previously-identified compounds were predicted to bind a narrow groove conformation of site 3 equivalent to that observed during cruzain MD simulations [37].On the other hand, the hypothesis that site 3 is involved in allosteric regulation and/or enzyme structural stability was previously proposed by Durrant *et al* [11]. In that work, the authors emphasized that the presence of two conserved residues lying outside site 3, i.e., SER49 and GLN51, may stabilize this groove within papain-like proteases. In addition, they underlined the role of conserved residues TYR89 and PRO90 in the rigidity of loop_84-109_ and the occurrence of a hydrogen bond network involving residues 47, 86 and 91. All these findings point out the role of site 3 in the internal stabilization of cruzain structure [11]. It is important to highlight that the “closed form” of site 3 is the most prevalent in cruzain crystal structures reported so far, since, generally, LYS17 and GLU35 are at optimal distance to form a salt bridge (~3.5 Å in crystal structures). Noteworthy, two non-conserved glutamic residues, predicted in the protonated state at pH 5.5, are present in the vicinity of LYS17 and GLU35 (Fig 9). Interestingly, the protonation states of this patch of ionizable residues at the protein surface is likely to be highly susceptible to pH variations, which, in turn, may have important functional implications. This phenomenon cannot be observed through conventional MD simulations techniques, but, in theory, it can affect the “open-close” dynamical equilibrium of this groove.

Secondly, the combination of Autodock Vina score and MM-GBSA free energy calculations allowed us to propose some compounds with the potential to bind the predicted allosteric pockets. Our results suggest that site 3 is more druggable than site 1, as none of the selected compounds from the database seemed to form a stable complex with cruzain through the latter site, as shown through long MD simulations. Therefore, the *in silico* design of ligands displaying affinity for site 1 remains challenging and the usage of larger compound databases and other transient conformations of the pocket are likely to be required. On the other hand, compounds 1 and 2 were proposed here as promising scaffolds of cruzain allosteric modulators. We verified that the aliphatic chains of the site 3 residues mediated the main protein-ligand interactions within the groove. It is important to highlight that the internal part of this pocket is formed by many aliphatic side chains, which are facing to each other, enabling the stability of secondary structures like α2 and α3 through intra-molecular hydrogen bonds (S4 and S5 Figs). Moreover, several side chains of loop_10-25_, loop_41-50_ and loop_84-109_ contribute to the stabilization of this internal structure of the groove. The disruption of the internal interaction pattern of the pocket due to the ligand binding might have a functional impact on cruzain activity.

The structural analysis of cruzain in complex with identified compounds showed slight conformational changes in enzyme binding site. However, through these structural analyses, we could also observed that compound 2 increased the flexibility of cruzain structure to a larger extent than compound 1. Remarkably, allostery can occur in the absence of well-defined conformational changes, indicating that subtle changes in protein dynamics can induce a population shift in conformational ensemble without substantially changing the mean conformation of the protein [107, 108]. The results shown here suggest that the proposed compounds would exert a modulation of the protein through the generalized allostery model, which involves the redistribution of the existing population of the conformations upon the ligand binding [26]. The modification of protein flexibility caused by the studied compounds can produce, in turn, a perturbation of enzyme processativity. Interestingly, a previous study on allosteric control in HCatK suggested that this enzyme possesses an allosteric inhibition without significant conformational changes, but through the alterations of protein motions [38]. On the other hand, the MM-GBSA calculations performed here indicated that ligand binding to site 3 moderately perturbed the substrate affinity, and that the slight differences in Δ*G*_*eff*(peptide)_ values were mainly because of modifications in the complementarity at the interfaces between S1’-S3’ subsites and the peptide positions P1’-P3’. According to the results obtained from RMSD distributions, *CP* analysis and correlation motions, the substrate binding to the orthosteric site decreases the flexibility of cruzain, thereby making the allosteric effect less appreciable upon ligand binding to site 3.

Thirdly, a community analysis of cruzain was performed, which made possible the detection of topological changes in correlated motions of this enzyme that result from ligand binding. In this analysis, we mapped the domains of papain-like cysteine proteases based on coupled movements of cruzain. As observed from our results, an increase of global correlated motions is produced when compound 2 is bound to cruzain. However, the interaction with this ligand also leads to a decrease of the “signal transduction” between the two domains of the enzyme (e.g. edges between communities 5 and 6, and between 2 and 3 when compound 2 is into site 3). A significant repartitioning among the community network stemmed from the weaker interface edges connecting the β-sheet and α-helix rich domains upon the ligand binding. In particular, we observed reorganization of communities containing the active site residues due to the presence of the compounds. This could lead to a modulation of the enzymatic activity, which requires appropriate coupled motions of the residues involved in the catalytic process. In the ternary systems (cruzain-substrate-compounds), the ligand presence into site 3 also leads to remarkable community reorganization as well as changes in the inter-domain correlated motions. This suggest the capacity of the compounds to modify the overall motions of the enzyme even when the substrate is bound to the active site, a likely signature of non-competitive inhibition.

Moreover, in the two active cruzain forms analyzed here (apo and cruzain-substrate complex), specific residues across the two domain interfaces are characterized by high centrality values, thereby suggesting their involvement in the flow and transmission of dynamical information within cruzain. These results are useful to devise experimental procedures, such as point mutations, that would yield valuable information about structure-function relationship and structural organization of cruzain and other papain-like proteases. To our knowledge, this is first work that has performed this type of study for this cysteine protease superfamily based on MD simulations data, thus providing an insight into their regulation, function and a possible pharmaceutical intervention.

The allosteric paths that link site 3 to the orthosteric site were elucidated by combining MD simulations with correlation of protein motions based in network theory. Our results showed that compound 2 triggers the propagation of signals by creating shorter pathways and stronger correlations when compared to the apo form. Furthermore, compound 2 perturbs cruzain motions in a higher degree than compound 1, pointing out that structural dissimilarities between ligands may define different communication paths, even when binding the same allosteric site. Our study also underlines the critical role of loop_10-25_ and helix 2 in allosteric communication between site 3 and catalytic site of cruzain. In this sense, previous studies on evolutionary conservation have shown that most residues of loop_10-25_ and helix 2 are partially or strongly conserved across papain-like proteases [11]. The changes in dynamical correlations observed here indicate a possible regulation of key allosteric residues of this protein family through the site 3 narrow groove. All these results evidence an effective signal propagation, which, in turn, may enable the allosteric modulation. The design of drugs interacting with key residues involved in the allosteric signal propagation and/or modifying their existing couplings, e.g., THR14, LYS17, ASP18, GLN19, CYS22, SER24, constitutes a promising approach to the modulation of cruzain activity, extensible, in principle to other papain-like proteases.

We extended our analysis to the calculation of residue-residue interaction energies separately for apo form, enzyme-substrate and compound-bound systems. Here, we included the computation of the specific residue node-betweenness centralities based on the interactions network. Therefore, we were able to identify the main residues involved in the transmission of energetic information in the active and bound states of cruzain. Indeed, our data suggest that in the cruzain-ligand forms the ionizable residues belonging to site 3 and its vicinity substantially change their pairwise energies. In this analysis, we corroborated the important role of the interaction between LYS17 and GLU35, which was the most affected one by the ligand presence. Moreover, we referred to the perturbations occurring in charged residues lying in α1, β1 and loop_10-25_ upon binding to site 3. We also underlined the role of three non-conserved histidine residues (i. e., HIS43, HIS106 and HIS115) in the connections between energetic paths within cruzain structure, as well as the structural implications of the interactions of conserved residues in modulation of papain-like cysteine proteases. Remarkably, a recent MD study proposes that allostery in the well-studied PDZ domain is driven by changes in electrostatic effects rather than solely changes in dynamics [99, 109]. Therefore, the predicted electrostatic rearrangement within the cruzain structure suggests its possible involvement in the enzyme allosteric modulation.

Remarkably, the *in silico* identification of non-competitive inhibitors is a challenging task because of various aspects. Firstly, the occurrence of transient and hidden pockets that are not observed in the available crystal structures of cruzain has to be characterized. This is a step that, when carried out *in silico*, requires experimental validation. Another major issue of the approach presented here is that the *in silico* selection of the most promising compounds was based only on their affinity for the putative allosteric pockets. However, until experimental assessment, we cannot confirm whether the selected compounds will act either as inhibitors or as simple binders unable to trigger an allosteric signal. In a worst scenario, some molecules targeting the allosteric sites might even act as activators of the enzymatic activity.

In order to understand and exploit allostery it is necessary to propose the allosteric sites, allosteric modulators and residues involved in propagating the allosteric signal. Although this work constitutes an entirely computational approximation to the discovery of allosteric inhibitors, there are various experimental techniques that can corroborate these predictions. Kinetic assays with fluorogenic substrates are usually performed in order to determine the inhibition mechanism, which, in turn, can confirm the non-competitive nature of the inhibition exerted by the proposed compounds [35, 37]. More insightful information can be obtained through the determination of the binding modes of the inhibitors using X-ray crystallography and NMR. The latter technique has also proven quite useful in the study of the allosteric mechanisms by tracking the fluctuations in ensemble populations in free and bound states, and changes in the motions of residues involved in allosteric pathways [106, 110–113]. Site-directed mutagenesis can also be used to validate the binding site and the predicted allosteric communication mechanisms [114–117].

Overall, we have characterized transient and hidden pockets in cruzain with the capacity of exerting allosteric modulation of the enzymatic activity. We also proposed some compounds displaying suitable affinity for site 3, which was the most likely druggable pocket predicted in this work. The *in silico* analyses performed here suggest that allosteric control in cruzain could be achieve due to ensemble redistribution and without major conformational changes, as has been described for other papain-like proteases. Our approach constitutes a promising way of designing novel allosteric inhibitors of cruzain.

## Supporting information

S1 Table Principal hydrogen bonds established along simulation time between cruzain allosteric site and hit compounds. (PDF)

S2 Table Distribution of cruzain residues within optimal community structures of apo and holo forms. (PDF)

S1 Text Comparison and selection of cruzain crystal structures. (PDF)

S2 Text Pairwise distance distributions identify differences in key residues of S1, S2 and S3 subsites between the apo and holo forms. (PDF)

S3 Text Small changes along PC2 detected for cruzain apo and holo forms. (PDF)

S4 Text Comparison of cross-correlation (*CC*) and generalized correlation results (*GC*). (PDF)

S1 Fig Representation of grid boxes employed in VS simulations into cruzain potential allosteric sites. (A) site 1 and (B) site 3 are highlighted in cruzain surface representation together with the corresponding docking boxes. The axis dimensions are labeled in each case and the residues contained in both sites are colored in magenta.
(TIF)

S2 Fig Comparison of crystal structures reported for cruzain. (A) Superimposed structures of cruzain obtained from PDB database. Secondary structure elements are colored as follows: alpha helices (red), beta sheets (yellow) and loops/turn (green). All ligands are positioned in the active site. (B) Pairwise RMSD calculated for the backbone atoms of cruzain crystal structures (47 in total considering each cruzain copy solved within the same PDB file). PDBID of each analyzed structure is specified in alphabetical order on the right hand side.
(TIF)

S3 Fig Comparison of the cluster central structures generated for each site by volume-based and pairwise RMSD cluster analysis. (A) Clusters (clust) calculated for site 1 (S1) and (B) site 3 (S3). Node size is proportional to average volume value and color gradient correspond to the frames number conforming each cluster. Edge color and thickness are proportional to pairwise RMSD values between the cluster central structures.
(TIF)

S4 Fig Residue composition of cruzain site 1 and site 3. Surface representation of two opposite orientations and top view of cruzain (A) site 1 and (B) site 3. Protein surface is colored according to atom type and volume size is depicted as a grid of points.
(TIF)

S5 Fig Dynamical opening/occlusion motions of site 3 gate. Snapshots of site 3 were taken at different times of cruzain apo simulations. The salt bridge formed between LYS17 and GLU35 represents the functional gate of this pocket. Cavities are colored in lightgrey and the distances between the former residues are labeled in each case.
(TIF)

S6 Fig Allosteric site stability in apo form of cruzain. (A) Time evolution of RMSD values calculated for heavy atoms of site 1 (red) and site 3 (green). Each replica was labeled with the PDBID of the cruzain structure employed as starting conformation in each case. (B) RMSF calculated for each replica of cruzain apo form. The secondary structure of cruzain is represented along the protein sequence. (C) Tube representation of free enzyme average conformation. Tube width is proportional to the per-residue atomic fluctuations computed for C-alpha atoms and the regions with high fluctuations are colored in red.
(TIF)

S7 Fig Time evolution of instantaneous Δ*G*_*eff*_ values for cruzain-compound 1 complex. Effective binding free energies of compound 1 are shown together with accumulated mean values (black lines). Dashed lines indicate the equilibration time of the MD simulations. Every replica was labeled with the corresponding average Δ*G*_*eff*_ value and its standard error of mean.
(TIFF)

S8 Fig Time evolution of instantaneous Δ*G*_*eff*_ values for cruzain-compound 2 complex. Effective binding free energies of compound 2 are shown together with accumulated mean values (black lines). Dashed lines indicate the equilibration time of the MD simulations. Every replica was labeled with the corresponding average Δ*G*_*eff*_ value and its standard error of mean.
(TIFF)

S9 Fig Time evolution of instantaneous Δ*G*_*eff*_ values for cruzain-peptide complex. Effective binding free energies of peptide are shown together with accumulated mean values (black lines). Dashed lines indicate the equilibration time of the MD simulations. Every replica was labeled with the corresponding average Δ*G*_*eff*_ value and its standard error of mean.
(TIFF)

S10 Fig Time evolution of instantaneous Δ*G*_*eff*_ values for cruzain-peptide-compound 1 complex. Effective binding free energies of compound 1 are show in grey color in order to highlight the deviation in Δ*G*_*eff*_ profiles of peptide (colored lines) in each replica. The accumulated mean values are shown as black lines. Dashed lines indicate the equilibration time of the MD simulations. Every replica was labeled with the corresponding average Δ*G*_*eff*_ value and its standard error of the mean.
(TIFF)

S11 Fig Time evolution of instantaneous Δ*G*_*eff*_ values for cruzain-peptide-compound 2 complex. Effective binding free energies of compound 2 are shown in grey color to highlight the deviation in Δ*G*_*eff*_ profiles of the peptide (colored graphs) in each replica. The accumulated mean values are shown as black lines. Dashed lines indicate the equilibration time of the MD simulations. Every replica was labeled with the corresponding average Δ*G*_*eff*_ value and its standard error of mean.
(TIFF)

S12 Fig RMSDs for the ligands and the peptide along simulation time. RMSD time profiles calculated with respect to the initial frame of MD simulations. The RMSD values for the heavy atoms of ligands are represented for the following systems (A) cruzain-compound 1, (B) cruzain-compound 2, (C) cruzain-petide-compound 1 and (D) cruzain-peptide-compound 2. The RMSD values with respect to the peptide heavy atoms are represented for (E) cruzain-peptide, (F) cruzain-peptide-compound 1 and (G) cruzain-peptide-compound 2 systems. Different colors represent an individual replica in each case.
(TIFF)

S13 Fig Distributions of pairwise interatomic distance of cruzain binding site. (A) S1, S2 and S3 region of cruzain with the interatomic distances of its principal residues. Selected residues are labeled and their side-chains are depicted as stick. (B) Distance distributions obtained from the MD simulations of three analyzed systems and from cruzain crystal structures. Graphs are labeled with the atomic pair analyzed in each case.
(TIF)

S14 Fig Comparison of contact maps of the analyzed systems. The contact map matrices are represented as a comparison of two simulated systems positioned in upper and lower triangles, respectively. Each system is identified by a different color, i. e. red for apo-form, green for cruzain-peptide, blue for cruzain-compound 1, cyan for cruzain-compound 2, magenta for cruzain-peptide-compound 1 and black for cruzain-peptide-compound 2.
(TIF)

S15 Fig Comparison of electrostatic surface of cruzain site 3. (A) Electrostatic surface representation of cruzain apo-form and (B) cruzain-compound 2 systems. Compound 2 is displayed as orange stick. The electrostatic potential ranges from -kbT/e (red) to +kbT/e (blue), where kb is the Boltzmann constant, T, the temperature and e, the electron elementary charge.
(TIF)

S16 Fig Principal component analysis (PCA) of cruzain apo and holo forms. (A) Amplitude of the first 12 eigenvectors calculated from the covariance matrix of Cα coordinates from MD simulations. (B) Percentage of total variance accounted for each of the first 12 eigenvectors. Labels beside each point indicate the cumulative fluctuation of the selected eigenvectors. The trajectory was projected onto the principal planes defined by the first two principal components. The holo form (orange) was projected onto the eigenvectors of the apo form (blue) for (C) cruzain-compound 1 and (D) cruzain-compound 2 complexes. A color gradient was employed to represent the density of structures in each region of phase space. The projections of the MD motions were represented along the first two eigenvector for (E) the apo form, (F) cruzain-compound 1 and (G) cruzain-compound 2 systems. The black arrows show the direction of collective motions (from red to green) and the principal loops are labeled.
(TIF)

S17 Fig Comparison between standard and generalized cross-correlation coefficients. Upper triangle corresponds to standard correlation and lower triangle the generalized correlation. The framed values are related to strong correlations. The violet rectangles enclose residues belonging to the catalytic core and to specificity-related subsites of cruzain. On the right side, the secondary structure elements of cruzain are numbered from N- to C-termini. This numbering scheme is also used in a linear sequence of cruzain at the bottom of the correlation matrix.
(TIF)

S18 Fig Representation of correlation networks onto cruzain structure. Each edge represents the *GC* value between a residue pair. All networks were filtered by a cut-off of *GC* values (>0.5) and the protein is colored by element of secondary structure. The edges colored in dark possess tighter correlations. This representation is shown for all analyzed systems, i. e., (A) apo-form, (B) cruzain-peptide, (C) cruzain-compound 1, (D) cruzain-compound 2, (E) cruzain-peptide-compound 1 and (F) cruzain-peptide-compound 2.
(TIF)

S19 Fig Node degeneracy in signaling pathways calculated for the analyzed systems. Each ligand-bounded complex (green lines) was compared to its reference system, respectively, i. e., cruzain apo-form (red lines) and cruzain-peptide (blue lines). The graphs are separated as follows: (A) cruzain-compound 1, (B) cruzain-compound 2, (C) cruzain-peptide-compound 1 and (D) cruzain-peptide-compound 2.
(TIFF)

S20 Fig Residue centrality obtained from PENs analysis of cruzain complexes. For convenience, the centrality profiles are represented as a comparison between reference (red lines) systems (i. e., apo form and cruzain-peptide complex) and ligand-bounded systems (blue lines) in each case. (A) cruzain-compound 1, (B) cruzain-compound 2, (C) cruzain-peptide-compound 1 and (D) cruzain-peptide-compound 2.
(TIF)
