# Supplementary material for: Dissecting a novel allosteric mechanism of cruzain: A computer-aided approach
Source: PLoS One. 2019 Jan 25;14(1):e0211227. doi: 10.1371/journal.pone.0211227 (PMC6347273; doi:10.1371/journal.pone.0211227)
Supplement: S1 Table — (PDF) [file pone.0211227.s001.pdf]

S1 Table. Principal hydrogen bonds established along simulation time between cruzain allosteric site and hit compounds.

System	Acceptor	Donor	Stability (%)	AvgDist (Å)
**Cruzain-compound 1**	Comp1_O1	GLU50_OE2	68.41	2.71
	ASN182_O	Comp1_O2	44.96	2.75
	Comp1_O3	ASN47_ND2	21.03	2.88
	Comp1_O3	ASN47_ND2	16.25	2.88
**Cruzain-compound 2**	GLU35_OE2	Comp2_O1	56.01	2.56
	GLU35_OE1	Comp2_O1	40.86	2.57
	GLU50_OE1	Comp2_N1	37.18	2.77
	GLU86_OE1	Comp2_N1	32.02	2.85
	Comp2_O1	GLU50_OE2	25.75	2.78
	ASN47_OD1	Comp2_N1	16.05	2.83
**Cruzain-peptide**	ASP161_O	PHE220_N	89.32	2.83
	GLY66_O	LEU219_N	84.32	2.84
	LEU219_O	GLY66_N	48.50	2.9
	LEU221_O	GLN19_NE2	33.73	2.87
	LEU221_O	TRP184_NE1	22.36	2.89
	ASP161_O	LEU221_N	21.18	2.92
**Cruzain-peptide-compound 1**	ASP161_O	PHE220_N	87.55	2.84
	GLY66_O	LEU219_N	75.81	2.86
	LEU219_O	GLY66_N	44.72	2.89
	LEU221_O	TRP184_NE1	29.67	2.88
	ASP161_O	LEU221_N	24.80	2.91
**Cruzain-peptide-compound 2**	ASP161_O	PHE220_N	79.92	2.83
	GLY66_O	LEU219_N	79.79	2.85
	LEU219_O	GLY66_N	43.20	2.89
	LEU221_O	TRP184_NE1	28.66	2.88
	ASP161_O	LEU221_N	25.65	2.91
	LEU221_O	GLN19_NE2	23.34	2.88
