# Supplementary material for: Dissecting a novel allosteric mechanism of cruzain: A computer-aided approach
Source: PLoS One. 2019 Jan 25;14(1):e0211227. doi: 10.1371/journal.pone.0211227 (PMC6347273; doi:10.1371/journal.pone.0211227)
Supplement: S2 Table — (PDF) [file pone.0211227.s002.pdf]

S1 Table. Distribution of cruzain residues within optimal community structures of apo and holo forms.

Comm ID	Apo	Cruzain - peptide	Cruzain-compound 1	Cruzain-compound 2	Cruzain-peptide-compound 1	Cruzain-peptide-compound 2
1	1-13, 151-152, 167-179, 195-198	1-15, 151-154, 167-179, 181, 195-198	1-13, 150-153, 167-179, 194-198	1-14, 133-135, 146-152, 165-182, 188-198	1-12, 151-153, 167-179, 195-198	1-7, 151-154, 167-179, 194-198
2	14-18, 146-150, 180-194	16-24, 92-98, 182-183	14-19, 145-149, 183-193	15-19, 183-187	13-17	8-17
3	19-22, 92-97	25-46	20-23, 51-66, 92-102	20-22, 47-59, 81-111	18-19, 180-194	18-19, 141-150, 181-193
4	23-24, 57-81	47-56, 80-91, 99-112	24-45	23, 60-80	20, 22-23, 57-67	20-24, 60-66
5	25-45	57-79	46-50, 83-91, 103-111	24-46, 112-115, 211-215	21, 92-98	25-46
6	46-56, 82-91, 98-111	113-119, 209-215	67-82	116-118, 120, 136-145, 153-164, 199-210	24-46	47-49, 81-91, 103-112
7	112-116, 210-215	120-132, 155, 199-208	112-116, 211-215	119, 121-132	47-56, 82-91, 99-111	50-59, 92-102
8	117-132	133-140, 156-166, 180	117-132, 199-200	-	68-81, 112	67-80
9	133-145, 153-166, 199-209	141-150, 184-194	133-144, 154-166, 180-182, 201-210	-	113-119, 209-215	113-118, 208-215
10					120-133, 199	119-132, 199-207
11					134-141, 154-166, 200-208	133-140, 155-166, 180
12					142-150	
