# Supplementary material for: Dissecting a novel allosteric mechanism of cruzain: A computer-aided approach
Source: PLoS One. 2019 Jan 25;14(1):e0211227. doi: 10.1371/journal.pone.0211227 (PMC6347273; doi:10.1371/journal.pone.0211227)
Supplement: S1 Text — (PDF) [file pone.0211227.s003.pdf]

S1 Tex. Comparison and selection of cruzain crystal structures

The pairwise RMSD analysis of cruzain structures showed small differences between crystal coordinates (S2 Fig). The observed deviations arise mainly from loop motions of the protein structures and not from large-scale movements. This fact indicates the stability of cruzain structure, notwithstanding the presence of dissimilar ligand scaffolds within its active site in the aforementioned structures. Consequently, the selection of the five structures for further structural analysis was based mainly on the following conditions: (i) relatively-large pairwise RMSD values, (ii) different experimental sources and (iii) high resolution (<2.5 Å).

In the 2D-RMSD matrix (S2B Fig), the structures 1-5, 16-22 and 35-47 have the greatest pairwise RMSD values. In the 1-5 range, all the structures, except for 1aim, were obtained in the same study. From the previous structures, 1ewp was selected because it has the best resolution (1.75 Å). In the 16-22 range, 3iut and 3i06 were selected since they have the best resolutions (1.2 Å and 1.1 Å respectively) and were determined in different studies. In addition, 1aim and 2aim were reported in the same work, but we selected 2aim because it has greater pairwise RMSDs. Finally, the structure 1me4 (resolution 1.2 Å) was chosen given its usage in previous simulations conducted by Durrant *et al.* [1]. Structures corresponding to 37-47 range were discarded because of their low resolution.

References

1. Durrant JD, Keranen H, Wilson BA, McCammon JA. Computational identification of uncharacterized cruzain binding sites. PLoS Negl Trop Dis. 2010;4(5):e676.
