# Supplementary material for: Dissecting a novel allosteric mechanism of cruzain: A computer-aided approach
Source: PLoS One. 2019 Jan 25;14(1):e0211227. doi: 10.1371/journal.pone.0211227 (PMC6347273; doi:10.1371/journal.pone.0211227)
Supplement: S2 Text — (PDF) [file pone.0211227.s004.pdf]

S2 Text. Pairwise distance distributions identify differences in key residues of S1, S2 and S3 subsites between the apo and holo forms

A comparison of the distribution of interatomic distances in cruzain shows some variability in the S2 and S3 regions formed by $loop_{56\text{-}67}$ and $loop_{197\text{-}208}$. In particular, some distances between residue pairs shown in S13 Fig were identified as those displaying the most appreciable differences in the active site. As can be observed, the distance distributions of the apo and holo (enzyme+allosteric inhibitor) forms have, in most cases, their respective maxima shifted towards lower values when compared to those of the crystal structures of cruzain bound to the orthosteric inhibitors. This suggests that the S3 and S2 subsites are slightly narrower when the active site is free. Therefore, the active site is likely to undergo a small structural reorganization to adapt to the molecules. On the other hand, the differences in the distance distributions of the apo and both holo forms highlight the occurrence of structural changes resulting from the binding of ligands to the putative allosteric site. The link between such dynamical differences in the active site and the inhibition of the protease remains elusive. Note, however, that a previous work [1] has emphasized the occurrence of different residue-residue distance distributions across the active site of HCatK in the apo and allosterically inhibited forms. Therefore, this phenomenon seems to be a hallmark of allosteric inhibition, and might be of major relevance for systems lacking large conformational changes during allosteric modulation.

References

1. Novinec M. Computational investigation of conformational variability and allostery in cathepsin K and other related peptidases. PLoS One. 2017;12(8):e0182387.
