# Supplementary material for: Dissecting a novel allosteric mechanism of cruzain: A computer-aided approach
Source: PLoS One. 2019 Jan 25;14(1):e0211227. doi: 10.1371/journal.pone.0211227 (PMC6347273; doi:10.1371/journal.pone.0211227)
Supplement: S3 Text — (PDF) [file pone.0211227.s005.pdf]

S3 Text. Small changes along PC2 detected for cruzain apo and holo forms

For further comprehension of cuzain correlated dynamics, PCA was performed using the Cα covariance matrix (S16 Fig). We can observe that the first 12 eigenvectors represent around 49% of total atomic fluctuations of enzyme, which means that most of total variance is not represented by only the first few eigenvectors. This is an expected result for quasi-rigid proteins (S16A and S16B Figs). For cruzain, the represented eigenvalues correspond to small and local fluctuation. Interestingly, previous works in which PCA was performed for cruzain and papain-like cysteine proteases systems did not show the statistical information of eigenvalues in terms of cumulative fluctuation accounted by them [1, 2], thus preventing the comparison with our results.

We represented our results in terms of PC1 and PC2 in cruzain apo and holo forms, since previous PCA performed for the family of papain-like cysteine proteases only considered the collective modes of these PCs to describe the most relevant motions in these enzymes [1, 2]. The representation of PC subspace as 2D histograms shows the population density of each conformational state of cruzain systems (S16C and S16D Figs). The projection of the movements of the holo states onto the apo PC2, confirms that MD conformers of apo form sampled a broader conformational space compared to the holo ones. Noteworthy, the MD simulation of the apo form is a concatenation of five replicas performed with different cruzain crystal structures. These initial conditions of MD simulation may enhance the sampling of the conformational space. In addition, it is well known that ligand binding tends to stabilize the protein structures, which could decrease the movements of great amplitude in holo forms.

The structures depicted in S16E, S16F and S16G Figs represent the extreme snapshots (extended structures) along the direction of collective motions (from red to green

direction) described by eigenvectors 1 and 2. In both systems (apo and holos), the two PC modes were associated mainly with loop motions, in accordance with previous results of per-residue RMSF values (see main article). Along the PC1 vector, the Cα of loop$_{88\text{-}109}$ undergo an open/close motion while the cruzain active site remains relatively in a fixed position. For the PC2 motion, where more dissimilarities between apo and holo were expected according to S16E, S16F and S16G Figs, only subtle differences in amplitude of the movement of 143-146 region were observed. Noteworthy, papain-like cysteine proteases contain three/four disulfide bonds which stiffen their structures. Also, the eigenvector 1 and 2 only represent two of the most dominant degrees of freedom in the configurational space, so the projected structures do not necessarily correspond to physical structures sampled during the MD simulations.

Previous works of PCA performed with papain-like cysteine proteases also denoted that the essential dynamics of these proteins lies in loop regions [1, 2]. In addition, all studies highlighted the pivotal role of loop88-109 in cruzain motions. Moreover. Hoelz *et al.* [1] reported that the identified movements for the apo form display an opening of the structure that exposes its active site (open conformation). Conversely, our results do not reproduce this behavior, which may be influenced by the conditions and structures employed in MD simulations.

References

1. Hoelz LV, Leal VF, Rodrigues CR, Pascutti PG, Albuquerque MG, Muri EM, et al. Molecular dynamics simulations of the free and inhibitor-bound cruzain systems in aqueous solvent: insights on the inhibition mechanism in acidic pH. J Biomol Struct Dyn. 2016;34(9):1969-78.

2. Novinec M. Computational investigation of conformational variability and allostery in

cathepsin K and other related peptidases. PLoS One. 2017;12(8):e0182387.
