# Supplementary material for: Dissecting a novel allosteric mechanism of cruzain: A computer-aided approach
Source: PLoS One. 2019 Jan 25;14(1):e0211227. doi: 10.1371/journal.pone.0211227 (PMC6347273; doi:10.1371/journal.pone.0211227)
Supplement: S4 Text — (PDF) [file pone.0211227.s006.pdf]

S4 Text. Comparison of cross-correlation (*CC*) and generalized correlation results (*GC*).

Comparison of cross-correlation (*CC*) and generalized correlation (*GC*) approaches shows that inter-residue correlation is strongest when using *GC* (S17 Fig). Both methods correctly identify highly coupled motions in residues belonging to substrate specificity subsites S2 and S3 (violet boxes). In addition, residues of $loop_{84\text{-}109}$ (belonging to site 1) and residues of helix 4 (conforming site 3) possess correlation values above 0.6.
