# Supplementary figures and images for: Dissecting a novel allosteric mechanism of cruzain: A computer-aided approach
Source: PLoS One. 2019 Jan 25;14(1):e0211227. doi: 10.1371/journal.pone.0211227 (PMC6347273; doi:10.1371/journal.pone.0211227)

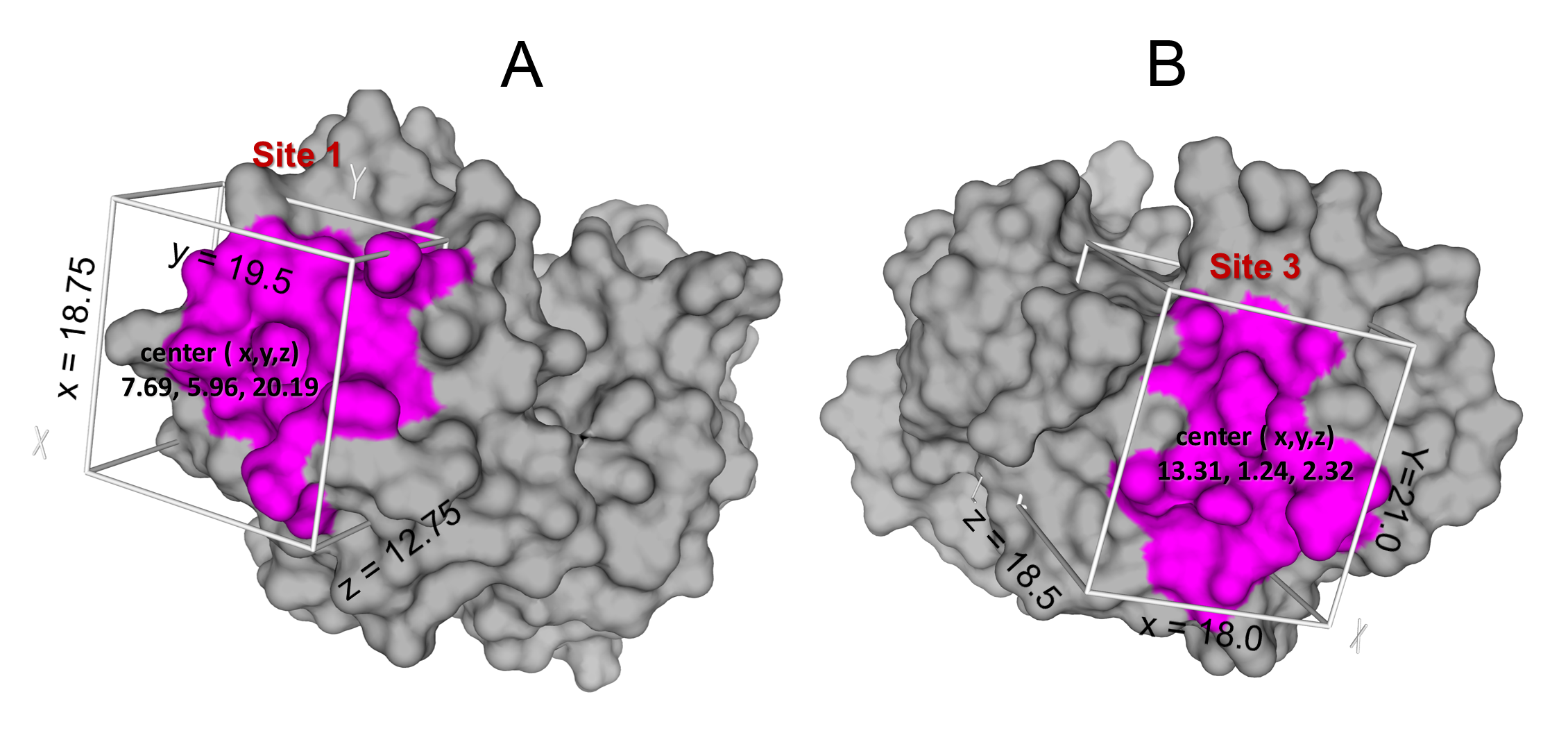

Supplement: S1 Fig — (A) site 1 and (B) site 3 are highlighted in cruzain surface representation together with the corresponding docking boxes. The axis dimensions are labeled in each case and the residues contained in both sites are colored in magenta. (TIF) [file pone.0211227.s007.tif]

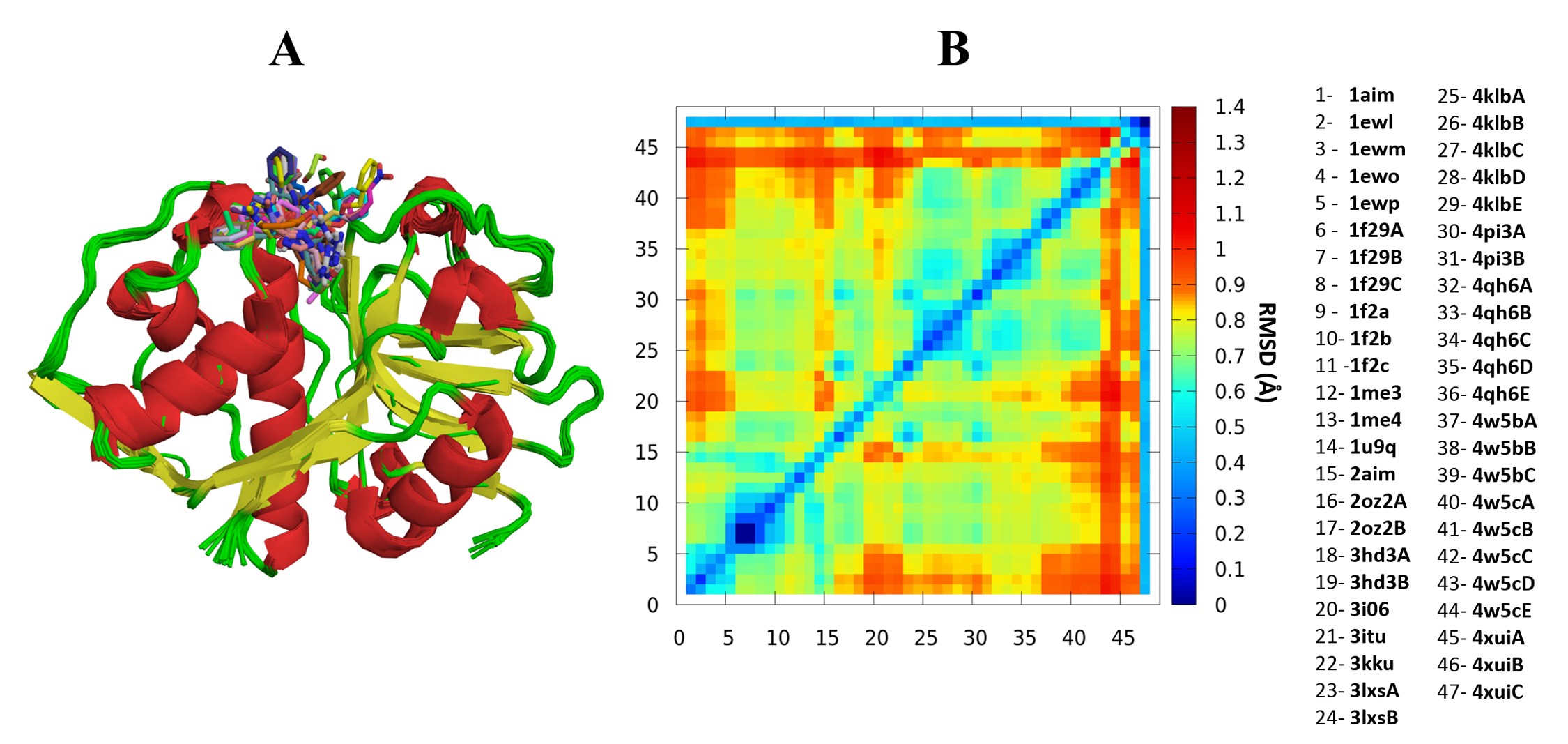

Supplement: S2 Fig — (A) Superimposed structures of cruzain obtained from PDB database. Secondary structure elements are colored as follows: alpha helices (red), beta sheets (yellow) and loops/turn (green). All ligands are positioned in the active site. (B) Pairwise RMSD calculated for the backbone atoms of cruzain crystal structures (47 in total considering each cruzain copy solved within the same PDB file). PDBID of each analyzed structure is specified in alphabetical order on the right hand side. (TIF) [file pone.0211227.s008.tif]

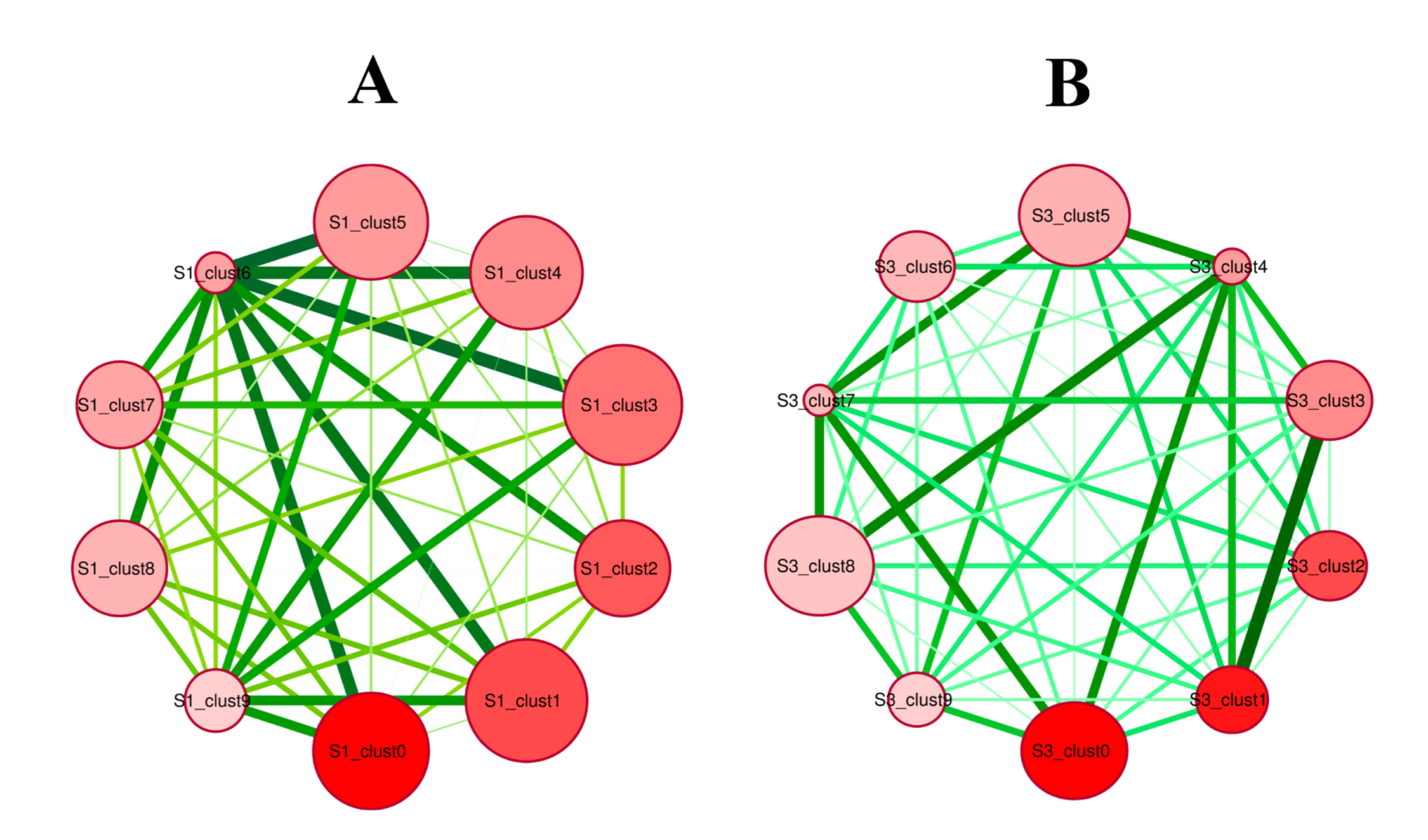

Supplement: S3 Fig — (A) Clusters (clust) calculated for site 1 (S1) and (B) site 3 (S3). Node size is proportional to average volume value and color gradient correspond to the frames number conforming each cluster. Edge color and thickness are proportional to pairwise RMSD values between the cluster central structures. (TIF) [file pone.0211227.s009.tif]

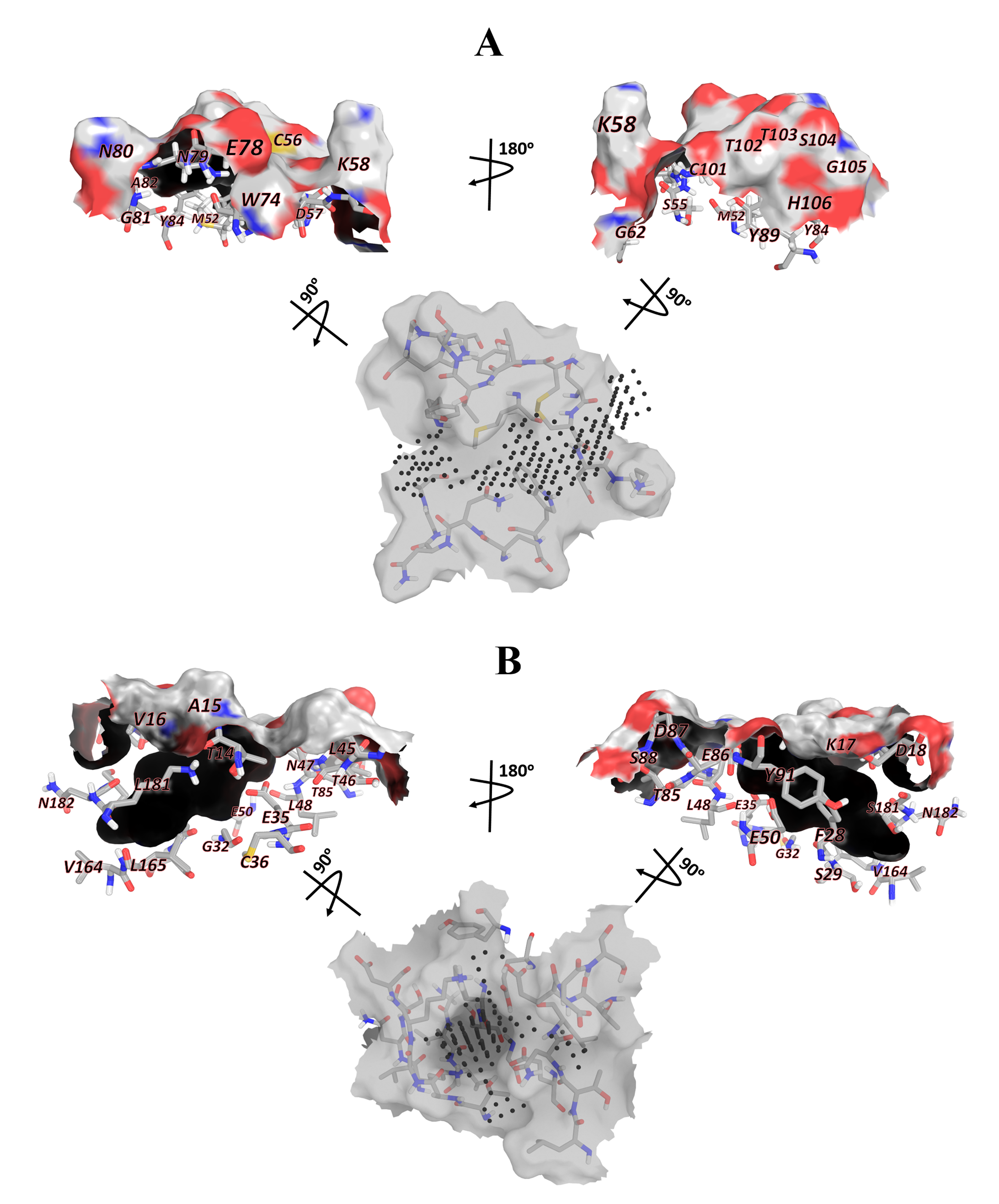

Supplement: S4 Fig — Surface representation of two opposite orientations and top view of cruzain (A) site 1 and (B) site 3. Protein surface is colored according to atom type and volume size is depicted as a grid of points. (TIF) [file pone.0211227.s010.tif]

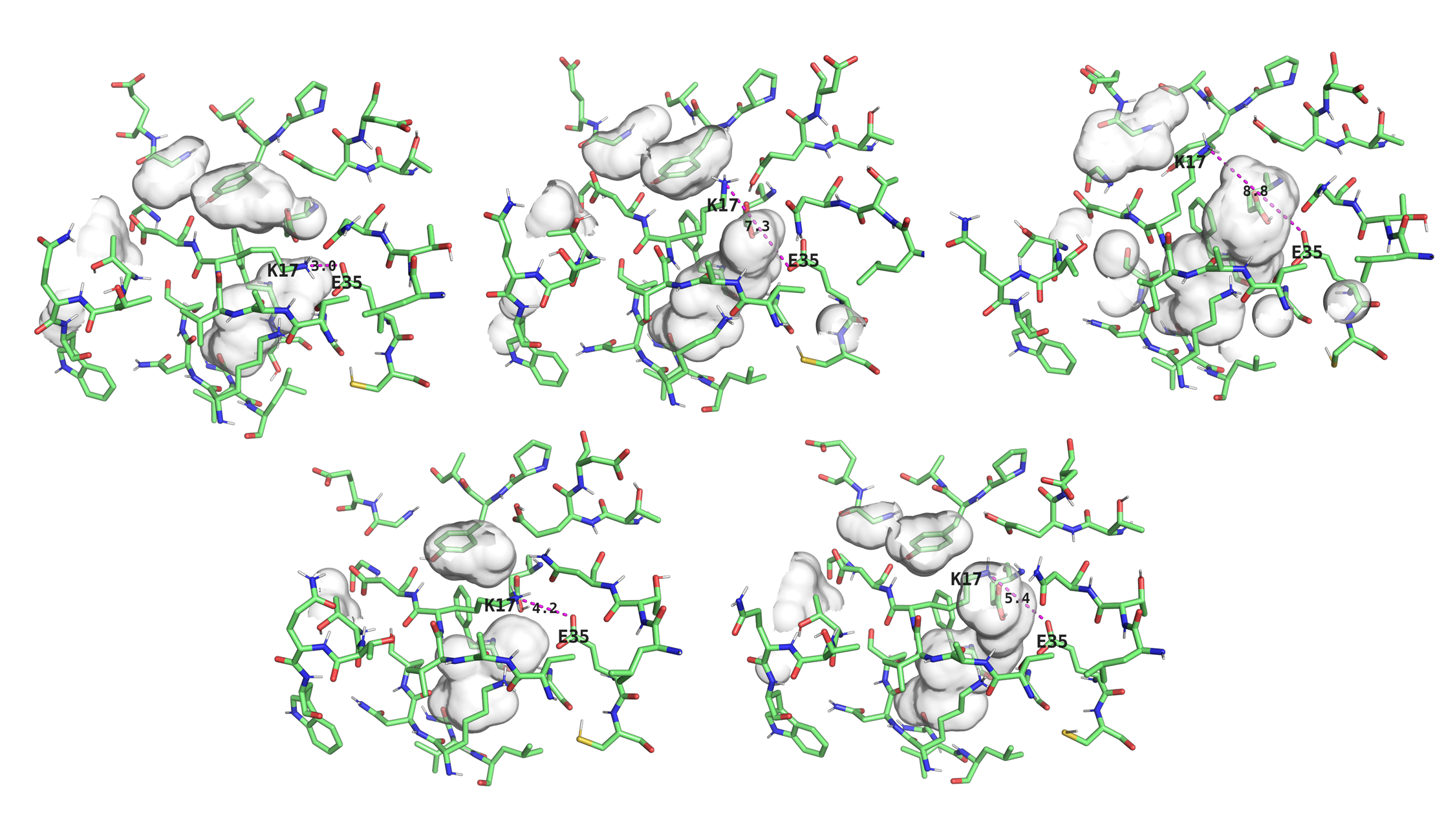

Supplement: S5 Fig — Snapshots of site 3 were taken at different times of cruzain apo simulations. The salt bridge formed between LYS17 and GLU35 represents the functional gate of this pocket. Cavities are colored in lightgrey and the distances between the former residues are labeled in each case. (TIF) [file pone.0211227.s011.tif]

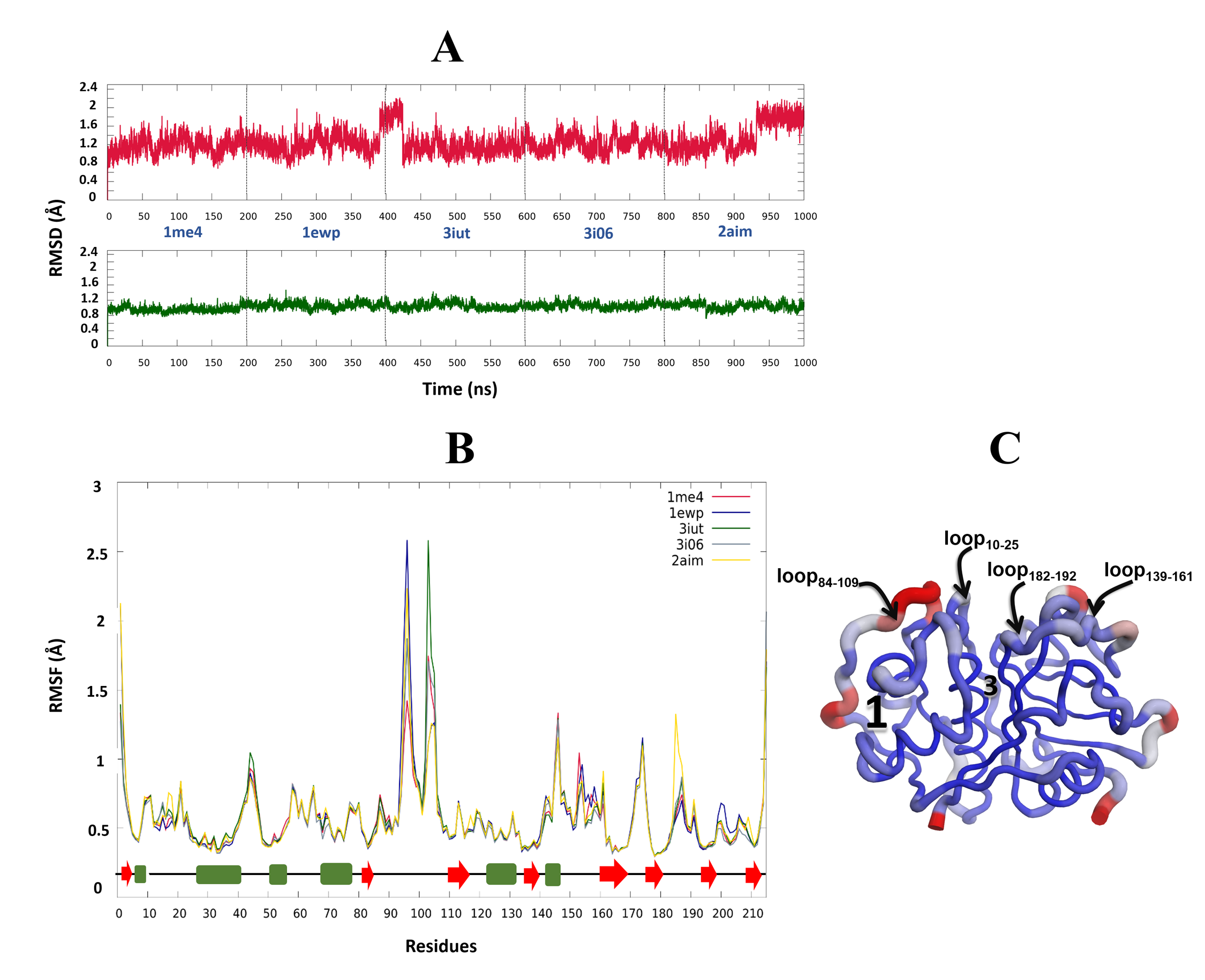

Supplement: S6 Fig — (A) Time evolution of RMSD values calculated for heavy atoms of site 1 (red) and site 3 (green). Each replica was labeled with the PDBID of the cruzain structure employed as starting conformation in each case. (B) RMSF calculated for each replica of cruzain apo form. The secondary structure of cruzain is represented along the protein sequence. (C) Tube representation of free enzyme average conformation. Tube width is proportional to the per-residue atomic fluctuations computed for C-alpha atoms and the regions with high fluctuations are colored in red. (TIF) [file pone.0211227.s012.tif]

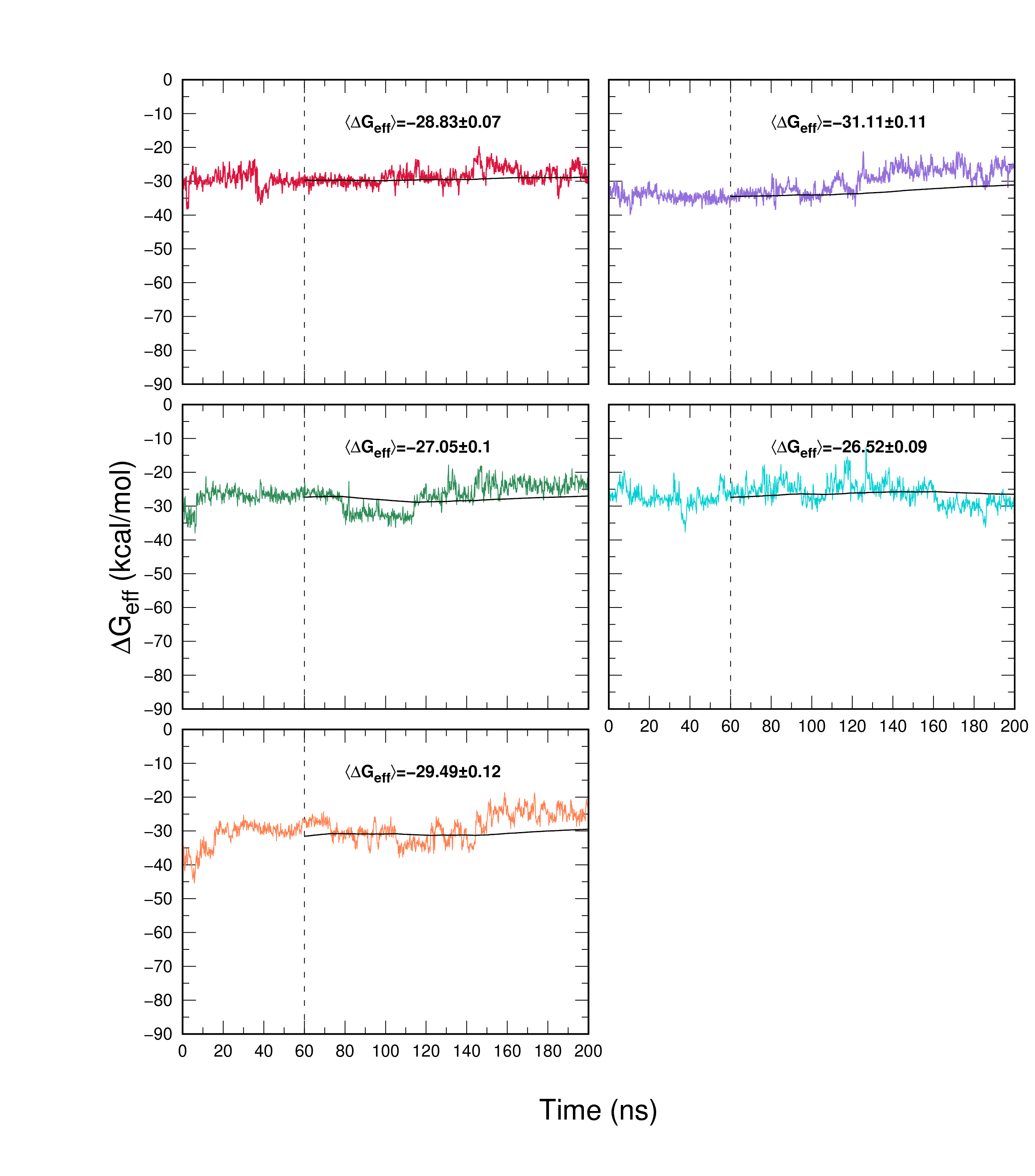

Supplement: S7 Fig — Effective binding free energies of compound 1 are shown together with accumulated mean values (black lines). Dashed lines indicate the equilibration time of the MD simulations. Every replica was labeled with the corresponding average ΔGeff value and its standard error of mean. (TIFF) [file pone.0211227.s013.tiff]

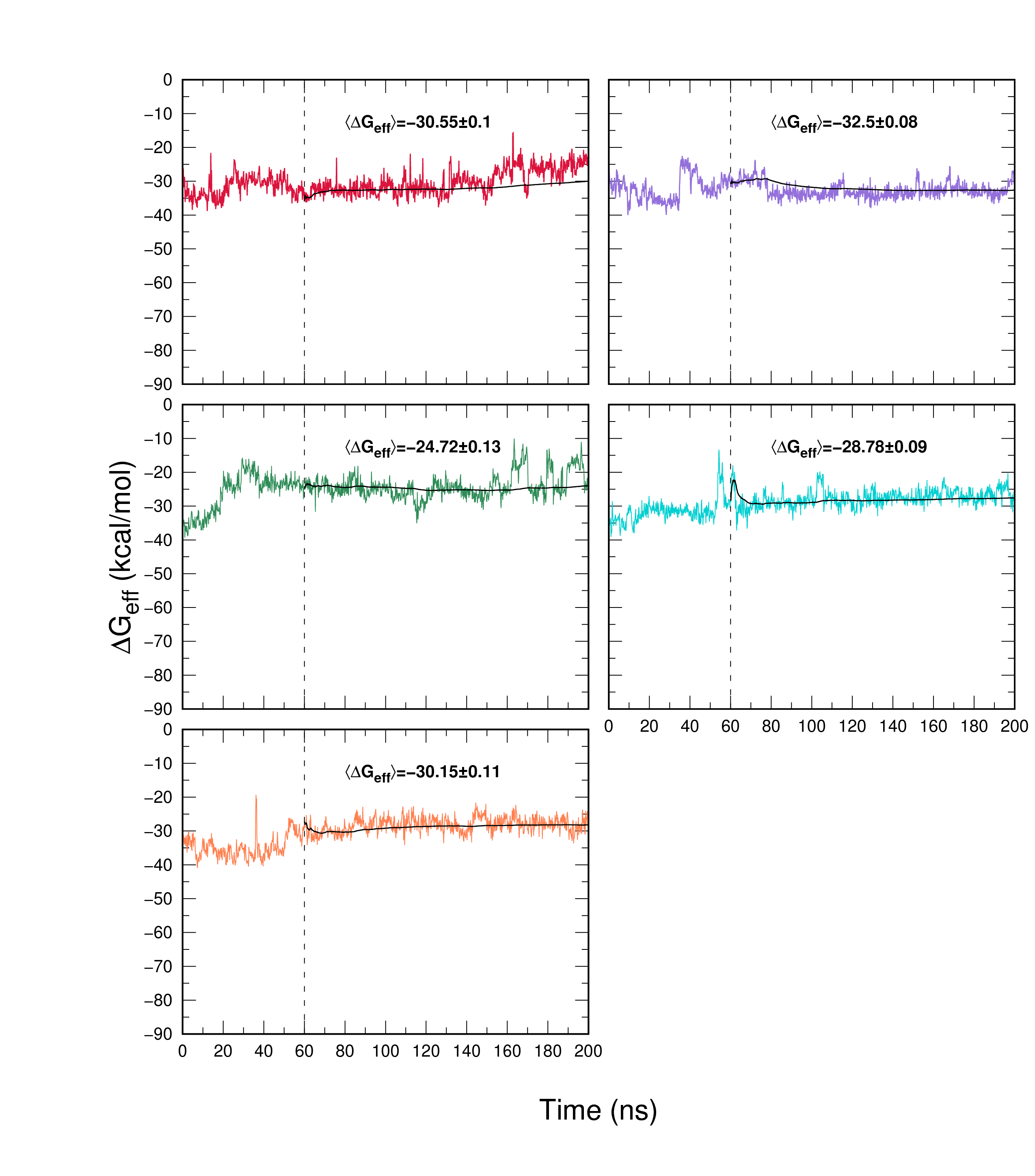

Supplement: S8 Fig — Effective binding free energies of compound 2 are shown together with accumulated mean values (black lines). Dashed lines indicate the equilibration time of the MD simulations. Every replica was labeled with the corresponding average ΔGeff value and its standard error of mean. (TIFF) [file pone.0211227.s014.tiff]

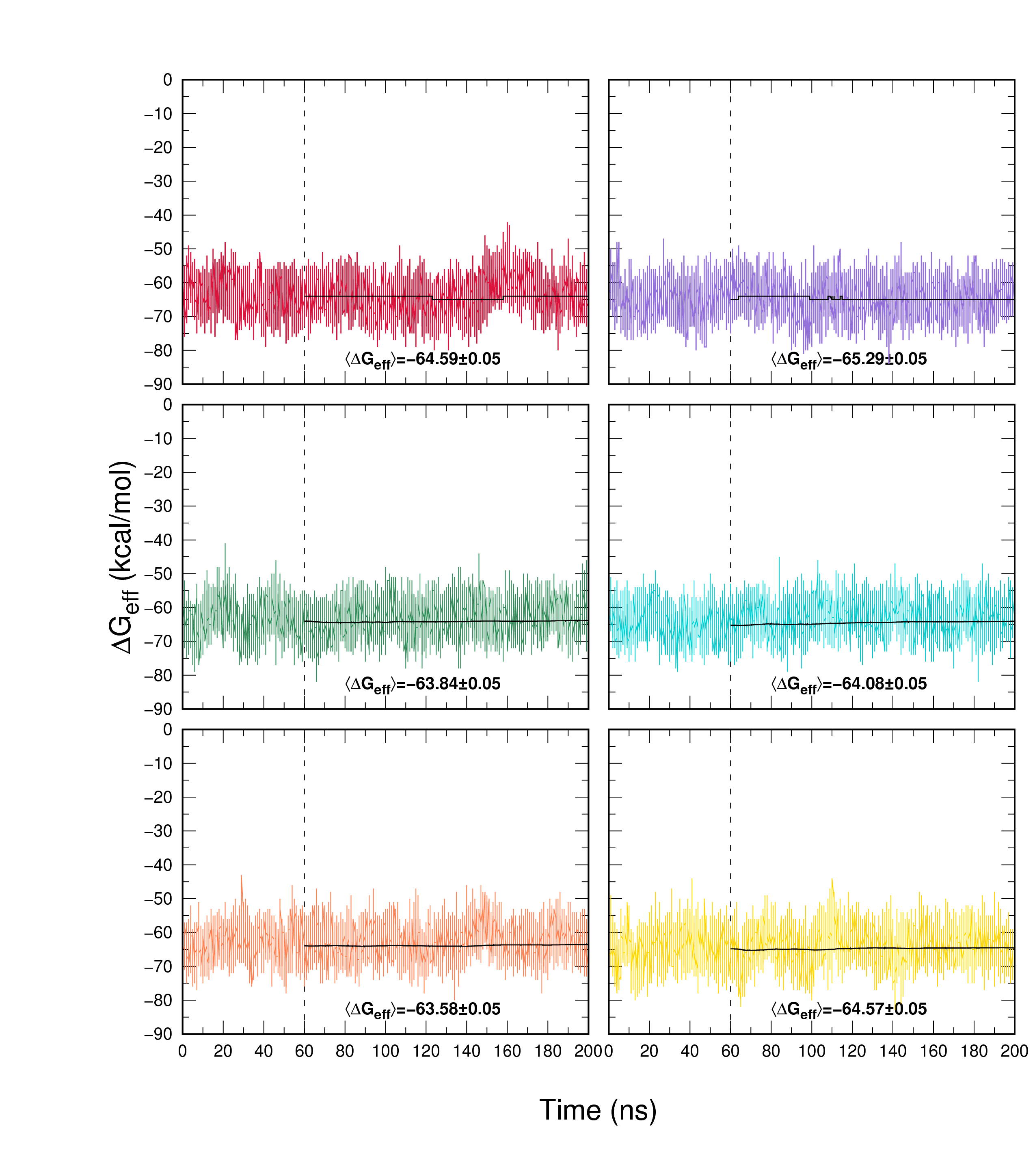

Supplement: S9 Fig — Effective binding free energies of peptide are shown together with accumulated mean values (black lines). Dashed lines indicate the equilibration time of the MD simulations. Every replica was labeled with the corresponding average ΔGeff value and its standard error of mean. (TIFF) [file pone.0211227.s015.tiff]

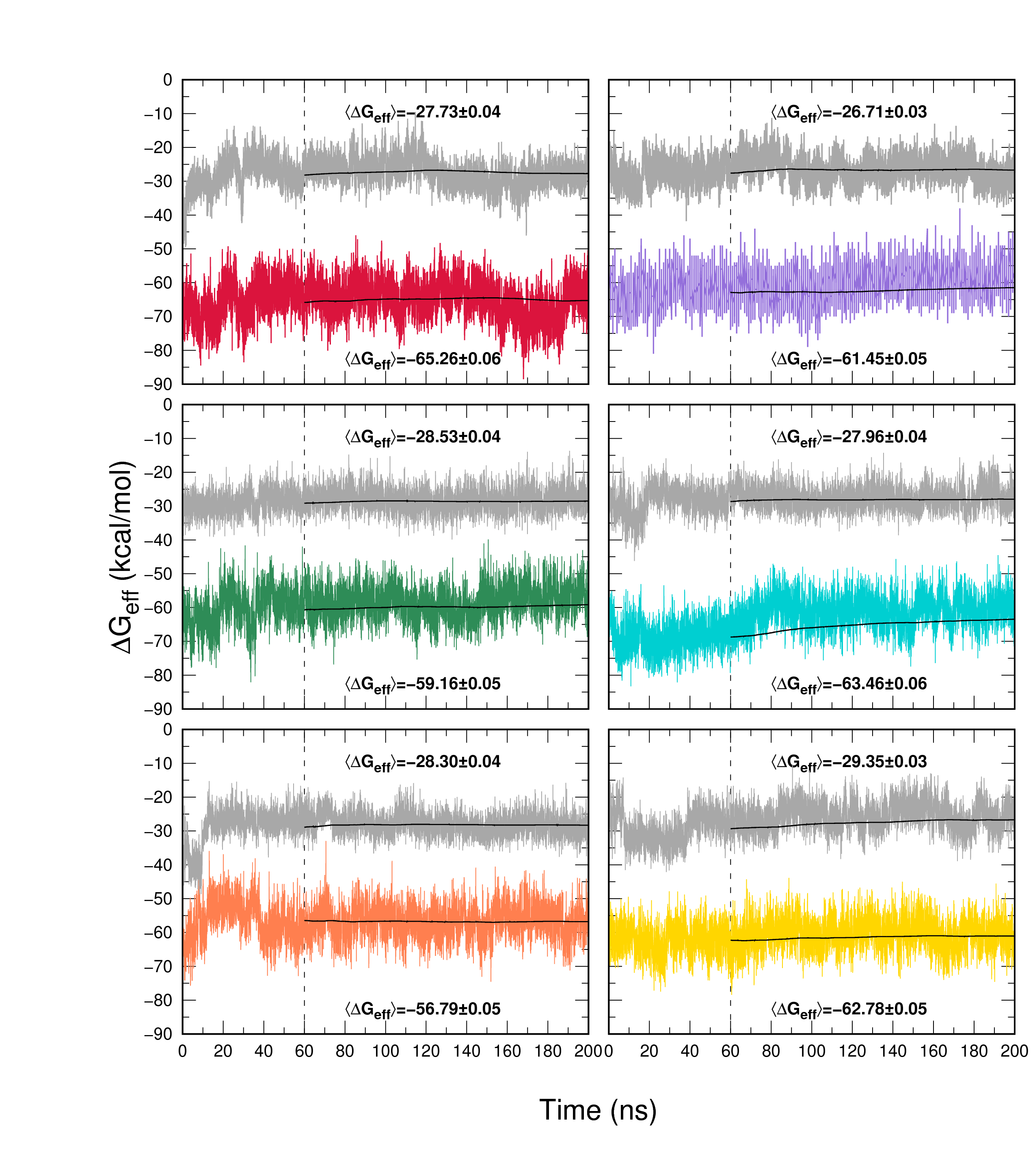

Supplement: S10 Fig — Effective binding free energies of compound 1 are show in grey color in order to highlight the deviation in ΔGeff profiles of peptide (colored lines) in each replica. The accumulated mean values are shown as black lines. Dashed lines indicate the equilibration time of the MD simulations. Every replica was labeled with the corresponding average ΔGeff value and its standard error of the mean. (TIFF) [file pone.0211227.s016.tiff]

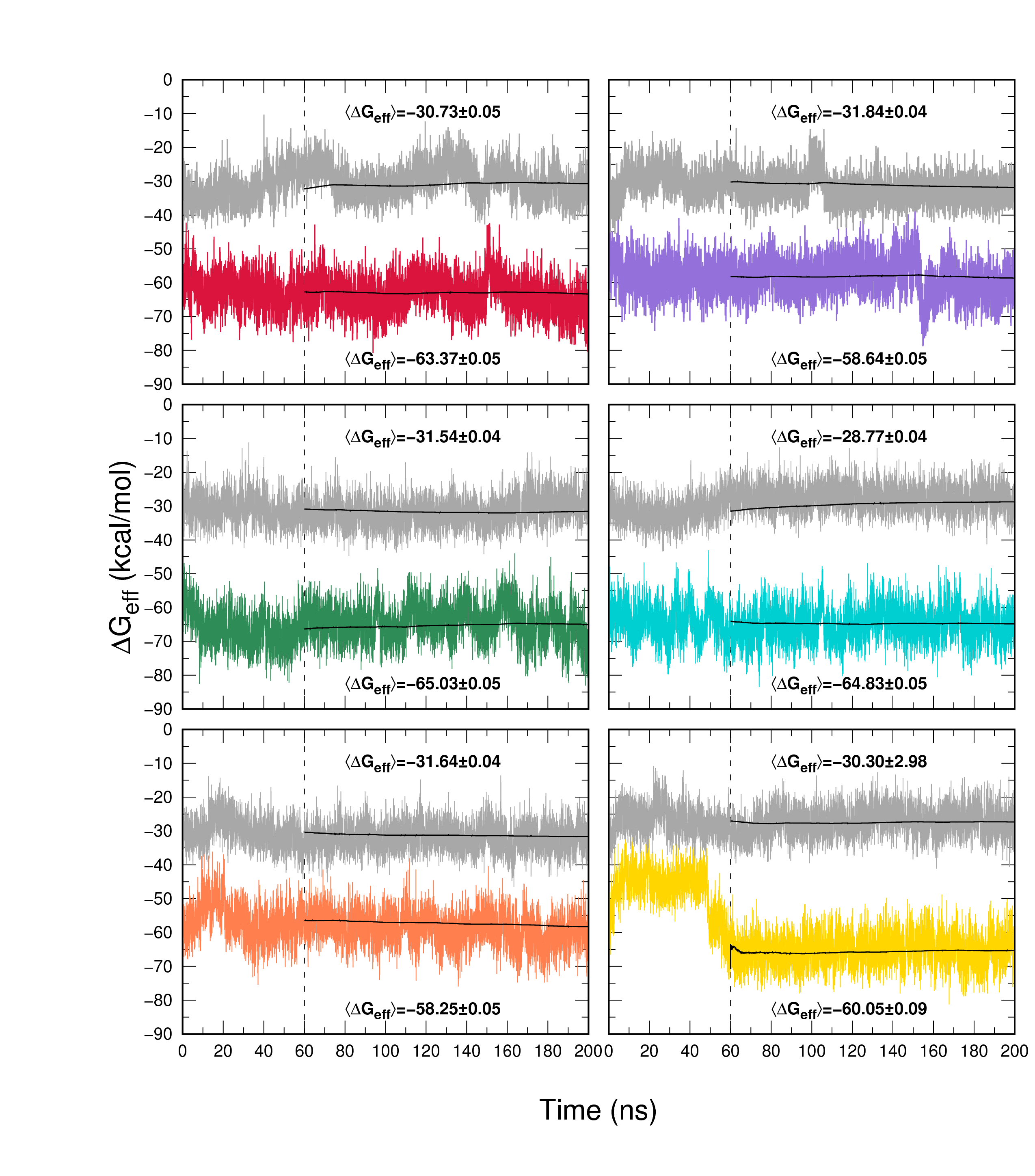

Supplement: S11 Fig — Effective binding free energies of compound 2 are shown in grey color to highlight the deviation in ΔGeff profiles of the peptide (colored graphs) in each replica. The accumulated mean values are shown as black lines. Dashed lines indicate the equilibration time of the MD simulations. Every replica was labeled with the corresponding average ΔGeff value and its standard error of mean. (TIFF) [file pone.0211227.s017.tiff]

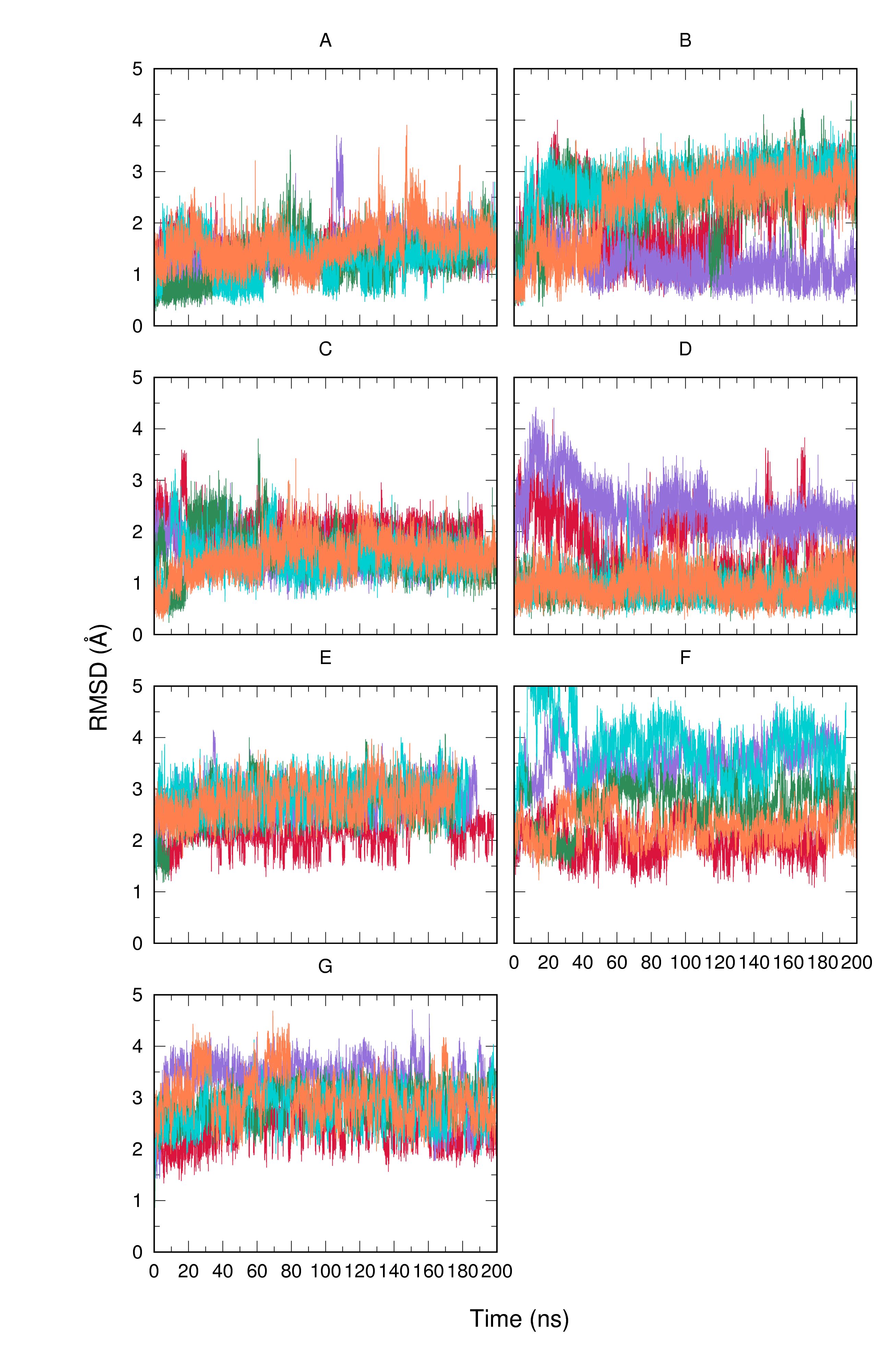

Supplement: S12 Fig — RMSD time profiles calculated with respect to the initial frame of MD simulations. The RMSD values for the heavy atoms of ligands are represented for the following systems (A) cruzain-compound 1, (B) cruzain-compound 2, (C) cruzain-petide-compound 1 and (D) cruzain-peptide-compound 2. The RMSD values with respect to the peptide heavy atoms are represented for (E) cruzain-peptide, (F) cruzain-peptide-compound 1 and (G) cruzain-peptide-compound 2 systems. Different colors represent an individual replica in each case. (TIFF) [file pone.0211227.s018.tiff]

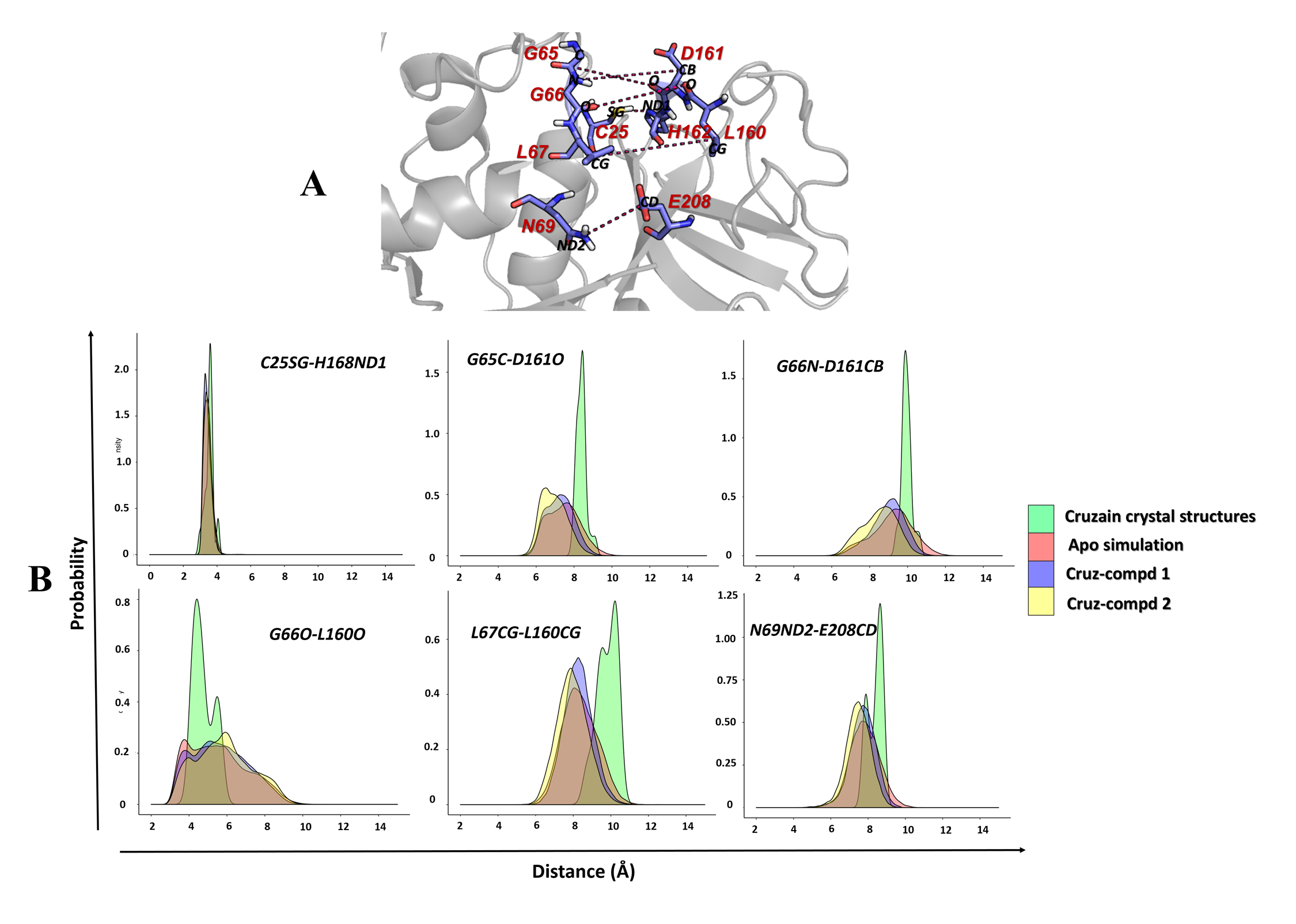

Supplement: S13 Fig — (A) S1, S2 and S3 region of cruzain with the interatomic distances of its principal residues. Selected residues are labeled and their side-chains are depicted as stick. (B) Distance distributions obtained from the MD simulations of three analyzed systems and from cruzain crystal structures. Graphs are labeled with the atomic pair analyzed in each case. (TIF) [file pone.0211227.s019.tif]

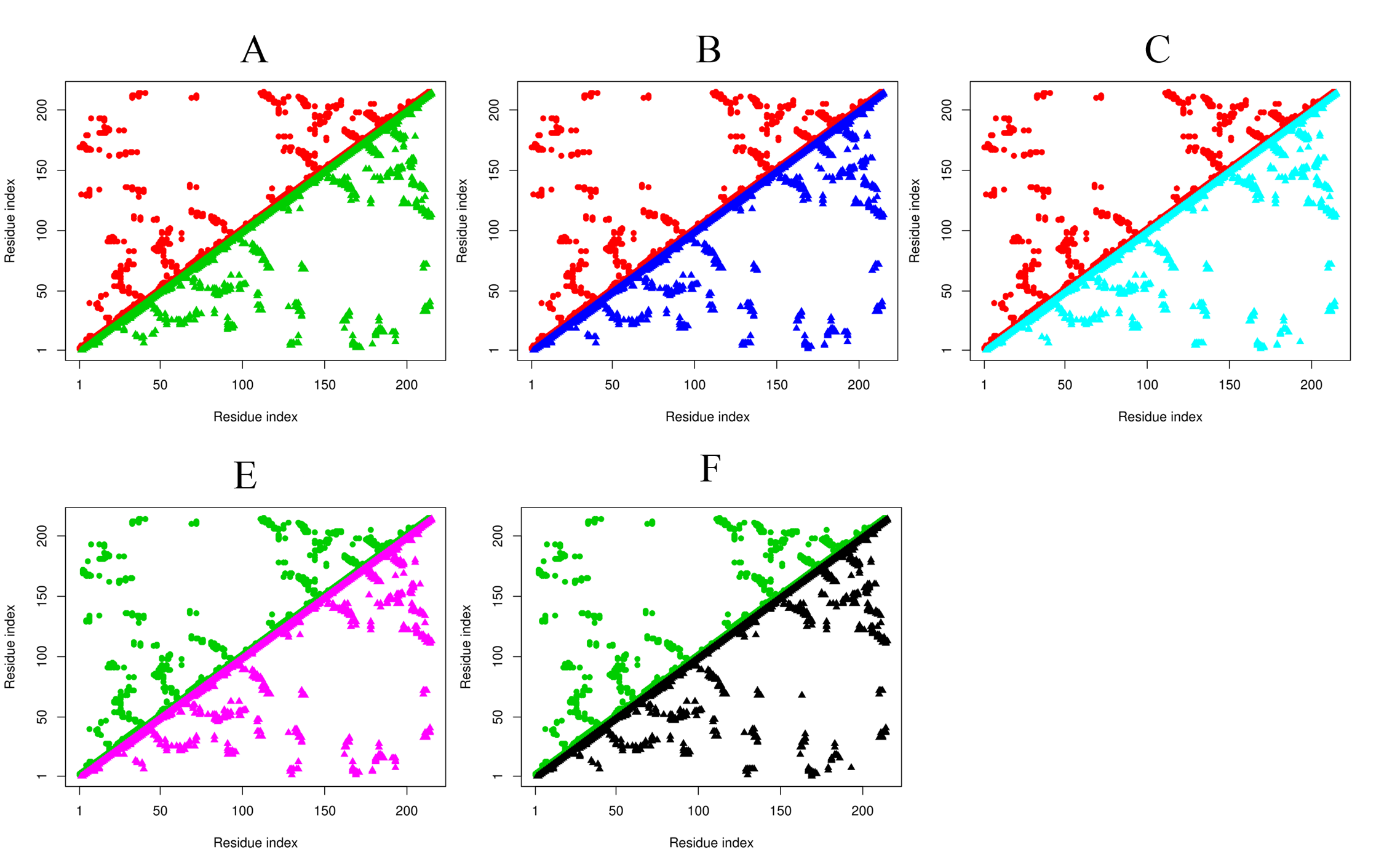

Supplement: S14 Fig — The contact map matrices are represented as a comparison of two simulated systems positioned in upper and lower triangles, respectively. Each system is identified by a different color, i. e. red for apo-form, green for cruzain-peptide, blue for cruzain-compound 1, cyan for cruzain-compound 2, magenta for cruzain-peptide-compound 1 and black for cruzain-peptide-compound 2. (TIF) [file pone.0211227.s020.tif]

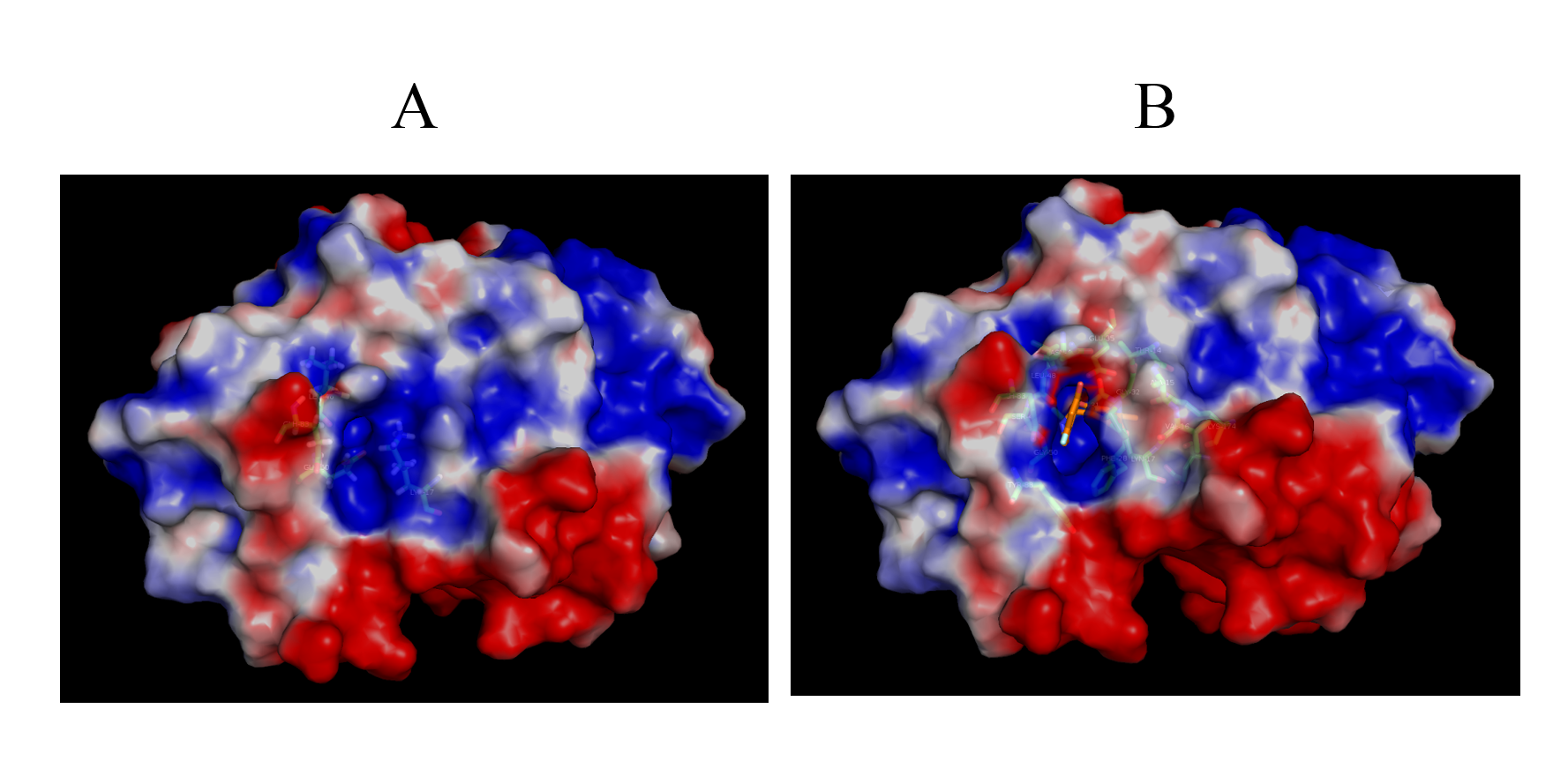

Supplement: S15 Fig — (A) Electrostatic surface representation of cruzain apo-form and (B) cruzain-compound 2 systems. Compound 2 is displayed as orange stick. The electrostatic potential ranges from -kbT/e (red) to +kbT/e (blue), where kb is the Boltzmann constant, T, the temperature and e, the electron elementary charge. (TIF) [file pone.0211227.s021.tif]

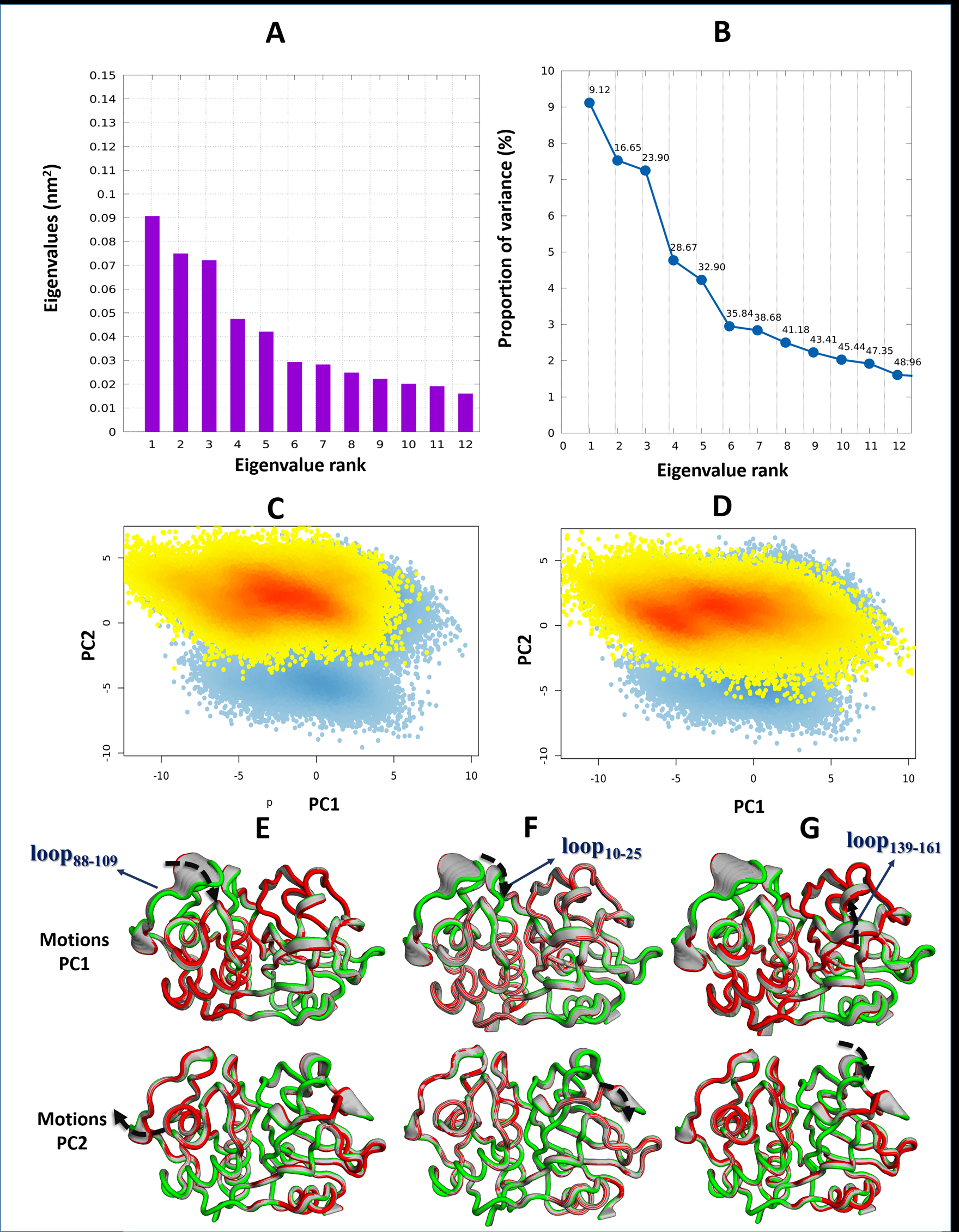

Supplement: S16 Fig — (A) Amplitude of the first 12 eigenvectors calculated from the covariance matrix of Cα coordinates from MD simulations. (B) Percentage of total variance accounted for each of the first 12 eigenvectors. Labels beside each point indicate the cumulative fluctuation of the selected eigenvectors. The trajectory was projected onto the principal planes defined by the first two principal components. The holo form (orange) was projected onto the eigenvectors of the apo form (blue) for (C) cruzain-compound 1 and (D) cruzain-compound 2 complexes. A color gradient was employed to represent the density of structures in each region of phase space. The projections of the MD motions were represented along the first two eigenvector for (E) the apo form, (F) cruzain-compound 1 and (G) cruzain-compound 2 systems. The black arrows show the direction of collective motions (from red to green) and the principal loops are labeled. (TIF) [file pone.0211227.s022.tif]

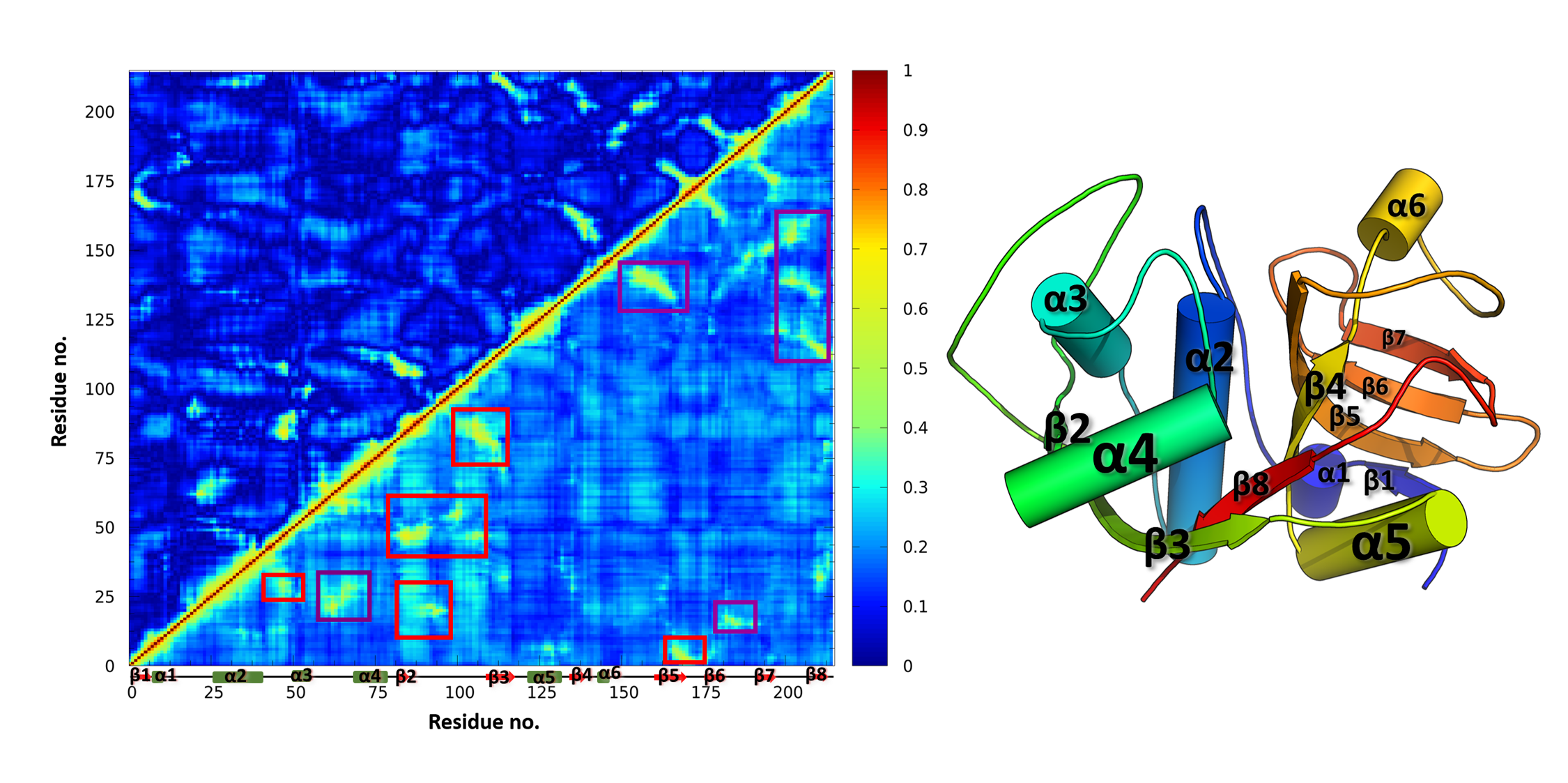

Supplement: S17 Fig — Upper triangle corresponds to standard correlation and lower triangle the generalized correlation. The framed values are related to strong correlations. The violet rectangles enclose residues belonging to the catalytic core and to specificity-related subsites of cruzain. On the right side, the secondary structure elements of cruzain are numbered from N- to C-termini. This numbering scheme is also used in a linear sequence of cruzain at the bottom of the correlation matrix. (TIF) [file pone.0211227.s023.tif]

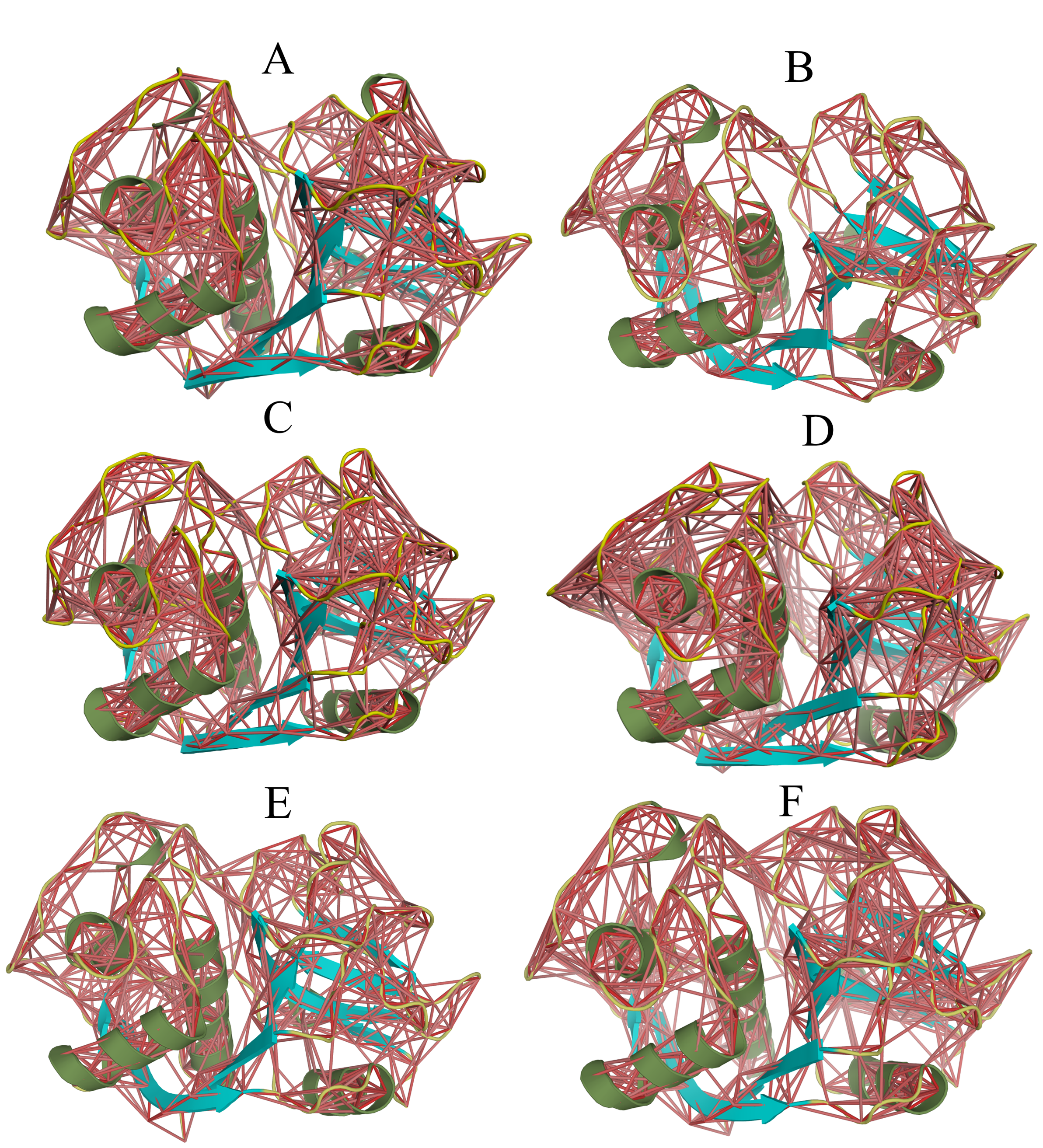

Supplement: S18 Fig — Each edge represents the GC value between a residue pair. All networks were filtered by a cut-off of GC values (>0.5) and the protein is colored by element of secondary structure. The edges colored in dark possess tighter correlations. This representation is shown for all analyzed systems, i. e., (A) apo-form, (B) cruzain-peptide, (C) cruzain-compound 1, (D) cruzain-compound 2, (E) cruzain-peptide-compound 1 and (F) cruzain-peptide-compound 2. (TIF) [file pone.0211227.s024.tif]

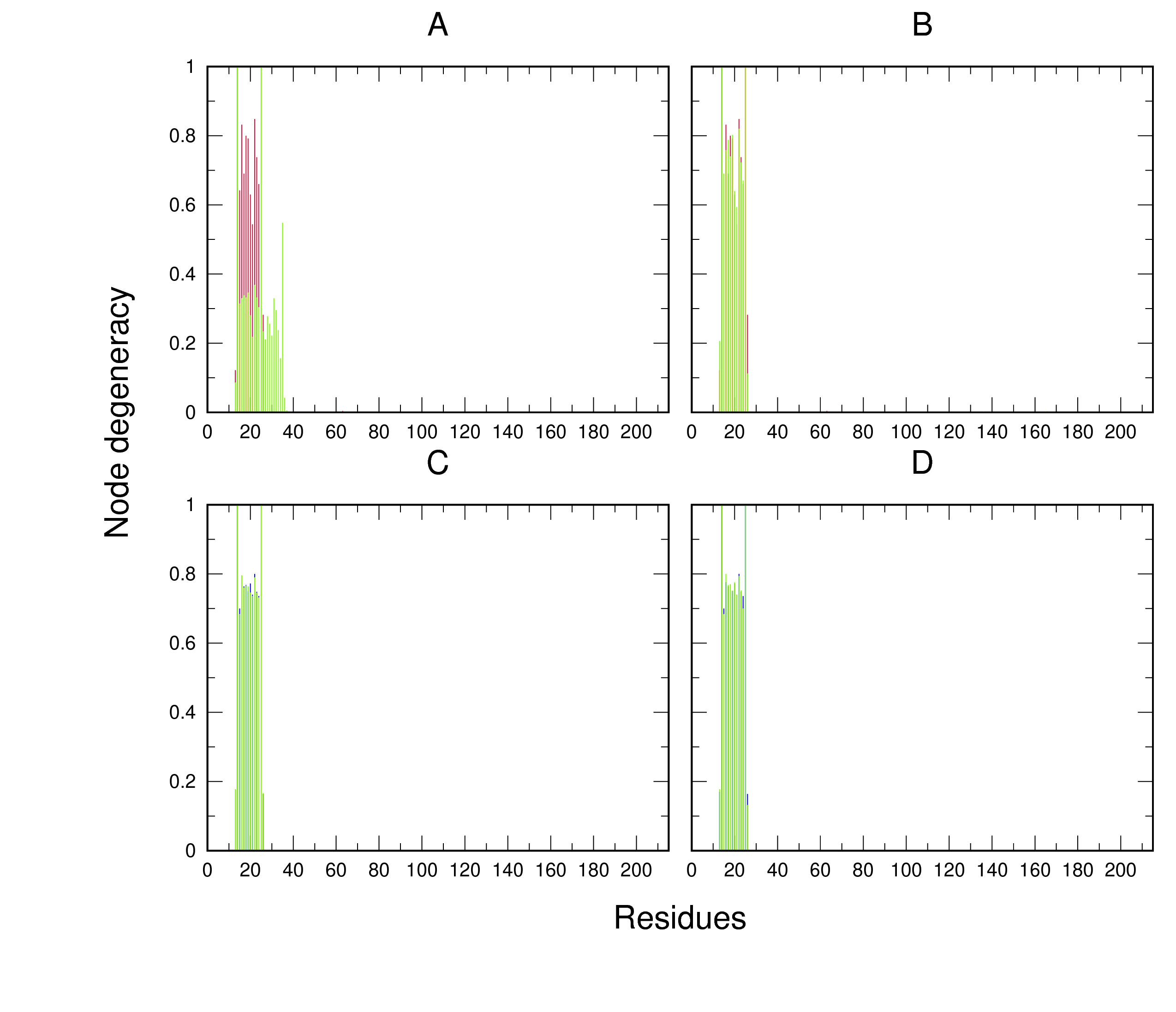

Supplement: S19 Fig — Each ligand-bounded complex (green lines) was compared to its reference system, respectively, i. e., cruzain apo-form (red lines) and cruzain-peptide (blue lines). The graphs are separated as follows: (A) cruzain-compound 1, (B) cruzain-compound 2, (C) cruzain-peptide-compound 1 and (D) cruzain-peptide-compound 2. (TIFF) [file pone.0211227.s025.tiff]

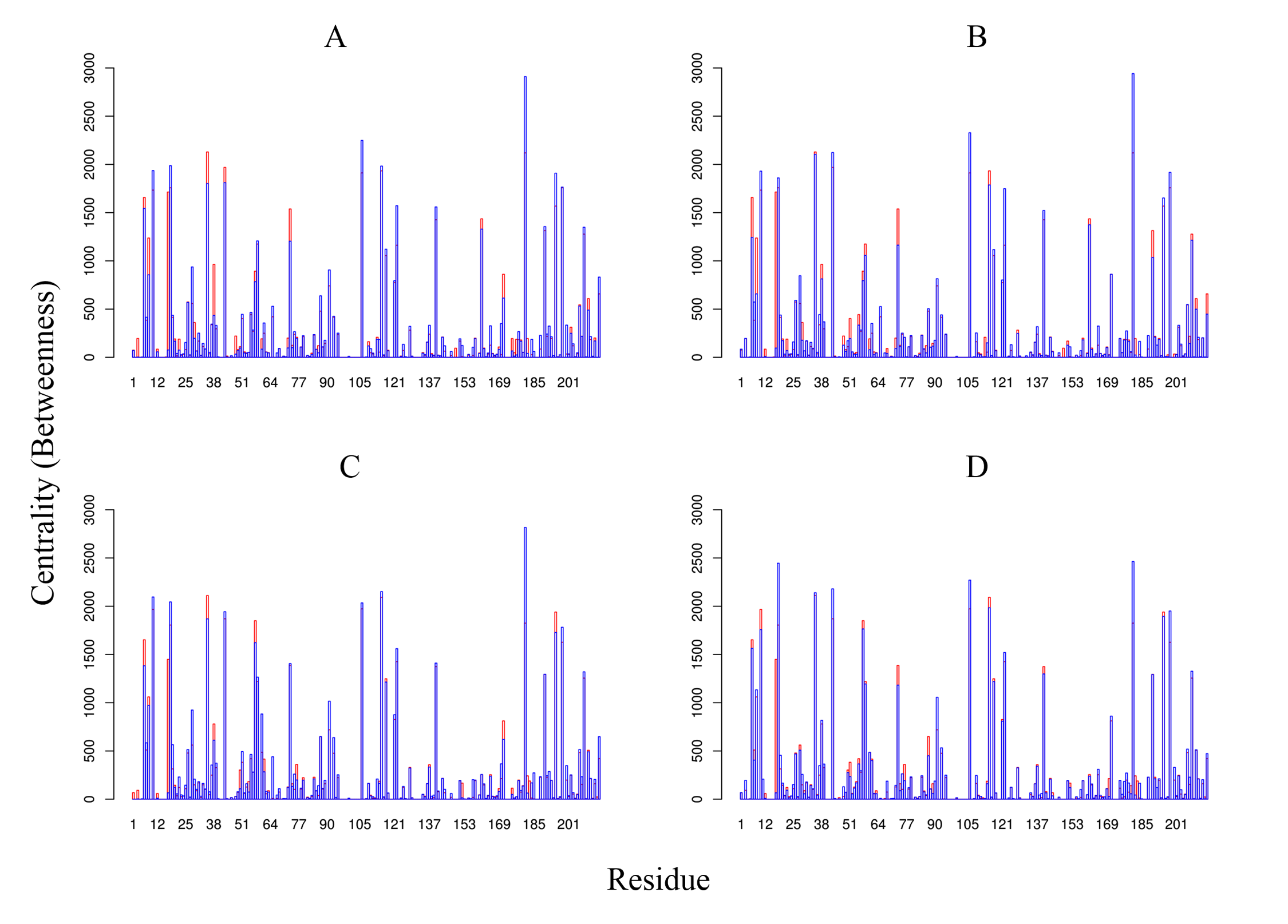

Supplement: S20 Fig — For convenience, the centrality profiles are represented as a comparison between reference (red lines) systems (i. e., apo form and cruzain-peptide complex) and ligand-bounded systems (blue lines) in each case. (A) cruzain-compound 1, (B) cruzain-compound 2, (C) cruzain-peptide-compound 1 and (D) cruzain-peptide-compound 2. (TIF) [file pone.0211227.s026.tif]
